# Screening of Potent Phytochemical Inhibitors Against SARS-CoV-2 Main Protease: An Integrative Computational Approach

**DOI:** 10.3389/fbinf.2021.717141

**Published:** 2021-10-05

**Authors:** Shafi Mahmud, Md. Robiul Hasan, Suvro Biswas, Gobindo Kumar Paul, Shamima Afrose, Mohsana Akter Mita, Mst. Sharmin Sultana Shimu, Maria Meha Promi, Umme Hani, Mohamed Rahamathulla, Md. Arif Khan, Shahriar Zaman, Md. Salah Uddin, Mohammed Rahmatullah, Rownak Jahan, Ali M. Alqahtani, Md. Abu Saleh, Talha Bin Emran

**Affiliations:** ^1^ Department of Genetic Engineering and Biotechnology, Microbiology Laboratory, University of Rajshahi, Rajshahi, Bangladesh; ^2^ Department of Genetic Engineering and Biotechnology, University of Rajshahi, Rajshahi, Bangladesh; ^3^ Department of Pharmaceutics, College of Pharmacy, King Khalid University, Abha, Saudi Arabia; ^4^ Department of Biotechnology and Genetic Engineering, University of Development Alternative, Dhaka, Bangladesh; ^5^ Department of Pharmacology, College of Pharmacy, King Khalid University, Abha, Saudi Arabia; ^6^ Department of Pharmacy, BGC Trust University Bangladesh, Chittagong, Bangladesh

**Keywords:** phytochemicals, SARS-CoV-2, molecular docking, admet, molecular dynamics

## Abstract

Coronavirus disease 2019 (COVID-19) is a potentially lethal and devastating disease that has quickly become a public health threat worldwide. Due to its high transmission rate, many countries were forced to implement lockdown protocols, wreaking havoc on the global economy and the medical crisis. The main protease (M^pro^) of severe acute respiratory syndrome coronavirus 2 (SARS-CoV-2), the causative virus for COVID-19, represent an effective target for the development of a new drug/vaccine because it is well-conserved and plays a vital role in viral replication. M^pro^ inhibition can stop the replication, transcription as well as recombination of SARS-CoV-2 after the infection and thus can halt the formation of virus particles, making M^pro^ a viable therapeutic target. Here, we constructed a phytochemical dataset based on a rigorous literature review and explored the probability that various phytochemicals will bind with the main protease using a molecular docking approach. The top three hit compounds, medicagol, faradiol, and flavanthrin, had binding scores of −8.3, −8.6, and −8.8 kcal/mol, respectively, in the docking analysis. These three compounds bind to the active groove, consisting of His41, Cys45, Met165, Met49, Gln189, Thr24, and Thr190, resulting in main protease inhibition. Moreover, the multiple descriptors from the molecular dynamics simulation, including the root-mean-square deviation, root-mean-square fluctuation, solvent-accessible surface area, radius of gyration, and hydrogen bond analysis, confirmed the stable nature of the docked complexes. In addition, absorption, distribution, metabolism, excretion, and toxicity (ADMET) analysis confirmed a lack of toxicity or carcinogenicity for the screened compounds. Our computational analysis may contribute toward the design of an effective drug against the main protease of SARS-CoV-2.

## Introduction

In Hubei province, China, the city of Wuhan identified a mysterious virus that caused respiratory illness in late December 2019 (Chan et al., 2020; Li et al., 2020b). Later, on February 11, 2020, this life-threatening virus was named severe acute respiratory syndrome coronavirus 2 (SARS-CoV-2) and was recognized as the causative agent of the coronavirus disease 2019 (COVID-19) by the World Health Organization (WHO) (Gorbalenya et al., 2020; Hu et al., 2020). Due to rapid worldwide viral transmission, on March 11, 2020, the WHO described the spread of SARS-CoV-2 as a global pandemic (Dagotto et al., 2020; Srinivasan et al., 2020). According to the latest update, on July 30, 2021, the WHO confirmed 196,553, 009 infected cases, associated with 4,200,412 deaths globally (https://covid19.who.int/). Fortunately, the death rate of COVID-19 disease is lower than that of other coronaviruses, such as SARS-CoV, which has a mortality rate approaching 9.6%, and Middle East respiratory syndrome coronavirus (MERS-CoV), which has the highest mortality rate of 35.5% (Rahman et al., 2020).

Coronaviruses are enveloped, single-stranded RNA virus with a positive sense strand that encodes an externally spherical spike protein, which presents with a crown shape and is approximately 80–160 mm in diameter (Cui et al., 2019; Li et al., 2020a; Kim et al., 2020; Yang et al., 2020). SARS-CoV-2 belongs to the suborder of Cornidovirineae within the Nidovirales order, the subfamily of Coronavirinae within the Coronaviridae family, and the genus beta-coronavirus, under the subgenus Sarbecovirus (Snijder et al., 2006; Siddell et al., 2019; Chen et al., 2020; Gorbalenya et al., 2020). Among RNA viruses, SARS-CoV-2 has the longest known genome of 26–32 kb in length and is well-formed, with a 5′ methyl-guanosine cap and 3′ poly-A tail. The genome is able to encode 9,860 amino acids (Snijder et al., 2006; Chen et al., 2020; Lu et al., 2020; Scheller et al., 2020; Yang et al., 2020; Mahmud et al., 2021c).

Bats and rodents are the primary genetic reservoir of the alpha-coronavirus and beta-coronavirus genera. Chinese horseshoe bats (*Rhinolophus* spp.) are thought to represent the most likely natural host of SARS-CoV-2. To transmit from bats to humans, SARS-CoV-2 may require an intermediate host; however, no specific evidence has been presented to support this mode of transmission. Due to a highly similar genome, Malayan pangolins (*Manis javanica*) are considered a potential intermediate host (Konda et al., 2020; Xiao et al., 2020; Ye et al., 2020). The reproduction rate of SARS-CoV-2 is much faster than that of MERS-CoV or SARS-CoV. The reproduction rate is nearly 2.5 for SARS-CoV-2, ranging from 1.8 to 3.6, compared with 2.0–3.0 for SARS-CoV and 0.9 for MERS. The average incubation periods for SARS-CoV-2 and SARS-CoV are both 5 days but can range between 2 and 14 days, whereas the incubation period for MERS-CoV lasts for 5–7 days (Alfaraj et al., 2019; Machhi et al., 2020; Petersen et al., 2020). The human immune system and the coronavirus itself potential targets for the development of COVID-19 therapies. Rather than enhancing the antiviral response of the human body, blocking viral RNA synthesis and viral self-assembly through receptor binding are considered more advantageous approaches (Omrani et al., 2014; Wu et al., 2020).

The viral main protease (M^pro^) is necessary for viral propagation and replication, making M^pro^ a promising drug target not only for SARS-CoV-2 but also for MERS-CoV, SARS-CoV, rhinoviruses, noroviruses, and enteroviruses (Naqvi et al., 2020; Sacco et al., 2020; Tripathi et al., 2020). M^pro^ is a homodimeric cysteine protease comprising 360 amino acids. Sequence alignment shows that the SARS-CoV-2 M^pro^ amino acid sequence shares 50% sequence identity with that of the MERS-CoV M^pro^, 96% identity with the SARS-CoV M^pro^, and 99% identity with the bat coronavirus RaTG13 M^pro^ (Padhi et al., 2020; Ullrich and Nitsche, 2020).

Monomeric M^pro^ can be divided into Domain I, Domain II, and Domain III, which serve as the “ceiling,” “floor,” and “basement,” respectively/Domain I consists of residues 8–101, Domain II consists of residues 102–184, and both have an anti-parallel β-barrel structure. Domain III (residues 201–303) associates with Domain II, with the 15 residues (185–200) between the two domains forming a long loop. In SARS-CoV-2, during viral replication, the formation of functional proteins through the cleavage of the polyproteins pp1a and pp1ab represents a significant step, and RdRp (RNA-dependent RNA polymerase) and nsp13-like replication-essential enzymes cannot fully function without M^pro^ protease activity (Padhi et al., 2020; Wan et al., 2020; Wu et al., 2020). The inhibition of M^pro^ during the replication process can halt the production of virus particles, making M^pro^ a desirable target for antiviral drug formulations. In the M^pro^ substrate, amino acids are arranged as (–P4–P3–P2–P1↓P1′–P2′–P3′–), from the N-terminus to the C-terminus, and multiplicity M^pro^ inhibitor at the P1 site (Du et al., 2004; Dai et al., 2020; Ullrich and Nitsche, 2020).

Since the beginning of human civilization, naturally occurring bioactive compounds with pharmaceutical potential have been derived from plants. Often referred to as secondary plant metabolites, these chemicals have functional properties that are strikingly similar to drug activities. Approximately 80% of the global population relies on natural plant-based medical treatments for their health care needs, as reported by the WHO. Among all pharmaceuticals and nutraceuticals, approximately 30–50% are derived from traditional medicinal plants (Flora and Pachauri, 2011; DW et al., 2016; Anand et al., 2019; Biswas et al., 2020). A vast range of therapeutic metabolites derived from plants are able to block viral replication or prevent cellular infection, which can inhibit the viral spread. A conventional rhinovirus infection that causes the common cold can be inhibited by the *in vitro* activity of rac-3-benzylchroman-4-ones (Khan et al., 2005; Naithani et al., 2008; Kapoor et al., 2017). Pentacyclic lupane-type triterpenoids, extracted from the aqueous portion of the plant *Cassine xylocarpa*, have been used to treat against human immunodeficiency virus (HIV). In addition, ethanol extracts of the plant *Ficus benjamina* contain kaempferol 3-O-robinobioside, kaempferol 3-O-rutinoside, and rutin compounds, which have been shown to exert antiviral effects against the herpes simplex viruses HSV-1 and HSV-2 (Yarmolinsky et al., 2012; Callies et al., 2015; Ben-Shabat et al., 2020). Glycyrrhizic acid, found in the roots of the *Glycyrrhiza radix* plant, has been shown to inhibit the Epstein-Barr virus. Decanoylphorbol-13 acetate, a phytochemical found in the leaves of the *Croton mauritianus* plant, has been used against the chikungunya virus. Flavones, such as 3′,4′-diacetoxy-5,6,7-trimethoxyflavone and naringin, are phytochemicals that have demonstrated immense efficiency against HCV, HIV, and parasitic infections. In addition, the bioflavonoid myricetin has demonstrated the remarkable ability to compete against viral infections, including influenza virus, coronavirus, and hepatitis B virus (Naithani et al., 2008; Choi et al., 2009; Corlay et al., 2014; Kapoor et al., 2017).

Antiviral agents with measurable efficacy often have dangerous side effects that can result in high morbidity and mortality, particularly when combined with viral infection. By contrast, naturally occurring bioactive substances contain phytochemicals that exert antiviral properties and can be comparably effective as alternative viral infection treatment systems with fewer negative side effects (Gasparini et al., 2012; Attia et al., 2020; Ben-Shabat et al., 2020). One example is the phytochemical baicalin, which has been used to treat enterovirus, dengue virus, respiratory syncytial virus, Newcastle disease virus, HIV, and hepatitis B virus. Another phytochemical, quercetin, can fight against adenovirus, Epstein-Barr virus, dengue virus type-2, influenza virus, poliovirus, Mayaro virus, rhinovirus, and HCV. Honokiol impedes dengue virus and HCV, genistein inhibits human cytomegalovirus, and zeaxanthin has antiviral drug efficiency against HIV (Li et al., 2000; Chiang et al., 2003; Dikici et al., 2005; Naithani et al., 2008; Li et al., 2015; Ben-Shabat et al., 2020). Therefore, phytochemicals serve as potential reservoirs of bioactive compounds with antiviral therapeutic activities that may be able to combat SARS-CoV-2 (Attia et al., 2020). Moreover, diverse natural product compounds entitled “NPC474104” (Kazinol T), “NPC306344,” “NPC470916,” “NPC173034,” “NPC66108” etc have demonstrated momentous interaction and raised as a lead compound against the SARS-CoV-2 (Muhammad et al., 2020; Rahman et al., 2020). Fleet modifying mutations of the virus genome have timbered the strait tenacious for the improvement of competent drugs and vaccines. Different countries are now using several vaccines. On December 11, 2020, a Covid-19 vaccine entitled “BNT162b2/COMIRNATY Tozinameran (INN)” was manufactured by Pfizer that granted by the FDA as the first “emergency use authorization” After that vaccine named “AZD1222” was designed by the University of Oxford and produced by AstraZeneca. In addition, a Boston-based company Moderna manufactured a vaccine “mRNA-1273” and on 18th December, FDA gave “emergency use authorization.” Besides two manufacturers Sinopharm and Sinovac launched two vaccines with the same name “SARS-CoV-2 Vaccine” at the earliest March. On March 12, 2021, Janssen (Johnson & Johnson) developed “Ad26.COV2.S” vaccine. Additionally, “Sputnik V” was invented by The Gamaleya Research Institute, part of Russia’s Ministry of Health. “Ad5-nCoV,” “EpiVacCorona” vaccines were developed by some other manufacturer. Moreover, on March 15, China endorsed a vaccine for exigence use named “ZF 2001” which has been made by two companies entitled- Anhui Zhifei Longcom and Institute of Medical Biology at the Chinese Academy of Medical Sciences as copartners. As research continues, “NVX-CoV2373” is a vaccine exhibited by a company Novavax which is Maryland-based. Furthermore, the Beijing Institute of Biological Products manufactured the “BBIBP-CorV” vaccine which is approved as exigency use by WHO on May 7. Nevertheless, the “CoronaVac” vaccine is developed by Sinovac Biotech, an unofficial Chinese company and on June 1 it gets emergency use permission from the WHO (https://www.nytimes.com/interactive/2020/science/coronavirus-vaccine-tracker.html).

Herein we have included the multiple computational algorithms to screen potent phytochemicals compounds from the in house developed library via molecular docking and molecular dynamics simulations. Also, the pharmacological profile of the screened compounds was assessed to understand their safety and efficacy level probability in lab conditions.

## Materials and Methods

### Protein Preparation

The crystalized three-dimensional (3D) structure of M^pro^ (PDB ID: 6LU7) from SARS-CoV-2 was retrieved from the RCSB Protein Data Bank (PDB) (Rose et al., 2017). The protein structure was cleaned using BIOVIA Discovery Studio (Studio, 2015) and PyMOL (DeLano, 2002). All water molecules and all hetero atoms were dispelled by PyMOL. Energy minimization was performed in GROMOS 43B1 force field I, with Swiss-PDB viewer (Kaplan and Littlejohn, 2001).

### Ligand Preparation

Initially, 1,024 compounds (Supplementary Tables S1-S2) were selected after a rigorous literature review, based on their antiviral properties, and were retrieved from the PubChem database (Kim et al., 2016). The ligand structure was prepared, and energy was minimized using the mmff94 force field (Molecular et al., 1996) along with the steepest descent optimization algorithm.

### Computational Molecular Docking Analysis

For a better understanding of the binding affinities and interactions between M^pro^ and ligands, molecular docking analysis was performed in association with the AutoDock software version 4.2 (Morris et al., 2012). Each ligand was converted into an acceptable PDBQT format for AutoDock. The energy was minimized by a universal force field (UFF). The PROPKA performs the pKa calculations in this docking study and adjust the protonation state of the targets (Hui Li and Jensen, 2005). PROPKA uses the 3D structure of proteins and protein-ligand complexes to estimate the pKa values of ionizable groups. The adjustments were made assuming the physiological pH of around 7.0; this is because some complexes are formed at low physiological pH and other exist at high physiological pH. The all tautomer states were generated and calculated independently. The stereoisomer generator mcule converts unknown or undefined tetrahedral stereocenters and *cis*-trans double bonds into well-defined centers and double bonds. In AutoDock, a grid box was generated, in which the center of the grid box was X: 26.299; Y: 12.6039; Z: 58.9455, and dimensions, in angstrom, were X: 50.3334; Y: 67.2744; Z: 59.2586. Docking was performed using the Lamarckian Algorithm, and the parameters were set to 250 runs and 25,000,000 energy evaluations for each cycle. The exhaustiveness was set as 8. The co-crystalized ligand (PDB: 6LU7) were used as a control where the ligand molecules were removed by Discovery studio and docked against the M^pro^ by using the same protocols. The binding affinities of ligands are displayed as negative scores, kcal/mol, in which more negative scores reflect better binding affinity. PyMOL and BIOVIA Discovery Studio were used to verify non-bond interactions.

### ADME/T

To evaluate the pharmacokinetic properties of the compounds, three feasible online servers, SwissADME (Daina et al., 2017), admetSAR (Cheng et al., 2012), and pKCSM (Pires et al., 2015), were used. The canonical simplified molecular-input line-entry system (SMILES) of the screened complexes was used in the entry system.

### Biological Activities of the Drug Candidates

A cheminformatics tool, Molinspiration (https://www.molinspiration.com/), was used to predict the specific biological activities of the selected hit compounds. The retrieved Canonical SMILES of the screened reliable compounds were incorporated to assay the biological activities.

### Molecular Dynamics Simulation

Molecular dynamics simulations of the docked complexes and the control were assessed to determine the overall stability of the complex in atomistic simulation conditions. The simulation study was performed in the YASARA dynamics package (Land and Humble, 2018) with an AMBER14 force field (Case et al., 2005). The cubic simulation cell was created and extended to 20 Å on each side of the complex. The ligand was parameterized by AutoSMILE algorithms, which used combined, AM1BCC and General AMBER Force Field (GAFF) for assigning atomic charges (Stewart, 1990; Jakalian et al., 2002; Wang et al., 2004). The complex was initially cleaned and optimized, along with hydrogen bond orientations. The initial energy minimization process was conducted using the steepest gradient algorithms by simulated annealing methods. The TIP3P water solvation model was used at conditions of 0.997 g/L^−1^, 25°C, and 1 atm (Krieger and Vriend, 2015). The total physiological conditions of the simulation cell were neutralized by the addition of 0.9% NaCl at 310 K, pH 7.4. The long-range electrostatic interactions were calculated by the particle mesh Ewalds algorithms, using a cutoff radius of 8 Å. The simulation time step was set to 1.25 fs (Krieger et al., 2006). Simulation snapshots were saved every 100 ps and finally run for 100 ns Finally, the simulation trajectories were used to calculate the root-mean-square deviation (RMSD), root-mean-square fluctuation (RMSF), the radius of gyration (Rg), solvent-accessible surface area (SASA), and hydrogen bonds of the complexes (Islam et al., 2019; Mahmud et al., 2020a, Mahmud et al., 2020b; Khan et al., 2020; Bappy et al., 2020; Samiul Islam et al., 2020; Swargiary et al., 2020; Mahmud et al., 2021a, Mahmud et al., 2021b, Mahmud et al., 2021d; Afrose et al., 2021; Chowdhury et al., 2021; Pramanik et al., 2021).

All of the simulation’s snapshots were further used for the binding free energy calculation through (MM-PBSA) by YASARA software using the following formula.

Binding Energy = E_potRecept_ + E_solvRecept_ + E_potLigand_ + E_solvLigand_ -E_potComplex_- E_solvComplex_

In these calculations, YASARA built-in macro files were used for MM-PBSA binding free energy where more positive energy indicates better binding (Dash et al., 2019).

## Results

### Molecular Docking Analysis

**FIGURE 1:**
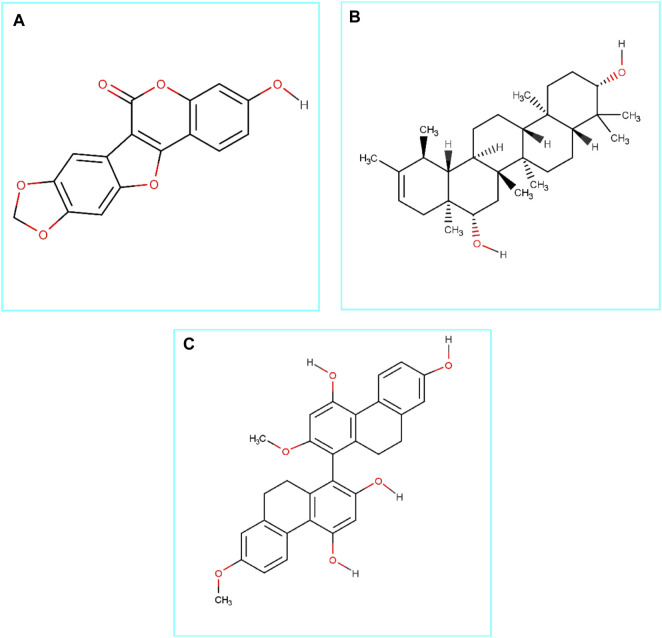
Chemical structures (2D) of Medicagol **(A)**, Faradiol **(B)**, and Flavanthrin **(C)**. The structures were drawn using Marvin Sketch software.

Based on the molecular docking analysis results, the best three (medicagol, faradiol, and flavanthrin) molecules (Figure 1 and Supplementary Table S3) were selected. The selected molecules, medicagol, faradiol, and flavanthrin, exhibited binding energies of −8.3, −8.6, and −8.8 kcal/mol, respectively whereas the control complex had binding energy of −8.0 kcal/mol **(** Table 1 **)**.

**TABLE 1:** Non-bond interactions between SARS-CoV-2 main protease and the top three compounds.

PubChem CID	Compounds	Binding affinity (kcal/mol)	Residue in contact	Interaction type	Distance in Å
5,319,322	Medicagol	−8.3	TYR54	Conventional Hydrogen Bond	2.55
			ASN142	Carbon Hydrogen Bond	2.59
			HIS41	Pi-Pi Stacked	5.72
			HIS163	Pi-Pi T-shaped	5.68
			CYS145	Pi-Alkyl	5.49
			MET165	Pi-Alkyl	5.18
			MET49	Pi-Alkyl	4.29
9,846,222	Faradiol	−8.6	THR24	Conventional Hydrogen Bond	2.93
			GLN189	Carbon Hydrogen Bond	2.42
			MET49	Alkyl	3.87
			MET165	Alkyl	4.23
			CYS145	Alkyl	5.30
			HIS41	Pi-Alkyl	5.41
102,004,681	Flavanthrin	−8.8	CYS145	Conventional Hydrogen Bond	2.96
			GLU166	Conventional Hydrogen Bond	2.52
			THR190	Conventional Hydrogen Bond	1.78
			GLY143	Conventional Hydrogen Bond	2.22
			MET49	Pi-Sulfur	5.72
			MET165	Alkyl	4.80
Control		−8.0	GLU166	Conventional Hydrogen Bond	1.93
			THR190	Conventional Hydrogen Bond	2.59
			GLN189	Conventional Hydrogen Bond	1.95
			MET165	Carbon Hydrogen Bond	2.72
			HIS164	Carbon Hydrogen Bond	2.95
			MET49	Alkyl	4.66
			LEU167	Alkyl	5.46
			HIS41	Pi-Alkyl	4.31

Medicagol, when bound to M^pro^, formed one conventional hydrogen bond at Tyr54, one carbon-hydrogen bond at Asn142, one pi-pi-stacked bond at His41, one pi-pi T-shaped bond at His163, and three pi-alkyl bonds at Cys145, Met165, and Met49.

The faradiol and M^pro^ interaction was stabilized by one conventional hydrogen bond at Thr24, one carbon-hydrogen bond at Gln189, three alkyl bonds at Met49, Met165, and Cys145, and one pi-alkyl bond at His41.

**FIGURE 2:**
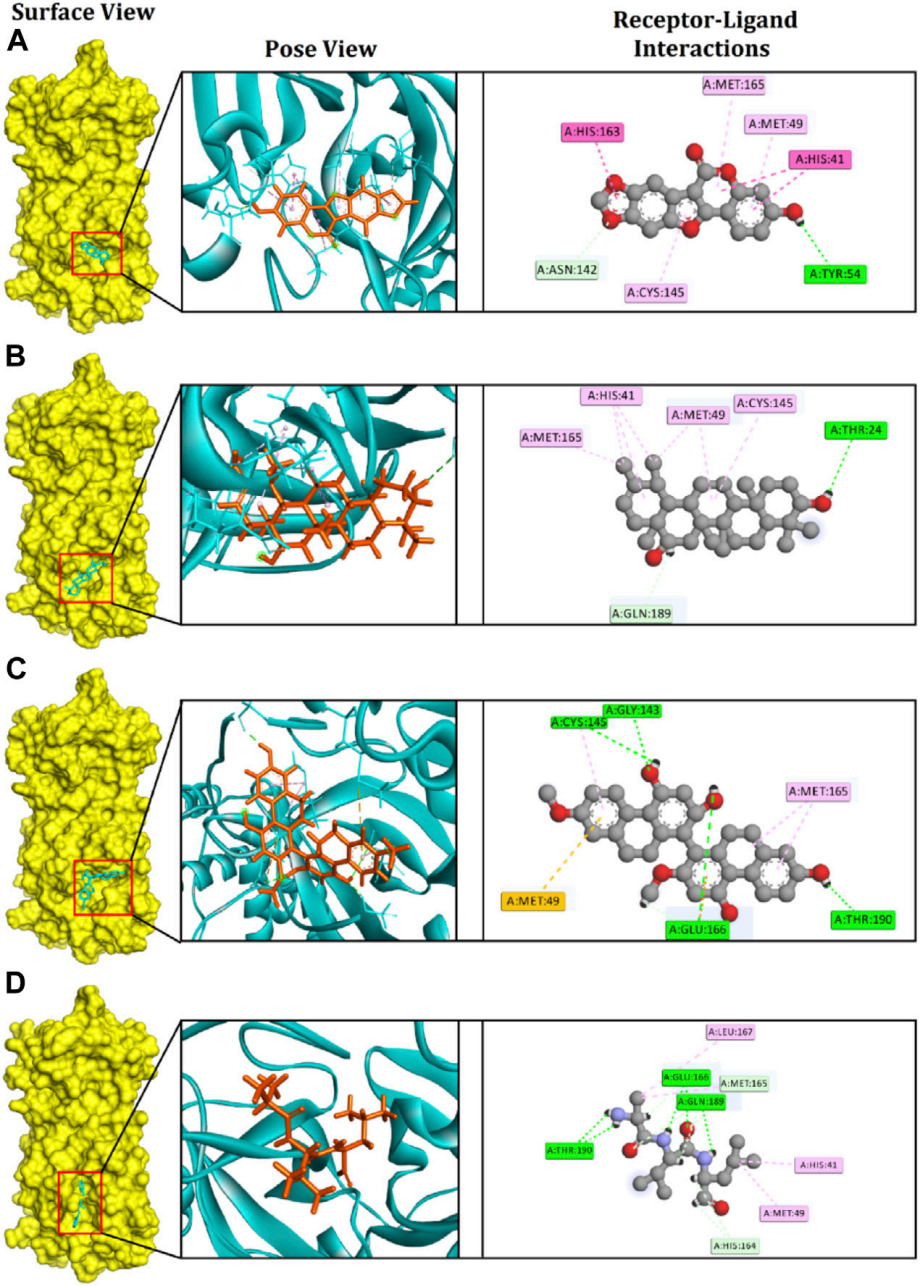
The figure illustrates non-bonded interactions of the docked complexes for top three compounds within the active and catalytic sites of the main protease. **(A)** Medicagol, **(B)** Faradiol, and **(C)** Flavanthrin.

The flavanthrin–M^pro^ drug complex forms four conventional hydrogen bonds at Cys145, Glu166, Thr190, and Gly143, a pi-sulfur bond at Met49, and an alkyl bond at Met165 **(** Table 1 and Figure 2).

The control complexes had five hydrogen bonds at Glu166, Thr190, Gln189, Met165, and His164. Also two alkyl bonds at Met49, Leu167 and one pi-alkyl interactions at His41 were also observed. The top three phytochemical compounds also exhibit similar binding interactions while docked with the M^pro^.

### ADME/T

Evaluating the toxicity and pharmacokinetics properties is necessary to assess the efficiency and indemnity level of lead molecules. Several parameters of the lead molecules, including carcinogenicity, central nervous system (CNS) permeability, p-glycoprotein inhibition, hepatotoxicity, and CYP inhibition, were examined **(** Table 2). CNS permeability indicates the capability of a compound to penetrate the semipermeable blood–brain barrier, which is designed to protect the CNS from potentially harmful substances. CNS permeability greater than −2 is considered to indicate the ability to permeate the blood–brain barrier. No toxic or carcinogenic profiles were observed for the three principal compounds. Medicagol, faradiol, and flavanthrin had molecular weights of 296.23, 442.7, and 482.5 g/mol, respectively, and aligned well with the Lipinski rule of five. Medicagol, faradiol, and flavanthrin displayed 6, 2, and 2 hydrogen bond accepters, respectively, and 1, 2, and 4 hydrogen bond donors.

**TABLE 2:** Pharmacological profiles of the top three potential candidates derived from the SwissADME, admetSAR, and pKCSM webservers.

Parameter	Medicagol	Faradiol	Flavanthrin
Molecular weight	296.23 g/mol	442.7 g/mol	482.5 g/mol
H-Bond Acceptor	6	2	6
H-Bond Donor	1	2	4
CNS	−2.064	−2.452	−2.901
CYP2D6 substrate	No	No	No
CYP3A4 substrate	No	Yes	No
CYP1A2 inhibitor	Yes	No	No
CYP2C19 inhibitor	No	Yes	No
CYP2C9 inhibitor	No	No	Yes
CYP2D6 inhibitor	No	No	No
CYP3A4 inhibitor	Yes	No	No
Carcinogenicity	Non-carcinogenic	Non-carcinogenic	Non-carcinogenic
Hepatotoxicity	No	No	No
P-glycoprotein inhibitor	No	No	No
Acute Oral Toxicity	No	No	No
Lipinski rule of five	Yes	Yes	Yes

### Biological Activities of the Drug Candidates

Various potential biological activities of the compounds were examined, including ion channel inhibition, protease inhibition, kinase inhibition, enzyme inhibition, G protein-coupled receptor (GPCR) ligand activity, and nuclear receptor ligand activity. Faradiol demonstrated the highest GPCR ligand activity, medicagol displayed the lowest GPCR ligand activity, and flavanthrin demonstrated a better ligand activity than medicagol (Table 3). The best ion channel inhibitor activity was demonstrated by faradiol, followed by flavanthrin and medicagol. However, flavanthrin exhibited better kinase inhibitor activity than both medicagol and faradiol. All screened compounds demonstrated nuclear receptor ligand activity, with that of faradiol being better than those of the other two compounds. Faradiol also exhibited better protease inhibitor activity than the other compounds.

**TABLE 3:** Biological activities of the screened hit phytochemicals were calculated from the Molinospiration chemoinformatics software package. Here Bioactivity score>0 (biologically active); −5.0 < Bioactivity score <0 (moderately active); Bioactivity score <0 (biologically inactive).

Compounds	GPCR ligand	Ion channel inhibitor	Kinase inhibitor	Nuclear receptor ligand	Protease inhibitor	Enzyme inhibitor
Medicagol	−0.30	−0.22	−0.20	0.17	−0.31	0.02
Faradiol	0.19	0.11	−0.25	0.67	0.11	0.55
Flavanthrin	0.12	0.04	0.10	0.27	−0.05	0.15

### The Molecular Dynamics Simulation Study

**FIGURE 3:**
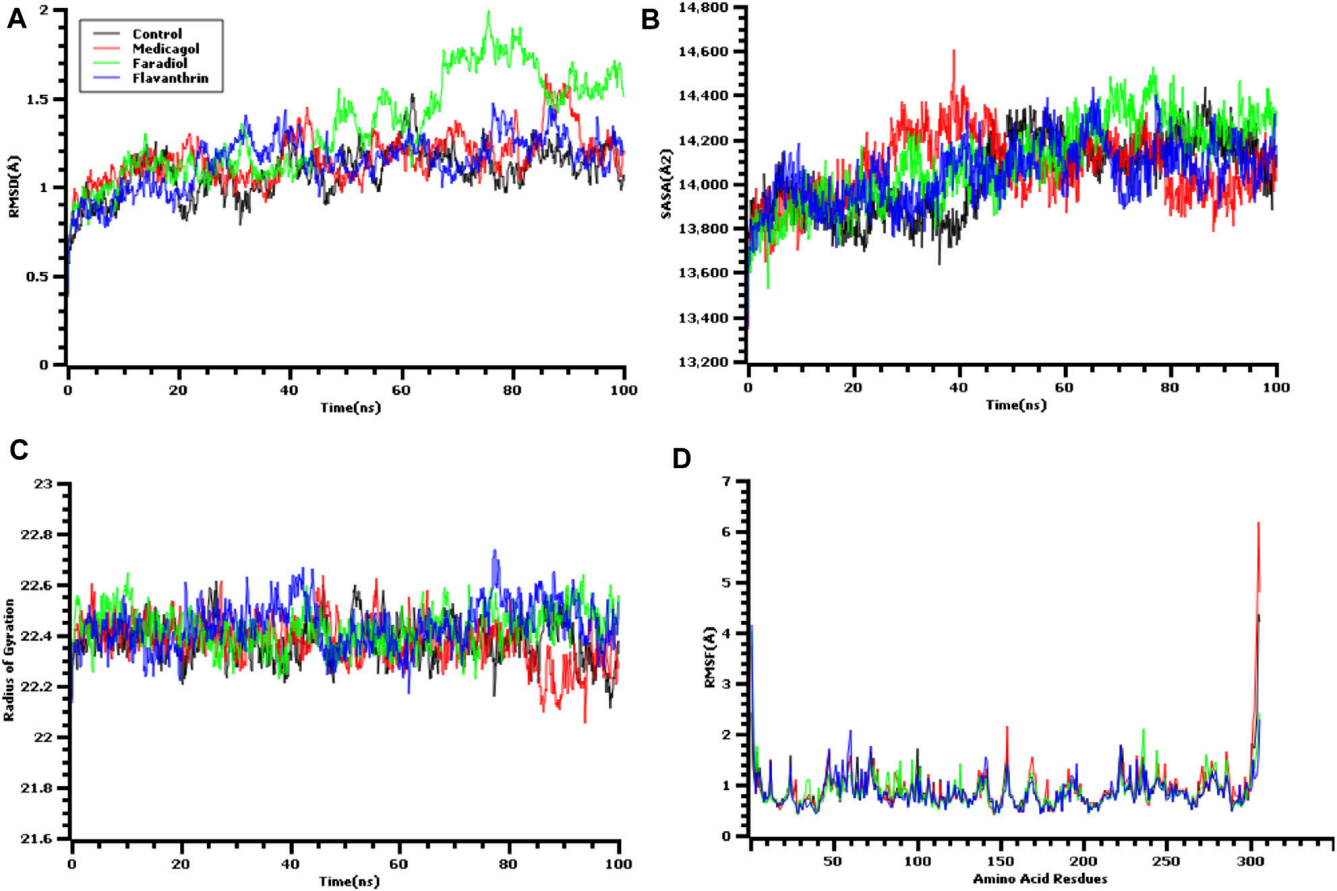
Time series analysis of all simulated systems. Panels from **(A)** to **(D)** indicate the RMSD analysis of alpha carbon atoms **(A)**, protein volume with expansion analysis **(B)**, degree of rigidity and compactness analysis **(C)**, and flexibility analysis of amino acid residues **(D)**.

A molecular dynamics simulation study was conducted, in which multiple descriptors from the simulation trajectories were analyzed to study the changes in the binding interactions and rigidity. The RMSD from the C-alpha atoms was analyzed, which revealed that all three docked complexes and the control had RMSD values below 2.5 Å throughout the entire simulation period Figure 3A. The complexes formed between M^pro^ and each of medicagol, faradiol, and flavanthrin reached an initial steady-state at the very beginning of the simulation, and the complexes containing both medicagol and flavanthrin maintained a stable profile throughout the entire simulation period. The faradiol complex had a similar profile as that for the two-protein complex for a 70-ns simulation time; however, this complex had a slightly higher RMSD profile due to increased instability.

The SASA values of the docked complexes and the control were analyzed to understand changes in the protein volume Figure 3B A higher SASA value indicates the enlargement of the protein surface area, whereas a low SASA value correlates with the minimization of the protein volume. The SASA values for the three top screened complexes initially increased during the first 30 ns of the simulation due to the extension of these complexes. After 30 ns, the complexes reached a steady-state, maintaining stability throughout the entire simulation period.

The Rg profile was assessed to determine the labile nature of the top three complexes Figure 3C. A higher Rg profile correlates with increased flexibility due to the folding or unfolding mechanism of the protein. All three complexes and the control displayed stable Rg characteristics for the docked complexes, although the Rg of the medicagol complex decreased in the 80–100 ns time window.

The RMSF values of the protein-ligand complexes were determined to assess the flexibility of the docked complexes across the amino acid residues Figure 3D. The maximum residues, except Ser1 (helix-strand), Gly2 (helix-strand), Asn72 (helix-strand), Leu232 (helix-strand), Lys236 (helix-strand), Gln244 (helix-strand), Ser301 (beta-turn), Gly302 (beta-turn), Val303 (beta-turn), Thr304 (beta-turn), Phe305 (beta-turn), and Gln306 (beta-turn), had lower RMSF profile, indicating a low degree of fluctuation.

**FIGURE 4:**
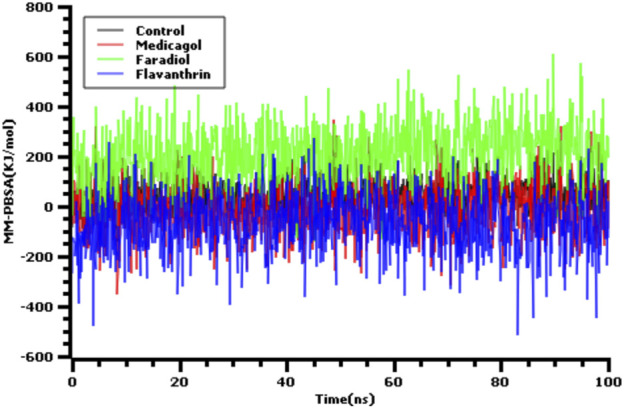
The binding free energy of the control and top three phytochemical compounds where more positive score indicates more better bindings.

Also we have calculated the binding free energy via MM-PBSA methods where the more positive energy indicates better bindings. The average binding free energy of the control, medicagol, faradiol, and flavanthrin were 43.33, −12.12, 210.78, −68.67 kJ/mol respectively (Figure 4). The faradiol had more binding free energy which indicates the comparative favorable binding of this ligand molecule. The other complexes had similar free energy compared to the control molecules which indicates better binding with these ligand molecules.

**FIGURE 5:**
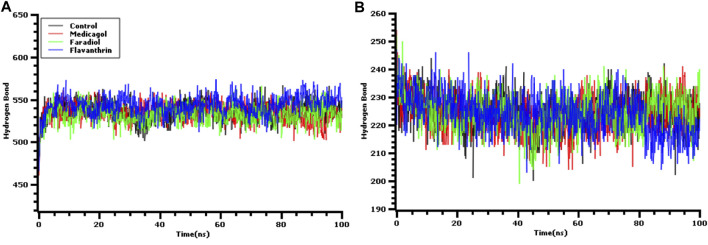
The hydrogen bond analysis from the simulation trajectories where every snapshot were taken into consideration for the graph generations. The **(** **A** **)** hydrogen bond between solute and the solvents, and **(** **B** **)** the hydrogen bond in the solute.

The hydrogen bonds in the simulation system were precisely verified to evaluate the stable nature of the three hit candidates, as any deviations in hydrogen bond patterns and numbers can increase flexibility Figures 5A,B. The medicagol, faradiol, flavanthrin, and control complexes all displayed low levels of deviation and maintained integrity throughout the entire simulation trajectory. The flavanthrin complex displayed more hydrogen bonds in the simulation snapshots than either complex formed by medicagol or faradiol, which indicates the increased rigidity of the complex containing flavanthrin.

The binding interactions were assessed after 100-ns simulation studies to explore any deviations in the binding interactions for the docked complexes. Medicagol formed two hydrogen bonds with the SARS-CoV-2 M^pro^ at His41and Glu166 and one pi-pi-T-shaped interaction at Met165 (Table 4). The faradiol complex formed two hydrogen bonds at Met49 and Met165 and two pi-alkyl interactions at Cys145 and His41. The flavanthrin and M^pro^ complex created the highest number of non-bonded interactions compared with the other two complexes, including four hydrogen bonds at Thr190, Asn142, GLy143, and Gln192, two pi-sigma bonds at Gln189 and Met165, one amide-pi-stacked bond at Gln189, and two pi-alkyl bonds at Pro168 and His41.

**TABLE 4:** The non-bonded interactions of the docked complexes after a 100 ns simulation time; here, H, PPT, A, PA, PS, and APS refer to hydrogen, pi-pi-T-shaped, alkyl, pi-alkyl, pi-sigma, amide pi-stacked bonds, respectively.

Complex	Residues	Interaction Type	Distance(Å)
Medicagol	His41	H	2.29
	Glu166	H	3.16
	Met165	PPT	5.28
Faradiol	Met49	H	2.31
	Met165	H	2.23
	Cys145	PA	3.48
	His41	PA	4.59
Flavanthrin	Thr190	H	1.88
	Asn142	H	2.55
	Gly143	H	2.08
	Gln192	H	2.01
	Gln189	PS	2.42
	Met165	PS	5.18
	Gln189	APS	4.12
	Pro168	PA	4.86
	His41	PA	5.33
	Glu166	H	2.04
	Gln189	H	2.71
	Thr190	H	2.00
	Met165	H	2.63
	Met49	A	4.69
	Leu167	A	5.30
	HIS41	PA	4.10

## Discussion

A novel etiological agent has been identified that induces a viral, pneumonia-like disease, labeled SARS-CoV-2, and the declaration of a global pandemic has disrupted both healthcare and economic systems worldwide (McKee et al., 2020; Ren et al., 2020). The extremely contagious and potentially deadly SARS-CoV-2 virus is transmitted through aerosolized droplets or fomite and has resulted in widespread fatalities on a global scale. To fight the spread of SARS-CoV-2, diverse treatments have advanced to clinical trials, several of which are ongoing, but the development of new therapies has been impeded by high costs and the time-consuming nature of basic science research. However, computer-aided molecular drug design schemes can rapidly and accurately be used to screen potentially active drugs from among large small-molecule libraries to identify novel molecules with the potential to counteract the effects of SARS-CoV-2.

The SARS-CoV-2 M^pro^ serves several essential functions in viral propagation that make this protein an excellent drug target. Two polyproteins, pp1a and pp1ab, are translated from the SARS-CoV-2 viral RNA, and M^pro^ is essential for processing these polyproteins into their active protein components. Therefore, M^pro^ plays crucial roles in both viral propagation viral genome replication. The development of an M^pro^ inhibitor could obstruct viral amplification (Mathpal et al., 2020; Tripathi et al., 2020; Mahmud et al., 2021d). The cysteine protease-based M^pro^ contains a catalytic dyad in the active center. M^pro^ is comprised of three domains: Domain, I consisting of amino acid residue 1–99; Domain II, consisting of amino acids 100–182; and Domain III, consisting of amino acid residues 198–303 (Ullrich and Nitsche, 2020; Mahmud et al., 2021d). The catalytic residues Cys145 and His61 are activated through dimerization, representing a potential SARS-CoV-2 M^pro^ inhibition mechanism to target for drug development. The catalytic site contains two shallow subsites, labeled S1 and S2, in addition to three additional shallow subsites known as S3, S4, and S5. The S1 shallow subsite is formed by the Phe140, His163, Glu166, His172, and Gly143 residues, whereas Thr25, His41, and Cys145 comprise the S2 subsite. Five residues, Glu166, His41, Met165, Gln189, and Met45, comprise the S3, S4, and S5 shallow subsite (Khan et al., 2020; Dai et al., 2020; Kalhor et al., 2020; Mahmud et al., 2021d).

During the process of identifying and developing potential drug candidates among known phytochemicals using experimental and computational approaches, molecular docking can provide crucial information through the prediction of probable binding modes, allowing the rapid screening of multiple molecules. Molecular docking analysis can identify ligand binding sites with substantial accuracy and provide quantitative predictions of free ligand-receptor energy allowing ligands to be ranked according to likely binding abilities during computational drug design. Virtual ligand screens can be applied to large collections of potentially active molecules, and the docking system can be used to rapidly identify those with a strong binding affinity that may be developed into drug candidates (F. Sousa et al., 2010; Ferreira et al., 2015; Macalino et al., 2015). Computational approaches are cost-effective methods that can circumvent time-consuming real-world screening processes, and binding affinity predictions can be used to reduce the massive array of diverse phytochemicals to a few highly probable drug candidates. In this manuscript, our best three targeted candidates had binding energy values of −8.3, −8.6, and −8.8 kcal/mol, which is highly significant because, in molecular docking approaches, lower binding energy indicates higher binding affinity, and also exhibited better energy than the control systems. Compounds with a high binding affinity for target proteins are frequently considered to have strong potential as effective inhibitors that can efficiently impede protein activity (Hsu et al., 2008; Parenti and Rastelli, 2012; Joshi et al., 2020).

Based on the estimated binding affinities, the three best phytochemicals were selected, and the predicted binding residues were analyzed. The first phytochemical compound, medicagol, demonstrated anti-collagenase, anti-elastase, and antioxidant properties in enzymatic assays (Thring et al., 2009). Medicagol formed numerous non-covalent interactions with the active groove of M^pro^ at His41, Cys145, Met165, and Met49, which are key amino acids for targeted inhibition. After a 100-ns simulation, interactions with active sites of SARS-CoV-2 M^pro^ (His41 and Met165) were identified. Faradiol demonstrated numerous activities under lab conditions, including anti-inflammatory activity (Colombo et al., 2015), inhibitory effects against tumor promotion (Yasukawa et al., 1996), and anti-edematous activity (Zitterl-Eglseer et al., 1997). Faradiol formed multiple interactions with active sites of M^pro^, including Gln189, Met49, Met165, Cys145, and His41, and the binding rigidity with these residues remained constant. Flavanthrin displayed cytotoxic effects under wet lab conditions (Chang et al., 2015), and this compound also formed multiple interactions at the active sites of the SARS-CoV-2 M^pro^, including Cys145, Glu166, Thr190, and Met49. Ligand binding to the catalytic sites of the protein may play a key role in targeted inhibition (Mahmud et al., 2021d).

**FIGURE 6:**
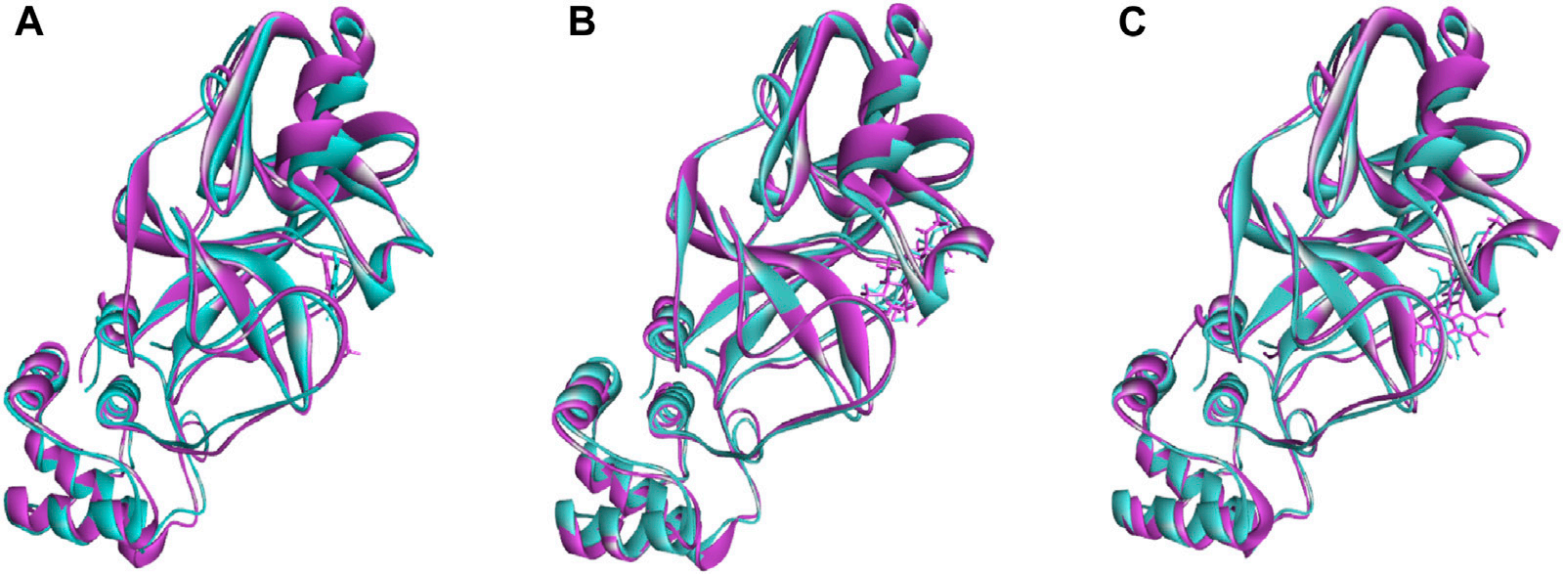
The superimposition between pre- and post-molecular dynamics structures, where lower root-mean-square deviations were found. The sky color indicates the pre-molecular dynamics structure, and the pink color indicates the post-molecular dynamics structure.

**FIGURE 7:**
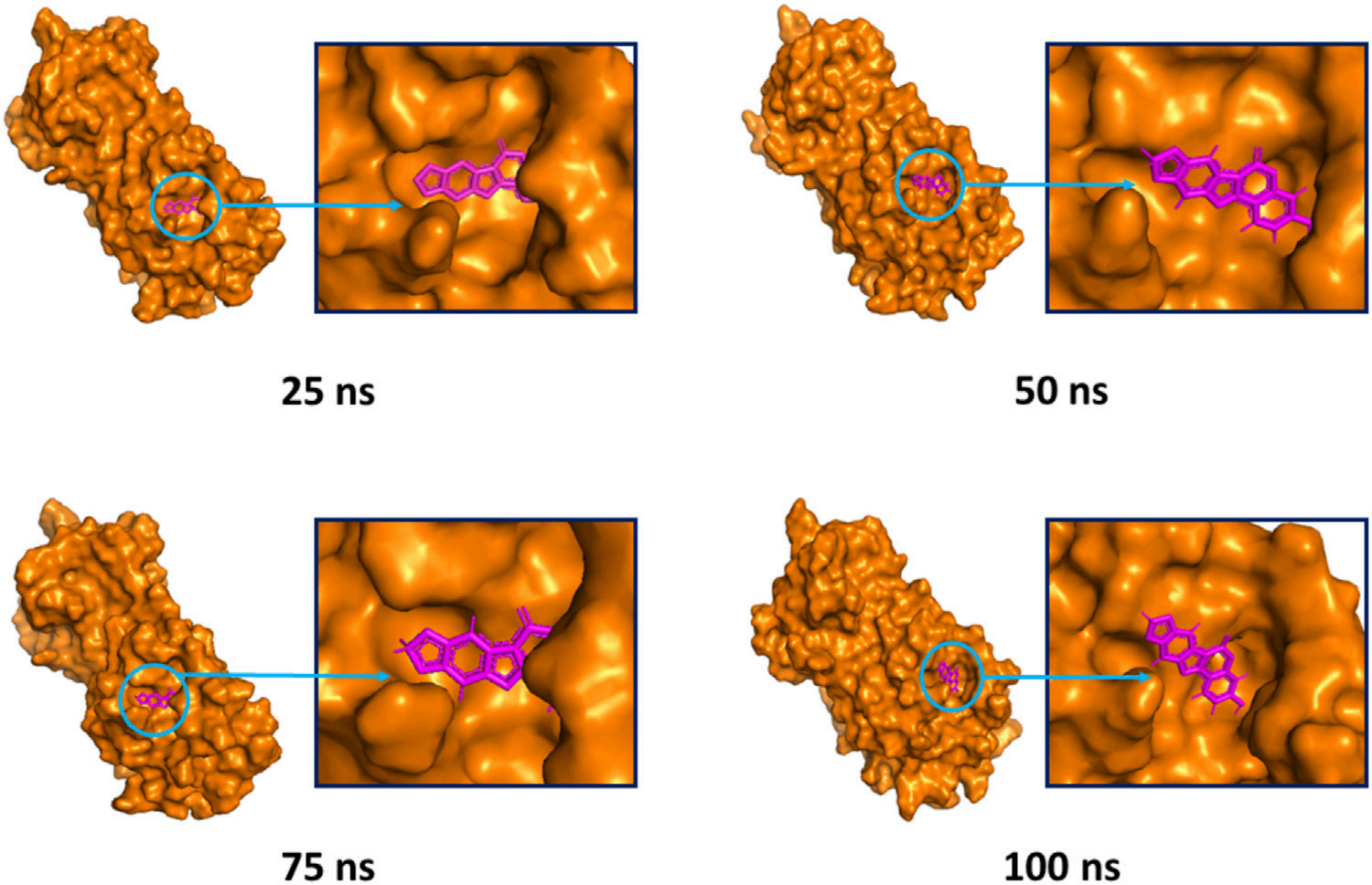
The surface view of the docked complex during the molecular dynamics simulation. Snapshots were taken at 25, 50, 75, and 100 ns for the medicagol and M^pro^ complex. The binding pose and positions of the ligands were remained rigid in different simulation time intervals.

**FIGURE 8:**
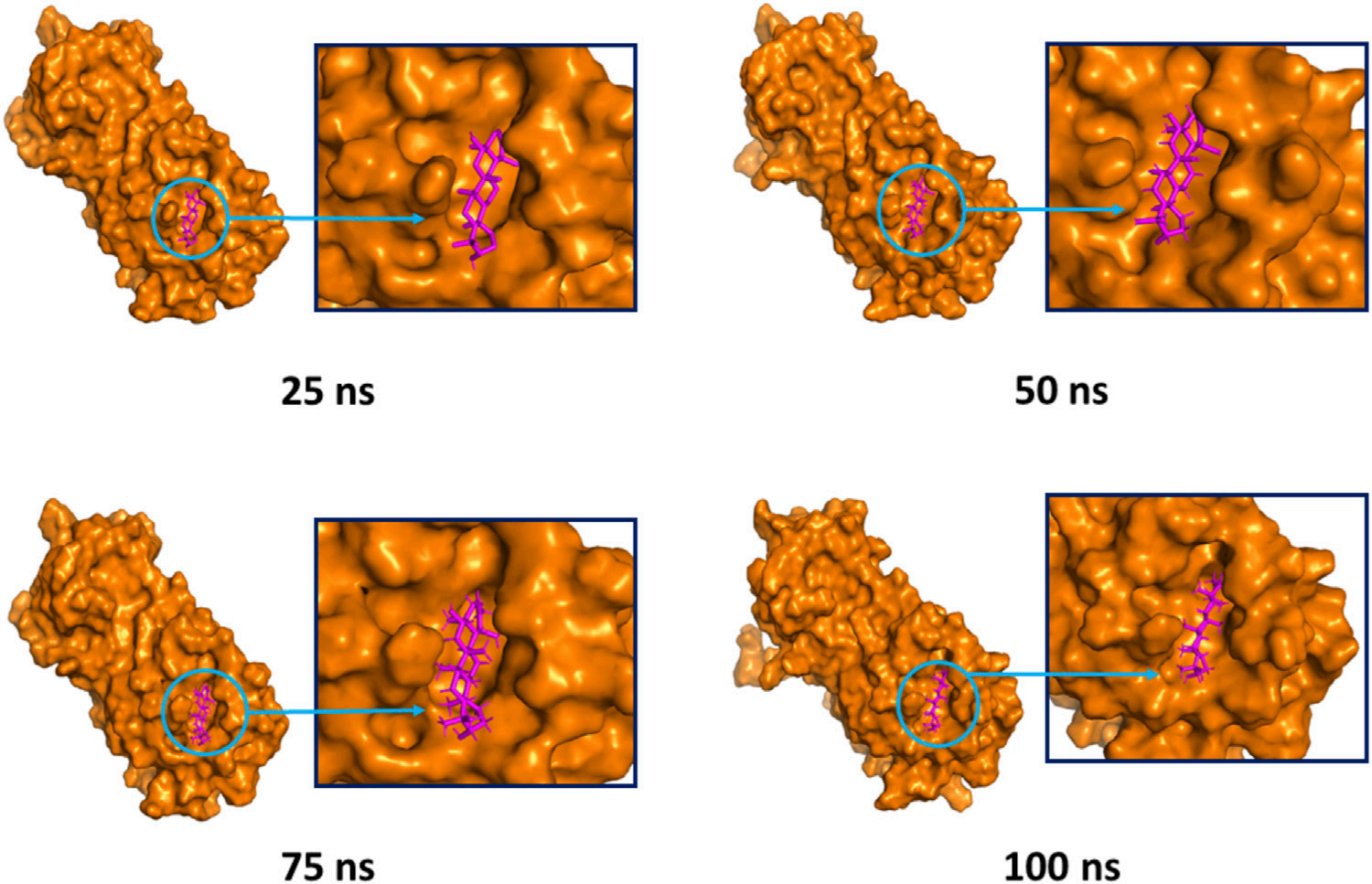
The surface view and the binding pockets of the faradiol and M^pro^ complex, for which the 25, 50, 75, and 100 ns snapshots were taken. The ligand molecules and binding in the interacting pockets of the proteins were remained similar across different simulation time intervals.

**FIGURE 9:**
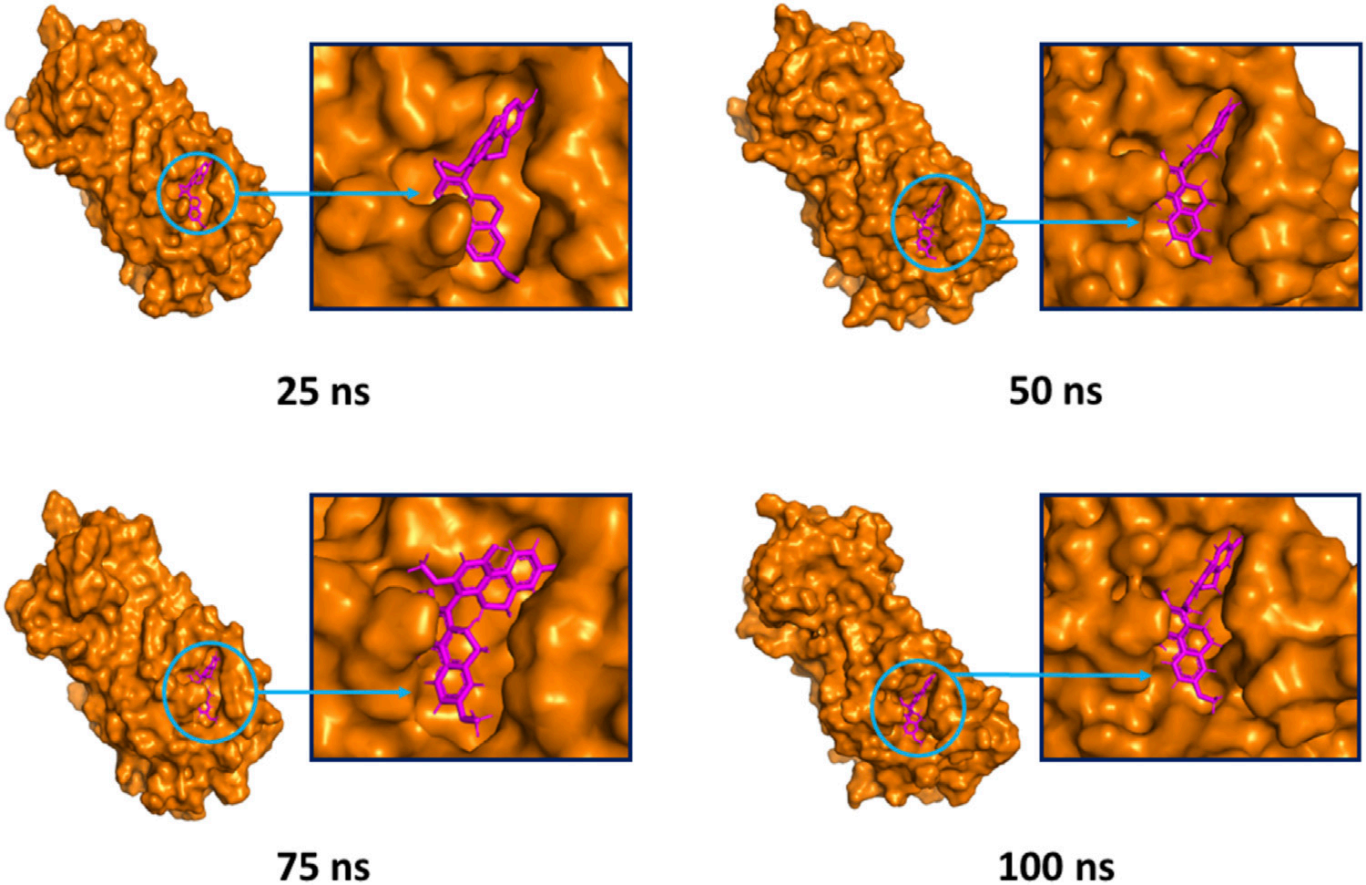
The surface view of the docked flavanthrin and M^pro^ complex, shown as 25, 50, 75, and 100 ns snapshots. The binding pose and interactions were remained in the same binding pockets. The figure were generated from Pymol software package.

The superimposition between pre- and post-molecular dynamics structures was performed to identify differences in the docked complexes. The three docked complexes had low deviations in their structures, and the medicagol, faradiol, and flavanthrin complexes with M^pro^ had RMSD values of 1.33, 1.50, and 1.20 Å, respectively, which indicated a low degree of changes after the 100-ns simulation (Figure 6). The simulated trajectories were also analyzed after 25, 50, 75, and 100 ns to identify any alterations in the binding pockets. Figures 7–9 indicate that the top three screened ligand molecules had similar binding poses and rigidity over all examined simulation trajectories when bound to M^pro^. The interacting residues and the binding pose of the ligand and protein complexes had lower aberrations in binding pockets as they stayed in the similar orientation across the simulation times. These results further supports the superimpositions of the Pre and Post MD structures in Figure 4.

The combination of bioinformatics approaches, including molecular docking and molecular dynamics studies, suggested that the three screened phytochemicals may have the ability to interfere with the function of the SARS-CoV-2 M^pro^. Also the comparison with the co-crystalized ligand molecules and the top three screened molecules provides better insights about M^pro^ targeted inhibitions. Furthermore the development of a new phytochemical datasets will allow fellow researchers to work against other targeted viral protein or signaling molecules. Although this study was validated in multiple computational algorithms but these data need to be validated at the wet lab conditions and in different enzymatic assays.

## Conclusion

This study utilized a structure-based drug design process to screen phytochemicals with potent inhibitory function against the SARS-CoV-2 M^pro^. We screened thousands of phytochemicals identified in various Asian plants and assessed their binding affinities for M^pro^ using a molecular docking program. The three (medicagol, faradiol, and flavanthrin) best compounds were selected, which were demonstrated to multiple, non-covalent interactions at the active region of SARS-CoV-2 M^pro^. Furthermore, the molecular dynamics study validated the binding poses and structural stabilities of the docked complexes by exploring multiple parameters from the simulation trajectories. The toxicity and carcinogenicity of the screened molecules indicated positive drug-likeness properties, which are crucial to ensuring drug safety. This study depended exclusively on computational pipelines; therefore, additional evaluations remain necessary to test these compounds under wet lab conditions. However, these computational approaches may aid researchers in the identification of precise compounds that may function as SARS-CoV-2 M^pro^ inhibitors.

## Data Availability

The original contributions presented in the study are included in the article/Supplementary Material, further inquiries can be directed to the corresponding authors.
