# Supplementary material for: Screening of Potent Phytochemical Inhibitors Against SARS-CoV-2 Main Protease: An Integrative Computational Approach
Source: Front Bioinform. 2021 Oct 5;1:717141. doi: 10.3389/fbinf.2021.717141 (PMC9581031; doi:10.3389/fbinf.2021.717141)
Supplement: Supplementary file 1 [file DataSheet2.PDF]

TABLE S3 The binding energy of the all compounds generated from AutoDock Vina.

Ligand	Binding Affinity	rmsd/ub	rmsd/lb
6lu7_EM_72_uff_E=70.72	-5.8	0	0
6lu7_EM_126_uff_E=57.33	-5.1	0	0
6lu7_EM_127_uff_E=89.11	-5.8	0	0
6lu7_EM_135_uff_E=62.95	-5.8	0	0
6lu7_EM_240_uff_E=52.88	-5	0	0
6lu7_EM_243_uff_E=59.30	-5.9	0	0
6lu7_EM_247_uff_E=88.22	-3.8	0	0
6lu7_EM_289_uff_E=55.42	-5.2	0	0
6lu7_EM_305_uff_E=103.14	-3.3	0	0
6lu7_EM_332_uff_E=99.78	-5.3	0	0
6lu7_EM_338_uff_E=73.50	-6.1	0	0
6lu7_EM_370_uff_E=77.82	-5.7	0	0
6lu7_EM_379_uff_E=30.18	-4.9	0	0
6lu7_EM_454_uff_E=27.41	-4.2	0	0
6lu7_EM_595_uff_E=124.04	-4.9	0	0
6lu7_EM_750_uff_E=27.51	-3.8	0	0
6lu7_EM_785_uff_E=52.91	-4.7	0	0
6lu7_EM_932_uff_E=195.80	-6.9	0	0
6lu7_EM_936_uff_E=74.86	-5.2	0	0
6lu7_EM_957_uff_E=39.46	-4.3	0	0
6lu7_EM_985_uff_E=57.46	-5.1	0	0
6lu7_EM_995_uff_E=161.17	-6.1	0	0
6lu7_EM_1001_uff_E=91.78	-5.2	0	0
6lu7_EM_1017_uff_E=151.39	-6	0	0
6lu7_EM_1110_uff_E=26.16	-4.7	0	0
6lu7_EM_1174_uff_E=44.82	-5.2	0	0
6lu7_EM_1183_uff_E=79.78	-5.1	0	0
6lu7_EM_2214_uff_E=155.34	-5.4	0	0
6lu7_EM_2346_uff_E=79.77	-4.7	0	0
6lu7_EM_2353_uff_E=579.86	-5.4	0	0
6lu7_EM_2518_uff_E=98.61	-6	0	0
6lu7_EM_2950_uff_E=185.78	-7.1	0	0
6lu7_EM_2969_uff_E=38.20	-5	0	0
6lu7_EM_3026_uff_E=212.61	-5.4	0	0
6lu7_EM_3220_uff_E=196.51	-7.3	0	0
6lu7_EM_3314_uff_E=169.59	-5.3	0	0
6lu7_EM_3469_uff_E=78.01	-6	0	0
6lu7_EM_3893_uff_E=43.33	-5.2	0	0
6lu7_EM_4133_uff_E=86.76	-5.4	0	0
6lu7_EM_4735_uff_E=245.94	-6.5	0	0
6lu7_EM_5417_uff_E=594.17	-6.6	0	0
6lu7_EM_5574_uff_E=318.49	-5.1	0	0
6lu7_EM_5634_uff_E=57.21	-5.2	0	0
6lu7_EM_5770_uff_E=859.40	-6.4	0	0
6lu7_EM_5793_uff_E=184.96	-4.6	0	0
6lu7_EM_5950_uff_E=33.09	-3.9	0	0
6lu7_EM_5951_uff_E=74.76	-4.1	0	0
6lu7_EM_5960_uff_E=39.14	-4.5	0	0
6lu7_EM_5962_uff_E=63.08	-4.6	0	0
6lu7_EM_5988_uff_E=487.38	-5.3	0	0
6lu7_EM_5997_uff_E=549.32	-7.4	0	0
6lu7_EM_6029_uff_E=306.70	-6.2	0	0
6lu7_EM_6036_uff_E=190.36	-5.3	0	0
6lu7_EM_6043_uff_E=312.52	-5.4	0	0
6lu7_EM_6057_uff_E=109.06	-5.8	0	0
6lu7_EM_6106_uff_E=72.66	-4.7	0	0
6lu7_EM_6137_uff_E=94.18	-4	0	0
6lu7_EM_6140_uff_E=104.20	-5.6	0	0
6lu7_EM_6251_uff_E=158.46	-4.8	0	0
6lu7_EM_6255_uff_E=385.08	-6	0	0
6lu7_EM_6274_uff_E=256.87	-5	0	0
6lu7_EM_6287_uff_E=55.97	-4.2	0	0
6lu7_EM_6288_uff_E=64.57	-4.7	0	0
6lu7_EM_6293_uff_E=182.03	-7.1	0	0

6lu7_EM_6305_uff_E=333.02	-6	0	0
6lu7_EM_6306_uff_E=69.94	-4.3	0	0
6lu7_EM_6322_uff_E=70.50	-4.8	0	0
6lu7_EM_6508_uff_E=156.41	-5.7	0	0
6lu7_EM_6654_uff_E=633.64	-5.1	0	0
6lu7_EM_6683_uff_E=197.40	-7.2	0	0
6lu7_EM_6780_uff_E=133.80	-6.9	0	0
6lu7_EM_6782_uff_E=255.99	-5.7	0	0
6lu7_EM_6919_uff_E=208.82	-4.9	0	0
6lu7_EM_7057_uff_E=95.90	-6.5	0	0
6lu7_EM_7150_uff_E=68.70	-5.1	0	0
6lu7_EM_7302_uff_E=151.57	-3.8	0	0
6lu7_EM_7427_uff_E=388.09	-5.9	0	0
6lu7_EM_7428_uff_E=94.73	-5.7	0	0
6lu7_EM_7439_uff_E=108.57	-5	0	0
6lu7_EM_7460_uff_E=154.38	-5.1	0	0
6lu7_EM_7461_uff_E=63.24	-5.3	0	0
6lu7_EM_7462_uff_E=122.04	-4.9	0	0
6lu7_EM_7463_uff_E=91.91	-5.6	0	0
6lu7_EM_7501_uff_E=71.29	-4.9	0	0
6lu7_EM_7695_uff_E=106.64	-5.1	0	0
6lu7_EM_7800_uff_E=53.04	-4.3	0	0
6lu7_EM_7824_uff_E=33.29	-3.7	0	0
6lu7_EM_7972_uff_E=103.11	-3.2	0	0
6lu7_EM_8029_uff_E=199.87	-3.3	0	0
6lu7_EM_8042_uff_E=83.23	-5	0	0
6lu7_EM_8049_uff_E=65.24	-4.1	0	0
6lu7_EM_8078_uff_E=30.11	-3.6	0	0
6lu7_EM_8129_uff_E=36.31	-4.3	0	0
6lu7_EM_8130_uff_E=24.07	-3.8	0	0
6lu7_EM_8138_uff_E=49.33	-4.2	0	0
6lu7_EM_8141_uff_E=23.72	-3.6	0	0
6lu7_EM_8143_uff_E=37.18	-4	0	0
6lu7_EM_8158_uff_E=33.81	-4.6	0	0
6lu7_EM_8164_uff_E=33.74	-4.1	0	0
6lu7_EM_8176_uff_E=33.04	-4	0	0
6lu7_EM_8180_uff_E=40.64	-4.7	0	0
6lu7_EM_8181_uff_E=66.14	-4.2	0	0
6lu7_EM_8194_uff_E=41.12	-4.2	0	0
6lu7_EM_8369_uff_E=63.99	-4.4	0	0
6lu7_EM_8468_uff_E=150.75	-5.5	0	0
6lu7_EM_8575_uff_E=210.98	-5.9	0	0
6lu7_EM_8655_uff_E=168.50	-4.8	0	0
6lu7_EM_8785_uff_E=85.97	-5.6	0	0
6lu7_EM_8794_uff_E=234.72	-4.5	0	0
6lu7_EM_8830_uff_E=60.42	-4.2	0	0
6lu7_EM_8892_uff_E=24.06	-4.7	0	0
6lu7_EM_8969_uff_E=497.11	-7.3	0	0
6lu7_EM_9064_uff_E=204.84	-7.4	0	0
6lu7_EM_9121_uff_E=435.87	-7.2	0	0
6lu7_EM_9210_uff_E=86.84	-5	0	0
6lu7_EM_9294_uff_E=150.09	-5	0	0
6lu7_EM_9415_uff_E=463.48	-6.6	0	0
6lu7_EM_9895_uff_E=172.51	-5.4	0	0
6lu7_EM_10143_uff_E=597.01	-7.2	0	0
6lu7_EM_10151_uff_E=237.56	-7.1	0	0
6lu7_EM_10168_uff_E=201.65	-7.3	0	0
6lu7_EM_10177_uff_E=620.08	-7.4	0	0
6lu7_EM_10206_uff_E=1064.03	-7.2	0	0
6lu7_EM_10207_uff_E=212.95	-7.4	0	0
6lu7_EM_10208_uff_E=215.65	-7.4	0	0
6lu7_EM_10215_uff_E=571.00	-6.9	0	0
6lu7_EM_10231_uff_E=304.43	-5.1	0	0
6lu7_EM_10235_uff_E=197.51	-5.7	0	0
6lu7_EM_10349_uff_E=28.89	-4.7	0	0
6lu7_EM_10393_uff_E=97.81	-5.6	0	0

6lu7_EM_10408_uff_E=120.66	-4.6	0	0
6lu7_EM_10416_uff_E=2180.77	-4.8	0	0
6lu7_EM_10465_uff_E=60.66	-4.8	0	0
6lu7_EM_10494_uff_E=689.79	-6.9	0	0
6lu7_EM_10582_uff_E=649.92	-5.3	0	0
6lu7_EM_10639_uff_E=212.65	-7.3	0	0
6lu7_EM_10703_uff_E=80.24	-5.2	0	0
6lu7_EM_10742_uff_E=110.08	-5.7	0	0
6lu7_EM_10976_uff_E=41.95	-4.4	0	0
6lu7_EM_11005_uff_E=51.08	-4.6	0	0
6lu7_EM_11006_uff_E=47.83	-3.8	0	0
6lu7_EM_11066_uff_E=599.93	-7.2	0	0
6lu7_EM_11142_uff_E=124.80	-5.4	0	0
6lu7_EM_11230_uff_E=155.64	-4.8	0	0
6lu7_EM_11468_uff_E=134.28	-5.7	0	0
6lu7_EM_11503_uff_E=100.47	-4.8	0	0
6lu7_EM_11605_uff_E=117.50	-3.9	0	0
6lu7_EM_11915_uff_E=102.80	-6.4	0	0
6lu7_EM_12398_uff_E=50.10	-3.7	0	0
6lu7_EM_12523_uff_E=140.35	-4.3	0	0
6lu7_EM_12530_uff_E=46.39	-4.4	0	0
6lu7_EM_12921_uff_E=2186.45	-4.4	0	0
6lu7_EM_13229_uff_E=89.44	-5.5	0	0
6lu7_EM_13250_uff_E=88.97	-5.7	0	0
6lu7_EM_13849_uff_E=53.38	-4.2	0	0
6lu7_EM_14257_uff_E=30.71	-4	0	0
6lu7_EM_14896_uff_E=713.31	-5.1	0	0
6lu7_EM_15611_uff_E=58.50	-4.7	0	0
6lu7_EM_15612_uff_E=60.32	-4.1	0	0
6lu7_EM_15979_uff_E=127.95	-4.4	0	0
6lu7_EM_16217_uff_E=55.80	-4.3	0	0
6lu7_EM_16331_uff_E=42.40	-4.2	0	0
6lu7_EM_16913_uff_E=506.65	-5.3	0	0
6lu7_EM_17868_uff_E=1384.84	-4.8	0	0
6lu7_EM_18721_uff_E=459.52	-7	0	0
6lu7_EM_18950_uff_E=195.52	-5	0	0
6lu7_EM_19009_uff_E=523.49	-6.7	0	0
6lu7_EM_19212_uff_E=710.49	-7.2	0	0
6lu7_EM_19725_uff_E=770.21	-5.9	0	0
6lu7_EM_21057_uff_E=42.23	-3.9	0	0
6lu7_EM_21205_uff_E=77.38	-4.4	0	0
6lu7_EM_22311_uff_E=102.50	-5.2	0	0
6lu7_EM_22955_uff_E=436.93	-7.3	0	0
6lu7_EM_23518_uff_E=62.94	-4.7	0	0
6lu7_EM_23741_uff_E=1468.22	-3.9	0	0
6lu7_EM_23915_uff_E=955.76	-7	0	0
6lu7_EM_24197_uff_E=70.53	-4.5	0	0
6lu7_EM_25310_uff_E=179.95	-5.2	0	0
6lu7_EM_28454_uff_E=85.05	-4	0	0
6lu7_EM_28469_uff_E=168.40	-5.5	0	0
6lu7_EM_28565_uff_E=522.34	-6.7	0	0
6lu7_EM_29566_uff_E=1102.39	-6.1	0	0
6lu7_EM_31209_uff_E=261.93	-5.1	0	0
6lu7_EM_31244_uff_E=74.15	-4.7	0	0
6lu7_EM_31253_uff_E=90.99	-4.2	0	0
6lu7_EM_31268_uff_E=212.35	-3.1	0	0
6lu7_EM_31283_uff_E=83.49	-3.8	0	0
6lu7_EM_31285_uff_E=24.64	-3.9	0	0
6lu7_EM_31703_uff_E=616.59	-7	0	0
6lu7_EM_33032_uff_E=55.55	-4.8	0	0
6lu7_EM_33624_uff_E=517.75	-5.3	0	0
6lu7_EM_38762_uff_E=63.64	-4	0	0
6lu7_EM_43595_uff_E=911.77	-6.1	0	0
6lu7_EM_60961_uff_E=543.04	-6.5	0	0
6lu7_EM_60985_uff_E=117.40	-4.6	0	0
6lu7_EM_61030_uff_E=46.56	-4.4	0	0

6lu7_EM_61126_uff_E=654.84	-5.2	0	0
6lu7_EM_61303_uff_E=48.96	-4.1	0	0
6lu7_EM_62321_uff_E=132.77	-4.8	0	0
6lu7_EM_62367_uff_E=1462.27	-5	0	0
6lu7_EM_62566_uff_E=899.87	-5.7	0	0
6lu7_EM_62752_uff_E=193.41	-4.5	0	0
6lu7_EM_64945_uff_E=805.65	-7.2	0	0
6lu7_EM_64971_uff_E=793.31	-7.4	0	0
6lu7_EM_65064_uff_E=325.39	-7.3	0	0
6lu7_EM_65366_uff_E=1548.87	-6.8	0	0
6lu7_EM_65373_uff_E=444.03	-6.2	0	0
6lu7_EM_65411_uff_E=4795.40	-7.4	0	0
6lu7_EM_65575_uff_E=580.54	-6	0	0
6lu7_EM_67328_uff_E=33.86	-3.9	0	0
6lu7_EM_68071_uff_E=214.20	-7	0	0
6lu7_EM_68167_uff_E=87.11	-4.4	0	0
6lu7_EM_69867_uff_E=287.71	-6.2	0	0
6lu7_EM_70627_uff_E=83.63	-5	0	0
6lu7_EM_72276_uff_E=230.94	-7.5	0	0
6lu7_EM_72277_uff_E=237.97	-7.3	0	0
6lu7_EM_72281_uff_E=283.48	-7.4	0	0
6lu7_EM_72310_uff_E=445.58	-7.3	0	0
6lu7_EM_72323_uff_E=720.42	-7.1	0	0
6lu7_EM_72326_uff_E=948.71	-7.2	0	0
6lu7_EM_72369_uff_E=673.25	-7.3	0	0
6lu7_EM_72537_uff_E=237.73	-7.1	0	0
6lu7_EM_72610_uff_E=267.25	-5.9	0	0
6lu7_EM_73145_uff_E=694.14	-7.1	0	0
6lu7_EM_73170_uff_E=3374.53	-6.8	0	0
6lu7_EM_73193_uff_E=781.69	-6.8	0	0
6lu7_EM_73253_uff_E=479.66	-7.1	0	0
6lu7_EM_73307_uff_E=87.58	-4.8	0	0
6lu7_EM_73330_uff_E=771.59	-6.8	0	0
6lu7_EM_73337_uff_E=577.41	-6.6	0	0
6lu7_EM_73399_uff_E=626.81	-7.4	0	0
6lu7_EM_73481_uff_E=958.07	-6.7	0	0
6lu7_EM_73568_uff_E=749.04	-6.5	0	0
6lu7_EM_73571_uff_E=212.55	-7.2	0	0
6lu7_EM_73659_uff_E=837.07	-7.2	0	0
6lu7_EM_74416_uff_E=54.58	-3.9	0	0
6lu7_EM_75704_uff_E=40.47	-4.5	0	0
6lu7_EM_77466_uff_E=115.76	-4.1	0	0
6lu7_EM_77547_uff_E=94.40	-5.8	0	0
6lu7_EM_78435_uff_E=265.89	-5.4	0	0
6lu7_EM_79043_uff_E=1556.99	-4.7	0	0
6lu7_EM_81101_uff_E=64.17	-4.3	0	0
6lu7_EM_81747_uff_E=93.93	-4.9	0	0
6lu7_EM_82143_uff_E=474.62	-7.1	0	0
6lu7_EM_82755_uff_E=106.56	-5.7	0	0
6lu7_EM_86609_uff_E=1517.00	-6.4	0	0
6lu7_EM_86770_uff_E=57.18	-4.2	0	0
6lu7_EM_89047_uff_E=304.37	-5.9	0	0
6lu7_EM_91354_uff_E=1698.37	-6.3	0	0
6lu7_EM_91439_uff_E=860.89	-6.2	0	0
6lu7_EM_91457_uff_E=194.77	-5.9	0	0
6lu7_EM_91466_uff_E=243.56	-6.6	0	0
6lu7_EM_91472_uff_E=973.63	-6.8	0	0
6lu7_EM_91510_uff_E=496.67	-6.7	0	0
6lu7_EM_92123_uff_E=298.25	-6.6	0	0
6lu7_EM_92139_uff_E=113.33	-5.5	0	0
6lu7_EM_92158_uff_E=853.30	-6.6	0	0
6lu7_EM_92231_uff_E=1727.92	-6.3	0	0
6lu7_EM_92780_uff_E=122.60	-4.6	0	0
6lu7_EM_92987_uff_E=68.07	-4.4	0	0
6lu7_EM_93017_uff_E=281.53	-6.8	0	0
6lu7_EM_93081_uff_E=1562.49	-6.1	0	0

6lu7_EM_94149_uff_E=617.77	-6.9	0	0
6lu7_EM_94196_uff_E=314.16	-6.2	0	0
6lu7_EM_94221_uff_E=122.65	-5.2	0	0
6lu7_EM_94266_uff_E=1497.23	-5.2	0	0
6lu7_EM_96191_uff_E=252.44	-6.9	0	0
6lu7_EM_97560_uff_E=229.53	-7	0	0
6lu7_EM_98912_uff_E=394.93	-6.8	0	0
6lu7_EM_99300_uff_E=197.16	-7.2	0	0
6lu7_EM_99474_uff_E=741.60	-7	0	0
6lu7_EM_99516_uff_E=880.45	-6.9	0	0
6lu7_EM_99693_uff_E=326.11	-7.3	0	0
6lu7_EM_99938_uff_E=469.76	-7.5	0	0
6lu7_EM_100067_uff_E=737.59	-7.2	0	0
6lu7_EM_100231_uff_E=1073.13	-7.3	0	0
6lu7_EM_100332_uff_E=283.69	-6.1	0	0
6lu7_EM_100781_uff_E=713.26	-7.5	0	0
6lu7_EM_101300_uff_E=623.26	-6.8	0	0
6lu7_EM_101731_uff_E=505.04	-5.8	0	0
6lu7_EM_102667_uff_E=738.29	-5.3	0	0
6lu7_EM_107905_uff_E=319.94	-6.9	0	0
6lu7_EM_107971_uff_E=542.73	-6.8	0	0
6lu7_EM_107985_uff_E=4889.50	-7.2	0	0
6lu7_EM_114829_uff_E=185.86	-7.5	0	0
6lu7_EM_115012_uff_E=928.34	-7	0	0
6lu7_EM_115127_uff_E=732.66	-6.9	0	0
6lu7_EM_115250_uff_E=783.38	-7.2	0	0
6lu7_EM_115269_uff_E=289.50	-6.4	0	0
6lu7_EM_117440_uff_E=372.64	-6.7	0	0
6lu7_EM_117900_uff_E=505.47	-7.1	0	0
6lu7_EM_119034_uff_E=876.98	-6.7	0	0
6lu7_EM_119204_uff_E=448.72	-6.8	0	0
6lu7_EM_119205_uff_E=472.04	-6.3	0	0
6lu7_EM_119258_uff_E=429.33	-6	0	0
6lu7_EM_121313_uff_E=1058.49	-7.4	0	0
6lu7_EM_122724_uff_E=726.35	-7.1	0	0
6lu7_EM_122844_uff_E=855.65	-7.3	0	0
6lu7_EM_122850_uff_E=233.92	-7.2	0	0
6lu7_EM_124062_uff_E=170.95	-7.2	0	0
6lu7_EM_124214_uff_E=953.23	-7.4	0	0
6lu7_EM_124219_uff_E=353.67	-7.5	0	0
6lu7_EM_128108_uff_E=1024.40	-7.3	0	0
6lu7_EM_129520_uff_E=810.45	-6.1	0	0
6lu7_EM_129521_uff_E=984.56	-6.9	0	0
6lu7_EM_132415_uff_E=347.31	-7.3	0	0
6lu7_EM_133323_uff_E=492.05	-6.6	0	0
6lu7_EM_133766_uff_E=535.42	-6.8	0	0
6lu7_EM_134697_uff_E=745.24	-6	0	0
6lu7_EM_136419_uff_E=353.87	-6.4	0	0
6lu7_EM_145742_uff_E=182.22	-4.4	0	0
6lu7_EM_146798_uff_E=440.35	-6.6	0	0
6lu7_EM_147299_uff_E=483.46	-7	0	0
6lu7_EM_149600_uff_E=550.32	-7.4	0	0
6lu7_EM_151529_uff_E=931.35	-6.7	0	0
6lu7_EM_154272_uff_E=1545.99	-7.1	0	0
6lu7_EM_155011_uff_E=382.26	-7.2	0	0
6lu7_EM_155380_uff_E=462.41	-6.9	0	0
6lu7_EM_155381_uff_E=515.34	-6.7	0	0
6lu7_EM_156286_uff_E=509.39	-7.2	0	0
6lu7_EM_156875_uff_E=413.76	-7.5	0	0
6lu7_EM_157561_uff_E=2088.64	-6.4	0	0
6lu7_EM_158477_uff_E=958.72	-6.6	0	0
6lu7_EM_159795_uff_E=1059.74	-7.3	0	0
6lu7_EM_160180_uff_E=578.99	-6.2	0	0
6lu7_EM_160476_uff_E=331.53	-7.2	0	0
6lu7_EM_160481_uff_E=211.16	-7.2	0	0
6lu7_EM_160483_uff_E=376.76	-7.4	0	0

6lu7_EM_160487_uff_E=329.16	-7.4	0	0
6lu7_EM_160490_uff_E=259.28	-7.2	0	0
6lu7_EM_160497_uff_E=2091.17	-6.6	0	0
6lu7_EM_160500_uff_E=524.91	-7.3	0	0
6lu7_EM_160712_uff_E=202.48	-7.1	0	0
6lu7_EM_160817_uff_E=182.64	-7.5	0	0
6lu7_EM_161336_uff_E=739.52	-6.7	0	0
6lu7_EM_161379_uff_E=420.26	-7.1	0	0
6lu7_EM_161487_uff_E=647.75	-6.9	0	0
6lu7_EM_161665_uff_E=511.20	-6.9	0	0
6lu7_EM_162138_uff_E=872.33	-6.7	0	0
6lu7_EM_163067_uff_E=949.38	-7	0	0
6lu7_EM_164893_uff_E=258.39	-6.9	0	0
6lu7_EM_165839_uff_E=616.53	-6.7	0	0
6lu7_EM_167718_uff_E=503.12	-6.7	0	0
6lu7_EM_167825_uff_E=729.84	-6.3	0	0
6lu7_EM_168985_uff_E=608.48	-6.9	0	0
6lu7_EM_169727_uff_E=535.79	-7	0	0
6lu7_EM_173183_uff_E=573.30	-6.9	0	0
6lu7_EM_173273_uff_E=449.08	-7	0	0
6lu7_EM_173713_uff_E=479.14	-6.9	0	0
6lu7_EM_174362_uff_E=837.92	-7.1	0	0
6lu7_EM_177562_uff_E=566.07	-7.4	0	0
6lu7_EM_178034_uff_E=498.56	-7.3	0	0
6lu7_EM_178222_uff_E=732.35	-6.8	0	0
6lu7_EM_179390_uff_E=1116.22	-7.1	0	0
6lu7_EM_181384_uff_E=1041.29	-7.3	0	0
6lu7_EM_182449_uff_E=220.58	-7.5	0	0
6lu7_EM_182497_uff_E=943.83	-7.5	0	0
6lu7_EM_185605_uff_E=513.90	-7.2	0	0
6lu7_EM_185740_uff_E=223.68	-5.9	0	0
6lu7_EM_187808_uff_E=646.30	-6.9	0	0
6lu7_EM_188289_uff_E=843.41	-7.4	0	0
6lu7_EM_188316_uff_E=397.63	-7.1	0	0
6lu7_EM_188442_uff_E=438.43	-7.3	0	0
6lu7_EM_188999_uff_E=599.86	-7.3	0	0
6lu7_EM_194654_uff_E=432.12	-6.1	0	0
6lu7_EM_196978_uff_E=150.91	-7.2	0	0
6lu7_EM_197001_uff_E=638.74	-7.1	0	0
6lu7_EM_197582_uff_E=1944.57	-7.2	0	0
6lu7_EM_197857_uff_E=574.93	-7.1	0	0
6lu7_EM_222284_uff_E=590.88	-7.5	0	0
6lu7_EM_227613_uff_E=755.45	-6.7	0	0
6lu7_EM_232703_uff_E=3020.63	-4.7	0	0
6lu7_EM_234823_uff_E=545.69	-6.7	0	0
6lu7_EM_237332_uff_E=252.62	-4.6	0	0
6lu7_EM_243793_uff_E=110.97	-4.9	0	0
6lu7_EM_246983_uff_E=914.38	-7.4	0	0
6lu7_EM_251566_uff_E=805.76	-6.7	0	0
6lu7_EM_253793_uff_E=890.29	-6.8	0	0
6lu7_EM_258412_uff_E=773.28	-7.1	0	0
6lu7_EM_259846_uff_E=928.19	-6.6	0	0
6lu7_EM_276591_uff_E=430.82	-6.7	0	0
6lu7_EM_294491_uff_E=4877.37	-7.3	0	0
6lu7_EM_294711_uff_E=491.05	-4.9	0	0
6lu7_EM_307918_uff_E=304.34	-6.8	0	0
6lu7_EM_322636_uff_E=107.16	-5.7	0	0
6lu7_EM_332426_uff_E=800.44	-7	0	0
6lu7_EM_333544_uff_E=626.23	-7.1	0	0
6lu7_EM_336327_uff_E=346.46	-7.1	0	0
6lu7_EM_363209_uff_E=428.28	-7.3	0	0
6lu7_EM_414564_uff_E=237.63	-4.2	0	0
6lu7_EM_439336_uff_E=590.39	-7.4	0	0
6lu7_EM_439503_uff_E=324.93	-6.4	0	0
6lu7_EM_439533_uff_E=241.36	-7.2	0	0
6lu7_EM_439653_uff_E=417.03	-6.8	0	0

6lu7_EM_439655_uff_E=76.10	-4.6	0	0
6lu7_EM_440595_uff_E=342.56	-6.6	0	0
6lu7_EM_440967_uff_E=713.31	-5.1	0	0
6lu7_EM_440968_uff_E=633.64	-5.1	0	0
6lu7_EM_441005_uff_E=241.83	-5.9	0	0
6lu7_EM_441300_uff_E=1759.65	-6.8	0	0
6lu7_EM_441564_uff_E=475.10	-7.1	0	0
6lu7_EM_441678_uff_E=776.43	-7.3	0	0
6lu7_EM_441975_uff_E=545.68	-7.1	0	0
6lu7_EM_442001_uff_E=515.21	-7.1	0	0
6lu7_EM_442194_uff_E=543.98	-7	0	0
6lu7_EM_442350_uff_E=689.95	-7	0	0
6lu7_EM_442393_uff_E=144.71	-6.2	0	0
6lu7_EM_442437_uff_E=418.68	-7.3	0	0
6lu7_EM_442501_uff_E=147.23	-5.1	0	0
6lu7_EM_442544_uff_E=628.51	-7.2	0	0
6lu7_EM_442675_uff_E=374.79	-7.1	0	0
6lu7_EM_442695_uff_E=773.81	-5.7	0	0
6lu7_EM_442702_uff_E=236.82	-7	0	0
6lu7_EM_442741_uff_E=1904.06	-6.6	0	0
6lu7_EM_442765_uff_E=378.47	-7.4	0	0
6lu7_EM_442813_uff_E=528.17	-5.6	0	0
6lu7_EM_442827_uff_E=692.08	-6.9	0	0
6lu7_EM_442896_uff_E=738.69	-6	0	0
6lu7_EM_442985_uff_E=717.71	-6	0	0
6lu7_EM_443024_uff_E=850.21	-7.2	0	0
6lu7_EM_443027_uff_E=870.68	-6.8	0	0
6lu7_EM_443158_uff_E=118.52	-4.5	0	0
6lu7_EM_443639_uff_E=225.40	-7.4	0	0
6lu7_EM_443648_uff_E=558.56	-7.3	0	0
6lu7_EM_444539_uff_E=86.61	-5.8	0	0
6lu7_EM_445070_uff_E=148.85	-5.5	0	0
6lu7_EM_445154_uff_E=172.22	-6.9	0	0
6lu7_EM_445638_uff_E=85.31	-4.4	0	0
6lu7_EM_445639_uff_E=80.35	-5.2	0	0
6lu7_EM_445641_uff_E=103.56	-4.6	0	0
6lu7_EM_445858_uff_E=177.42	-6.1	0	0
6lu7_EM_446284_uff_E=172.66	-5.7	0	0
6lu7_EM_448438_uff_E=3509.77	-6.8	0	0
6lu7_EM_457194_uff_E=530.67	-7.2	0	0
6lu7_EM_457825_uff_E=1030.07	-6.9	0	0
6lu7_EM_466268_uff_E=207.82	-6.8	0	0
6lu7_EM_470665_uff_E=756.48	-6.8	0	0
6lu7_EM_471426_uff_E=896.18	-7.3	0	0
6lu7_EM_485186_uff_E=638.68	-7.3	0	0
6lu7_EM_503732_uff_E=730.31	-6	0	0
6lu7_EM_503734_uff_E=592.03	-5.9	0	0
6lu7_EM_517973_uff_E=80.62	-4.8	0	0
6lu7_EM_519186_uff_E=65.09	-4	0	0
6lu7_EM_519330_uff_E=125.36	-6.1	0	0
6lu7_EM_519545_uff_E=534.83	-5.9	0	0
6lu7_EM_519743_uff_E=562.91	-6.2	0	0
6lu7_EM_519857_uff_E=221.98	-6	0	0
6lu7_EM_519872_uff_E=70.85	-4.4	0	0
6lu7_EM_520710_uff_E=59.55	-4.7	0	0
6lu7_EM_527418_uff_E=740.60	-5.8	0	0
6lu7_EM_528708_uff_E=188.62	-6.3	0	0
6lu7_EM_530421_uff_E=1568.36	-5.6	0	0
6lu7_EM_530426_uff_E=1658.89	-5.9	0	0
6lu7_EM_534446_uff_E=99.80	-4	0	0
6lu7_EM_535346_uff_E=654.25	-5.9	0	0
6lu7_EM_536442_uff_E=185.24	-5.9	0	0
6lu7_EM_536595_uff_E=212.88	-6	0	0
6lu7_EM_536727_uff_E=208.75	-5.7	0	0
6lu7_EM_540645_uff_E=213.98	-6.2	0	0
6lu7_EM_541526_uff_E=190.55	-6.9	0	0

6lu7_EM_543312_uff_E=184.58	-4.3	0	0
6lu7_EM_545799_uff_E=126.49	-4.5	0	0
6lu7_EM_545889_uff_E=91.62	-4.3	0	0
6lu7_EM_545955_uff_E=88.84	-4.4	0	0
6lu7_EM_548865_uff_E=59.07	-5	0	0
6lu7_EM_550058_uff_E=478.86	-6.7	0	0
6lu7_EM_561668_uff_E=105.71	-4.8	0	0
6lu7_EM_570529_uff_E=582.59	-6.2	0	0
6lu7_EM_573721_uff_E=53.33	-4.3	0	0
6lu7_EM_578229_uff_E=173.01	-5.2	0	0
6lu7_EM_583791_uff_E=275.98	-6.2	0	0
6lu7_EM_584545_uff_E=142.22	-6.4	0	0
6lu7_EM_585744_uff_E=1354.31	-6	0	0
6lu7_EM_593889_uff_E=252.74	-6.1	0	0
6lu7_EM_594593_uff_E=1675.88	-6.2	0	0
6lu7_EM_612605_uff_E=208.84	-5.9	0	0
6lu7_EM_619267_uff_E=220.05	-6.2	0	0
6lu7_EM_630859_uff_E=623.26	-7.2	0	0
6lu7_EM_637213_uff_E=449.70	-7.4	0	0
6lu7_EM_637541_uff_E=90.97	-5.9	0	0
6lu7_EM_637542_uff_E=218.20	-5.6	0	0
6lu7_EM_637566_uff_E=110.10	-4.9	0	0
6lu7_EM_637584_uff_E=545.43	-6.8	0	0
6lu7_EM_637775_uff_E=206.17	-5.9	0	0
6lu7_EM_638072_uff_E=248.91	-6.1	0	0
6lu7_EM_643684_uff_E=131.80	-4.4	0	0
6lu7_EM_667639_uff_E=172.66	-6.7	0	0
6lu7_EM_670971_uff_E=211.83	-5.8	0	0
6lu7_EM_689043_uff_E=98.60	-5.8	0	0
6lu7_EM_689075_uff_E=109.04	-5.5	0	0
6lu7_EM_1201518_uff_E=477.10	-5.3	0	0
6lu7_EM_1268096_uff_E=550.14	-7.5	0	0
6lu7_EM_1268142_uff_E=184.78	-6	0	0
6lu7_EM_1548883_uff_E=248.44	-5.8	0	0
6lu7_EM_1549108_uff_E=157.02	-5.5	0	0
6lu7_EM_1742210_uff_E=2180.22	-6	0	0
6lu7_EM_1794427_uff_E=254.42	-6.9	0	0
6lu7_EM_2113884_uff_E=484.54	-5.6	0	0
6lu7_EM_3000341_uff_E=673.45	-6.2	0	0
6lu7_EM_3001662_uff_E=918.86	-6.9	0	0
6lu7_EM_3010930_uff_E=646.91	-7.3	0	0
6lu7_EM_3034112_uff_E=2310.32	-7.2	0	0
6lu7_EM_3035567_uff_E=428.72	-6.5	0	0
6lu7_EM_3037048_uff_E=550.54	-6.1	0	0
6lu7_EM_3037151_uff_E=509.55	-6.7	0	0
6lu7_EM_3037448_uff_E=538.47	-6.1	0	0
6lu7_EM_3037884_uff_E=522.37	-6.7	0	0
6lu7_EM_3037997_uff_E=478.47	-6.7	0	0
6lu7_EM_3039336_uff_E=490.68	-6.7	0	0
6lu7_EM_3050539_uff_E=499.66	-6.6	0	0
6lu7_EM_3080632_uff_E=630.00	-6.5	0	0
6lu7_EM_3082134_uff_E=539.38	-6.7	0	0
6lu7_EM_3082494_uff_E=1012.62	-7.5	0	0
6lu7_EM_3083575_uff_E=259.76	-7	0	0
6lu7_EM_3083592_uff_E=514.46	-6.5	0	0
6lu7_EM_3083983_uff_E=442.13	-7.3	0	0
6lu7_EM_3084224_uff_E=555.47	-7.1	0	0
6lu7_EM_3084326_uff_E=609.20	-7.3	0	0
6lu7_EM_3084713_uff_E=539.21	-7.2	0	0
6lu7_EM_3084765_uff_E=575.31	-7.1	0	0
6lu7_EM_3084770_uff_E=502.76	-7.4	0	0
6lu7_EM_3085457_uff_E=639.83	-7.3	0	0
6lu7_EM_3086461_uff_E=4896.28	-7.5	0	0
6lu7_EM_3477029_uff_E=168.39	-6.3	0	0
6lu7_EM_5241825_uff_E=447.45	-6.7	0	0
6lu7_EM_5270605_uff_E=938.06	-6.8	0	0

6lu7_EM_5273569_uff_E=194.54	-6.1	0	0
6lu7_EM_5276890_uff_E=308.17	-4.2	0	0
6lu7_EM_5280343_uff_E=380.43	-7.5	0	0
6lu7_EM_5280372_uff_E=406.64	-6.5	0	0
6lu7_EM_5280373_uff_E=372.64	-7.3	0	0
6lu7_EM_5280378_uff_E=337.60	-7.1	0	0
6lu7_EM_5280385_uff_E=358.68	-5.6	0	0
6lu7_EM_5280435_uff_E=165.89	-4.5	0	0
6lu7_EM_5280443_uff_E=233.26	-7.3	0	0
6lu7_EM_5280445_uff_E=242.10	-6.7	0	0
6lu7_EM_5280448_uff_E=410.89	-5.5	0	0
6lu7_EM_5280450_uff_E=147.99	-5.4	0	0
6lu7_EM_5280459_uff_E=587.34	-7.3	0	0
6lu7_EM_5280460_uff_E=186.56	-6	0	0
6lu7_EM_5280462_uff_E=222.28	-5.7	0	0
6lu7_EM_5280536_uff_E=169.29	-5.4	0	0
6lu7_EM_5280569_uff_E=104.71	-6.5	0	0
6lu7_EM_5280633_uff_E=251.74	-6.9	0	0
6lu7_EM_5280637_uff_E=456.82	-6.2	0	0
6lu7_EM_5280644_uff_E=94.22	-5.2	0	0
6lu7_EM_5280666_uff_E=324.01	-7.2	0	0
6lu7_EM_5280703_uff_E=2512.28	-5.7	0	0
6lu7_EM_5280781_uff_E=577.00	-6.7	0	0
6lu7_EM_5280794_uff_E=546.19	-7.3	0	0
6lu7_EM_5281600_uff_E=626.01	-7.4	0	0
6lu7_EM_5281607_uff_E=229.60	-7.4	0	0
6lu7_EM_5281617_uff_E=247.18	-7	0	0
6lu7_EM_5281623_uff_E=224.59	-6.9	0	0
6lu7_EM_5281643_uff_E=608.46	-6.7	0	0
6lu7_EM_5281649_uff_E=365.00	-7.4	0	0
6lu7_EM_5281654_uff_E=450.92	-7.5	0	0
6lu7_EM_5281659_uff_E=405.71	-5.7	0	0
6lu7_EM_5281662_uff_E=420.82	-7.5	0	0
6lu7_EM_5281665_uff_E=241.42	-7.4	0	0
6lu7_EM_5281666_uff_E=378.68	-6.9	0	0
6lu7_EM_5281672_uff_E=388.01	-7.5	0	0
6lu7_EM_5281673_uff_E=592.73	-7.2	0	0
6lu7_EM_5281674_uff_E=238.34	-7.4	0	0
6lu7_EM_5281696_uff_E=737.84	-7.2	0	0
6lu7_EM_5281699_uff_E=450.91	-7.1	0	0
6lu7_EM_5281703_uff_E=315.73	-6.8	0	0
6lu7_EM_5281704_uff_E=426.96	-7.3	0	0
6lu7_EM_5281706_uff_E=383.08	-7.1	0	0
6lu7_EM_5281708_uff_E=321.26	-7.2	0	0
6lu7_EM_5281718_uff_E=365.16	-7.5	0	0
6lu7_EM_5281750_uff_E=452.19	-7.1	0	0
6lu7_EM_5281752_uff_E=535.71	-7	0	0
6lu7_EM_5281756_uff_E=488.53	-6.6	0	0
6lu7_EM_5281762_uff_E=260.55	-7.3	0	0
6lu7_EM_5281764_uff_E=272.33	-7.1	0	0
6lu7_EM_5281766_uff_E=255.77	-6.9	0	0
6lu7_EM_5281781_uff_E=464.25	-6.9	0	0
6lu7_EM_5281800_uff_E=559.11	-6.6	0	0
6lu7_EM_5281801_uff_E=363.65	-7.4	0	0
6lu7_EM_5281803_uff_E=446.60	-7.2	0	0
6lu7_EM_5281804_uff_E=373.68	-6.8	0	0
6lu7_EM_5281805_uff_E=483.40	-7.1	0	0
6lu7_EM_5281807_uff_E=524.81	-7.3	0	0
6lu7_EM_5281810_uff_E=643.16	-7.4	0	0
6lu7_EM_5281811_uff_E=448.75	-7	0	0
6lu7_EM_5281855_uff_E=227.58	-7.5	0	0
6lu7_EM_5282073_uff_E=197.42	-7.5	0	0
6lu7_EM_5282102_uff_E=602.48	-7.2	0	0
6lu7_EM_5282149_uff_E=605.92	-6.2	0	0
6lu7_EM_5282155_uff_E=788.62	-6	0	0
6lu7_EM_5282737_uff_E=47.52	-4.9	0	0

6lu7_EM_5282743_uff_E=59.81	-4.7	0	0
6lu7_EM_5282761_uff_E=74.80	-4.3	0	0
6lu7_EM_5283324_uff_E=23.87	-4	0	0
6lu7_EM_5283335_uff_E=24.66	-3.9	0	0
6lu7_EM_5283349_uff_E=30.78	-4.2	0	0
6lu7_EM_5283384_uff_E=76.41	-4.7	0	0
6lu7_EM_5284421_uff_E=115.86	-4.3	0	0
6lu7_EM_5284499_uff_E=95.04	-4.2	0	0
6lu7_EM_5280802_uff_E=128.00	-5.3	0	0
6lu7_EM_5280804_uff_E=610.61	-6.8	0	0
6lu7_EM_5280805_uff_E=751.59	-5	0	0
6lu7_EM_5280863_uff_E=362.50	-7.4	0	0
6lu7_EM_5280899_uff_E=704.15	-7.5	0	0
6lu7_EM_5280906_uff_E=466.78	-6.9	0	0
6lu7_EM_5280933_uff_E=157.30	-4.9	0	0
6lu7_EM_5280934_uff_E=142.21	-5.4	0	0
6lu7_EM_5280961_uff_E=356.74	-7.3	0	0
6lu7_EM_5281117_uff_E=147.06	-4.8	0	0
6lu7_EM_5281119_uff_E=95.80	-4.8	0	0
6lu7_EM_5281125_uff_E=103.73	-4.6	0	0
6lu7_EM_5281220_uff_E=505.22	-7	0	0
6lu7_EM_5281223_uff_E=2122.76	-7.4	0	0
6lu7_EM_5281234_uff_E=623.80	-7.1	0	0
6lu7_EM_5281235_uff_E=709.09	-7.4	0	0
6lu7_EM_5281243_uff_E=655.10	-7.5	0	0
6lu7_EM_5281247_uff_E=2418.43	-7.4	0	0
6lu7_EM_5281377_uff_E=560.81	-7.3	0	0
6lu7_EM_5281404_uff_E=350.13	-5.9	0	0
6lu7_EM_5281408_uff_E=550.96	-6.8	0	0
6lu7_EM_5281416_uff_E=104.17	-6.5	0	0
6lu7_EM_5281426_uff_E=97.00	-6	0	0
6lu7_EM_5281514_uff_E=848.59	-5.8	0	0
6lu7_EM_5281515_uff_E=740.89	-5.9	0	0
6lu7_EM_5281516_uff_E=164.36	-5.1	0	0
6lu7_EM_5281520_uff_E=195.77	-5.9	0	0
6lu7_EM_5281522_uff_E=720.33	-5.9	0	0
6lu7_EM_5281553_uff_E=113.15	-4.7	0	0
6lu7_EM_5284507_uff_E=158.07	-5	0	0
6lu7_EM_5312508_uff_E=148.38	-5.1	0	0
6lu7_EM_5317025_uff_E=649.05	-7.2	0	0
6lu7_EM_5317238_uff_E=109.82	-5.6	0	0
6lu7_EM_5317303_uff_E=246.15	-5.8	0	0
6lu7_EM_5317306_uff_E=280.44	-6.6	0	0
6lu7_EM_5317570_uff_E=260.17	-5.9	0	0
6lu7_EM_5317750_uff_E=411.31	-7.5	0	0
6lu7_EM_5317844_uff_E=261.31	-6.2	0	0
6lu7_EM_5318042_uff_E=33.39	-4.1	0	0
6lu7_EM_5318151_uff_E=1433.79	-6.6	0	0
6lu7_EM_5318267_uff_E=589.28	-7.2	0	0
6lu7_EM_5318358_uff_E=444.69	-6.6	0	0
6lu7_EM_5318565_uff_E=274.44	-5.7	0	0
6lu7_EM_5318599_uff_E=38.64	-4	0	0
6lu7_EM_5318767_uff_E=736.07	-6.7	0	0
6lu7_EM_5319292_uff_E=591.85	-7.3	0	0
6lu7_EM_5319322_uff_E=576.06	-8.3	0	0
6lu7_EM_5319706_uff_E=138.20	-4.9	0	0
6lu7_EM_5320351_uff_E=780.08	-6.7	0	0
6lu7_EM_5320686_uff_E=664.98	-6.3	0	0
6lu7_EM_5320863_uff_E=600.81	-7.5	0	0
6lu7_EM_5321205_uff_E=440.54	-7.3	0	0
6lu7_EM_5321398_uff_E=560.16	-7.5	0	0
6lu7_EM_5321656_uff_E=874.19	-7	0	0
6lu7_EM_5321825_uff_E=270.36	-6.1	0	0
6lu7_EM_5321919_uff_E=1187.31	-7.5	0	0
6lu7_EM_5321920_uff_E=993.69	-7.4	0	0
6lu7_EM_5321977_uff_E=353.12	-6.5	0	0

6lu7_EM_5321980_uff_E=270.56	-6.8	0	0
6lu7_EM_5352470_uff_E=1651.26	-5.9	0	0
6lu7_EM_5352973_uff_E=47.62	-4.5	0	0
6lu7_EM_5353015_uff_E=242.20	-5.8	0	0
6lu7_EM_5357283_uff_E=187.18	-5.5	0	0
6lu7_EM_5362793_uff_E=111.38	-5	0	0
6lu7_EM_5362863_uff_E=137.15	-4.1	0	0
6lu7_EM_5362876_uff_E=119.24	-5.2	0	0
6lu7_EM_5362885_uff_E=683.67	-6.1	0	0
6lu7_EM_5363249_uff_E=54.76	-4.6	0	0
6lu7_EM_5363388_uff_E=62.04	-4	0	0
6lu7_EM_5363734_uff_E=1632.70	-5.8	0	0
6lu7_EM_5364471_uff_E=74.84	-3.9	0	0
6lu7_EM_5364759_uff_E=57.67	-4.4	0	0
6lu7_EM_5365004_uff_E=50.96	-4.4	0	0
6lu7_EM_5365582_uff_E=84.64	-4.6	0	0
6lu7_EM_5365585_uff_E=79.49	-4.3	0	0
6lu7_EM_5365667_uff_E=95.06	-4	0	0
6lu7_EM_5365678_uff_E=65.99	-4.4	0	0
6lu7_EM_5366078_uff_E=337.57	-5.4	0	0
6lu7_EM_5367706_uff_E=112.03	-4.7	0	0
6lu7_EM_5367785_uff_E=150.45	-5.8	0	0
6lu7_EM_5368460_uff_E=213.29	-4.7	0	0
6lu7_EM_5375252_uff_E=140.97	-6	0	0
6lu7_EM_5378284_uff_E=389.84	-6.7	0	0
6lu7_EM_5380876_uff_E=505.90	-6.9	0	0
6lu7_EM_5383438_uff_E=608.53	-6.8	0	0
6lu7_EM_5384417_uff_E=729.61	-6.9	0	0
6lu7_EM_5384527_uff_E=521.35	-6.7	0	0
6lu7_EM_5459840_uff_E=741.05	-7.5	0	0
6lu7_EM_5462193_uff_E=632.32	-4.8	0	0
6lu7_EM_5462912_uff_E=86.58	-4.6	0	0
6lu7_EM_5481646_uff_E=527.88	-7.2	0	0
6lu7_EM_5481663_uff_E=826.93	-7	0	0
6lu7_EM_5481882_uff_E=586.70	-6.7	0	0
6lu7_EM_5484202_uff_E=569.80	-6.3	0	0
6lu7_EM_5486199_uff_E=842.59	-5.6	0	0
6lu7_EM_5490351_uff_E=758.81	-6.8	0	0
6lu7_EM_5742590_uff_E=785.84	-7.2	0	0
6lu7_EM_5835713_uff_E=613.39	-6.7	0	0
6lu7_EM_6100671_uff_E=968.50	-7.2	0	0
6lu7_EM_6143289_uff_E=121.55	-5.3	0	0
6lu7_EM_6325460_uff_E=827.44	-7.2	0	0
6lu7_EM_6326060_uff_E=692.55	-6.1	0	0
6lu7_EM_6384256_uff_E=709.09	-7.4	0	0
6lu7_EM_6421261_uff_E=202.20	-5	0	0
6lu7_EM_6423815_uff_E=214.81	-5	0	0
6lu7_EM_6427091_uff_E=149.30	-5.8	0	0
6lu7_EM_6428020_uff_E=376.38	-5.7	0	0
6lu7_EM_6429077_uff_E=180.69	-5.9	0	0
6lu7_EM_6429302_uff_E=713.72	-5.6	0	0
6lu7_EM_6431456_uff_E=209.47	-6.3	0	0
6lu7_EM_6432005_uff_E=264.33	-6.1	0	0
6lu7_EM_6432312_uff_E=179.25	-5.3	0	0
6lu7_EM_6432404_uff_E=194.04	-6.3	0	0
6lu7_EM_6432648_uff_E=217.68	-6	0	0
6lu7_EM_6438572_uff_E=936.53	-6	0	0
6lu7_EM_6439187_uff_E=395.09	-6.7	0	0
6lu7_EM_6441416_uff_E=485.86	-7.2	0	0
6lu7_EM_6442194_uff_E=520.30	-7.1	0	0
6lu7_EM_6442229_uff_E=752.25	-7.3	0	0
6lu7_EM_6442619_uff_E=523.70	-7.3	0	0
6lu7_EM_6442694_uff_E=571.67	-7.1	0	0
6lu7_EM_6443046_uff_E=544.56	-7	0	0
6lu7_EM_6450452_uff_E=116.52	-4.4	0	0
6lu7_EM_6452086_uff_E=220.04	-5.8	0	0

6lu7_EM_6452639_uff_E=358.36	-7.4	0	0
6lu7_EM_6453213_uff_E=79.30	-4.8	0	0
6lu7_EM_6474309_uff_E=311.67	-6.9	0	0
6lu7_EM_6474310_uff_E=607.28	-5.7	0	0
6lu7_EM_6476333_uff_E=563.20	-5	0	0
6lu7_EM_6508206_uff_E=247.53	-6.2	0	0
6lu7_EM_6737485_uff_E=459.52	-6.1	0	0
6lu7_EM_6857493_uff_E=456.55	-6.8	0	0
6lu7_EM_6912281_uff_E=539.51	-7	0	0
6lu7_EM_6917970_uff_E=432.85	-7.1	0	0
6lu7_EM_6918391_uff_E=152.08	-5.7	0	0
6lu7_EM_6918774_uff_E=706.68	-5.2	0	0
6lu7_EM_9548595_uff_E=638.65	-6.9	0	0
6lu7_EM_9548665_uff_E=679.09	-6	0	0
6lu7_EM_9548846_uff_E=550.59	-6.7	0	0
6lu7_EM_9796304_uff_E=139.93	-5.1	0	0
6lu7_EM_9798666_uff_E=249.33	-7	0	0
6lu7_EM_9846221_uff_E=733.38	-7.3	0	0
6lu7_EM_9846222_uff_E=864.24	-8.6	0	0
6lu7_EM_9851101_uff_E=2511.90	-7.3	0	0
6lu7_EM_9859098_uff_E=382.69	-5.3	0	0
6lu7_EM_9885603_uff_E=585.25	-7.2	0	0
6lu7_EM_9912297_uff_E=752.10	-7.4	0	0
6lu7_EM_9921439_uff_E=477.15	-6.6	0	0
6lu7_EM_9938773_uff_E=1007.31	-7.1	0	0
6lu7_EM_9945785_uff_E=249.76	-6.9	0	0
6lu7_EM_10049223_uff_E=787.06	-6.8	0	0
6lu7_EM_10085878_uff_E=382.01	-6.7	0	0
6lu7_EM_10091424_uff_E=592.80	-6.7	0	0
6lu7_EM_10133609_uff_E=323.37	-6.9	0	0
6lu7_EM_10163855_uff_E=736.81	-5.2	0	0
6lu7_EM_10345799_uff_E=781.14	-7.2	0	0
6lu7_EM_10354359_uff_E=407.91	-6.5	0	0
6lu7_EM_10358881_uff_E=364.10	-7.3	0	0
6lu7_EM_10366595_uff_E=423.11	-6.6	0	0
6lu7_EM_10380207_uff_E=406.42	-5.8	0	0
6lu7_EM_10383888_uff_E=395.25	-6	0	0
6lu7_EM_10398656_uff_E=220.84	-5.7	0	0
6lu7_EM_10433924_uff_E=618.00	-7.5	0	0
6lu7_EM_10434225_uff_E=812.32	-7.5	0	0
6lu7_EM_10445823_uff_E=383.37	-6.8	0	0
6lu7_EM_10456395_uff_E=459.05	-7.1	0	0
6lu7_EM_10456516_uff_E=386.16	-6.9	0	0
6lu7_EM_10466989_uff_E=137.98	-6.6	0	0
6lu7_EM_10475115_uff_E=586.19	-6.6	0	0
6lu7_EM_10476201_uff_E=560.06	-5.8	0	0
6lu7_EM_10478550_uff_E=954.35	-5.5	0	0
6lu7_EM_10538510_uff_E=463.27	-7.2	0	0
6lu7_EM_10589811_uff_E=373.48	-6.3	0	0
6lu7_EM_10675744_uff_E=989.61	-5.2	0	0
6lu7_EM_10704181_uff_E=1678.38	-5.8	0	0
6lu7_EM_10713200_uff_E=422.84	-7.1	0	0
6lu7_EM_10781192_uff_E=455.86	-6.9	0	0
6lu7_EM_10798883_uff_E=210.82	-6.1	0	0
6lu7_EM_10814237_uff_E=2111.36	-6.8	0	0
6lu7_EM_10850329_uff_E=582.66	-6	0	0
6lu7_EM_10856614_uff_E=191.36	-5.9	0	0
6lu7_EM_10906239_uff_E=886.50	-7.4	0	0
6lu7_EM_10948757_uff_E=524.86	-6.7	0	0
6lu7_EM_10962444_uff_E=1254.89	-5.8	0	0
6lu7_EM_11034432_uff_E=222.93	-6.7	0	0
6lu7_EM_11095734_uff_E=1698.37	-6.3	0	0
6lu7_EM_11102092_uff_E=951.99	-5.8	0	0
6lu7_EM_11113483_uff_E=1086.98	-6.4	0	0
6lu7_EM_11250133_uff_E=498.07	-7.1	0	0
6lu7_EM_11253808_uff_E=196.06	-7.4	0	0

6lu7_EM_11390848_uff_E=298.77	-6.9	0	0
6lu7_EM_11537361_uff_E=640.76	-7.5	0	0
6lu7_EM_11552547_uff_E=867.34	-7.4	0	0
6lu7_EM_11623165_uff_E=376.96	-7.4	0	0
6lu7_EM_11653181_uff_E=574.56	-7.2	0	0
6lu7_EM_11667940_uff_E=738.09	-7.2	0	0
6lu7_EM_11711453_uff_E=596.41	-7	0	0
6lu7_EM_11754080_uff_E=496.15	-7.2	0	0
6lu7_EM_11822566_uff_E=569.42	-5.8	0	0
6lu7_EM_11830551_uff_E=241.87	-6.1	0	0
6lu7_EM_11968944_uff_E=745.11	-4.4	0	0
6lu7_EM_11974586_uff_E=441.51	-5.4	0	0
6lu7_EM_12004512_uff_E=2292.11	-7.5	0	0
6lu7_EM_12004524_uff_E=882.98	-4.4	0	0
6lu7_EM_12019473_uff_E=921.34	-7.2	0	0
6lu7_EM_12019474_uff_E=884.47	-7.3	0	0
6lu7_EM_12302222_uff_E=230.48	-5.6	0	0
6lu7_EM_12302502_uff_E=518.77	-6.1	0	0
6lu7_EM_12303845_uff_E=593.93	-7.1	0	0
6lu7_EM_12304985_uff_E=1741.23	-6	0	0
6lu7_EM_12309865_uff_E=638.48	-5.1	0	0
6lu7_EM_12313019_uff_E=272.33	-6.9	0	0
6lu7_EM_12313020_uff_E=173.65	-6.3	0	0
6lu7_EM_12313665_uff_E=976.48	-6.6	0	0
6lu7_EM_12314884_uff_E=676.73	-6.6	0	0
6lu7_EM_12315515_uff_E=829.59	-7	0	0
6lu7_EM_12358846_uff_E=327.66	-7.4	0	0
6lu7_EM_13818582_uff_E=914.89	-6.1	0	0
6lu7_EM_13844274_uff_E=856.33	-5.9	0	0
6lu7_EM_13844288_uff_E=498.19	-6.9	0	0
6lu7_EM_13844292_uff_E=391.86	-6.6	0	0
6lu7_EM_13844293_uff_E=491.57	-7.4	0	0
6lu7_EM_13844295_uff_E=440.69	-6.6	0	0
6lu7_EM_13845970_uff_E=462.51	-6.9	0	0
6lu7_EM_13854255_uff_E=1730.77	-6.3	0	0
6lu7_EM_13889019_uff_E=430.82	-6.5	0	0
6lu7_EM_13889020_uff_E=397.64	-6.4	0	0
6lu7_EM_13889021_uff_E=458.73	-6.7	0	0
6lu7_EM_13965876_uff_E=498.53	-7.3	0	0
6lu7_EM_13967183_uff_E=920.30	-7.1	0	0
6lu7_EM_14019178_uff_E=1057.45	-5.5	0	0
6lu7_EM_14160302_uff_E=526.85	-7.3	0	0
6lu7_EM_14180789_uff_E=569.27	-6.6	0	0
6lu7_EM_14237625_uff_E=332.30	-6.4	0	0
6lu7_EM_14262868_uff_E=614.35	-6.9	0	0
6lu7_EM_14274765_uff_E=542.06	-7.1	0	0
6lu7_EM_14283236_uff_E=492.45	-7.4	0	0
6lu7_EM_14286954_uff_E=950.77	-6	0	0
6lu7_EM_14446178_uff_E=983.92	-6.9	0	0
6lu7_EM_14520970_uff_E=220.40	-7.4	0	0
6lu7_EM_14526072_uff_E=350.93	-6.8	0	0
6lu7_EM_14562693_uff_E=309.83	-7.2	0	0
6lu7_EM_14562696_uff_E=416.45	-6.7	0	0
6lu7_EM_14605164_uff_E=1024.75	-6.3	0	0
6lu7_EM_14691941_uff_E=306.85	-7.2	0	0
6lu7_EM_14707724_uff_E=2433.83	-5.7	0	0
6lu7_EM_14730796_uff_E=205.78	-6.8	0	0
6lu7_EM_14887327_uff_E=643.49	-6.4	0	0
6lu7_EM_14992067_uff_E=934.96	-7.2	0	0
6lu7_EM_14992071_uff_E=1000.37	-5.4	0	0
6lu7_EM_15011611_uff_E=492.71	-6.3	0	0
6lu7_EM_15226622_uff_E=880.19	-6.8	0	0
6lu7_EM_15275710_uff_E=1469.51	-5.9	0	0
6lu7_EM_15459518_uff_E=969.49	-5.2	0	0
6lu7_EM_15559638_uff_E=660.89	-7.2	0	0
6lu7_EM_15560252_uff_E=281.90	-6.1	0	0

6lu7_EM_15560332_uff_E=350.66	-5.8	0	0
6lu7_EM_15658444_uff_E=405.93	-6.3	0	0
6lu7_EM_15694364_uff_E=987.91	-7.3	0	0
6lu7_EM_15736564_uff_E=1991.94	-7.2	0	0
6lu7_EM_15767709_uff_E=2245.00	-6.8	0	0
6lu7_EM_15944778_uff_E=791.28	-6.2	0	0
6lu7_EM_15983991_uff_E=724.12	-7.5	0	0
6lu7_EM_16118969_uff_E=1079.65	-5.2	0	0
6lu7_EM_20055661_uff_E=689.02	-6.3	0	0
6lu7_EM_20056194_uff_E=1163.08	-5.7	0	0
6lu7_EM_21122581_uff_E=1085.20	-4.5	0	0
6lu7_EM_21582929_uff_E=944.80	-5.7	0	0
6lu7_EM_21582934_uff_E=902.33	-6.7	0	0
6lu7_EM_21582935_uff_E=936.36	-7.3	0	0
6lu7_EM_21594133_uff_E=1040.94	-7.2	0	0
6lu7_EM_21594201_uff_E=1029.71	-7.2	0	0
6lu7_EM_21603611_uff_E=902.79	-6.5	0	0
6lu7_EM_21636239_uff_E=541.75	-6.4	0	0
6lu7_EM_21637563_uff_E=505.53	-6.8	0	0
6lu7_EM_21637743_uff_E=1218.48	-5.6	0	0
6lu7_EM_21672545_uff_E=906.64	-7.4	0	0
6lu7_EM_21672546_uff_E=1010.22	-7.1	0	0
6lu7_EM_21673419_uff_E=1417.97	-7.3	0	0
6lu7_EM_21723831_uff_E=897.91	-7	0	0
6lu7_EM_21766881_uff_E=882.33	-2.7	0	0
6lu7_EM_22297418_uff_E=578.43	-7.2	0	0
6lu7_EM_22416599_uff_E=552.35	-4.3	0	0
6lu7_EM_22676887_uff_E=75.39	-4.2	0	0
6lu7_EM_24721165_uff_E=902.37	-7.1	0	0
6lu7_EM_24814354_uff_E=1729.57	-7.2	0	0
6lu7_EM_24970641_uff_E=575.07	-7.5	0	0
6lu7_EM_25104959_uff_E=1106.53	-5.6	0	0
6lu7_EM_25756094_uff_E=188.36	-6.6	0	0
6lu7_EM_25763835_uff_E=199.05	-6.6	0	0
6lu7_EM_27282457_uff_E=89.25	-5.3	0	0
6lu7_EM_42604340_uff_E=450.86	-6.1	0	0
6lu7_EM_42604341_uff_E=251.04	-6.2	0	0
6lu7_EM_42626427_uff_E=1462.85	-4.9	0	0
6lu7_EM_44147426_uff_E=439.01	-6.2	0	0
6lu7_EM_44241258_uff_E=227.54	-7	0	0
6lu7_EM_44241259_uff_E=252.51	-5.7	0	0
6lu7_EM_44256718_uff_E=759.59	-6	0	0
6lu7_EM_44257058_uff_E=432.88	-7.3	0	0
6lu7_EM_44257110_uff_E=499.57	-7.5	0	0
6lu7_EM_44257299_uff_E=639.23	-5.2	0	0
6lu7_EM_44257531_uff_E=499.51	-6.4	0	0
6lu7_EM_44258219_uff_E=623.44	-7.4	0	0
6lu7_EM_44421646_uff_E=861.15	-6.9	0	0
6lu7_EM_44421647_uff_E=928.75	-4.6	0	0
6lu7_EM_44557350_uff_E=1114.56	-5.3	0	0
6lu7_EM_44557351_uff_E=1096.26	-6.3	0	0
6lu7_EM_44557352_uff_E=1124.88	-5.2	0	0
6lu7_EM_44557353_uff_E=911.03	-6.8	0	0
6lu7_EM_44557413_uff_E=1042.55	-7.2	0	0
6lu7_EM_44557414_uff_E=977.87	-6.8	0	0
6lu7_EM_44557415_uff_E=1101.27	-6	0	0
6lu7_EM_44557416_uff_E=923.76	-7	0	0
6lu7_EM_44557481_uff_E=1010.81	-6	0	0
6lu7_EM_44557482_uff_E=1086.57	-5.2	0	0
6lu7_EM_44568160_uff_E=539.37	-6.7	0	0
6lu7_EM_44575701_uff_E=1079.47	-5.7	0	0
6lu7_EM_44578390_uff_E=512.12	-7.4	0	0
6lu7_EM_44583694_uff_E=1145.72	-7.5	0	0
6lu7_EM_44583695_uff_E=1254.22	-6.8	0	0
6lu7_EM_44584027_uff_E=876.14	-4.7	0	0
6lu7_EM_44584030_uff_E=783.21	-7.5	0	0

6lu7_EM_44593364_uff_E=1138.13	-6.6	0	0
6lu7_EM_44715841_uff_E=588.93	-6.6	0	0
6lu7_EM_45359677_uff_E=1123.83	-6.2	0	0
6lu7_EM_45482321_uff_E=786.78	-7.5	0	0
6lu7_EM_46174030_uff_E=435.37	-5.6	0	0
6lu7_EM_46882793_uff_E=957.76	-7.2	0	0
6lu7_EM_46939340_uff_E=710.54	-7.1	0	0
6lu7_EM_50909267_uff_E=667.89	-6.9	0	0
6lu7_EM_51003489_uff_E=1080.14	-6.2	0	0
6lu7_EM_52945930_uff_E=221.19	-7.3	0	0
6lu7_EM_54067425_uff_E=810.34	-6.4	0	0
6lu7_EM_54326635_uff_E=949.03	-7.2	0	0
6lu7_EM_54675810_uff_E=205.18	-5.3	0	0
6lu7_EM_54690297_uff_E=233.66	-5.9	0	0
6lu7_EM_56680033_uff_E=762.83	-4.7	0	0
6lu7_EM_56776306_uff_E=340.57	-7.1	0	0
6lu7_EM_56951689_uff_E=952.52	-7.3	0	0
6lu7_EM_57335470_uff_E=597.68	-5.6	0	0
6lu7_EM_58552693_uff_E=89.04	-5	0	0
6lu7_EM_70697882_uff_E=600.67	-5.8	0	0
6lu7_EM_70698280_uff_E=629.07	-6.2	0	0
6lu7_EM_71307329_uff_E=604.28	-7	0	0
6lu7_EM_71437983_uff_E=402.58	-7.3	0	0
6lu7_EM_71473354_uff_E=2606.44	-7.4	0	0
6lu7_EM_71473355_uff_E=756.85	-7.1	0	0
6lu7_EM_71473390_uff_E=971.96	-5.8	0	0
6lu7_EM_73348891_uff_E=903.83	-7.1	0	0
6lu7_EM_75202444_uff_E=400.94	-7.5	0	0
6lu7_EM_76327123_uff_E=504.95	-7.1	0	0
6lu7_EM_76330776_uff_E=489.75	-6.5	0	0
6lu7_EM_76972524_uff_E=1077.68	-4.2	0	0
6lu7_EM_85350942_uff_E=3273.69	-5.7	0	0
6lu7_EM_90474067_uff_E=259.81	-6.9	0	0
6lu7_EM_91227631_uff_E=331.76	-7.3	0	0
6lu7_EM_91864462_uff_E=1004.27	-6	0	0
6lu7_EM_91895373_uff_E=589.37	-7.2	0	0

6lu7_EM_91895456_uff_E=964.43	-6.3	0	0
6lu7_EM_92016157_uff_E=497.42	-6.2	0	0
6lu7_EM_92135690_uff_E=332.88	-6.6	0	0
6lu7_EM_92469142_uff_E=254.92	-5.6	0	0
6lu7_EM_100929735_uff_E=398.11	-6.8	0	0
6lu7_EM_100967916_uff_E=1061.99	-4.7	0	0
6lu7_EM_100968221_uff_E=750.87	-5.5	0	0
6lu7_EM_100991413_uff_E=938.63	-6.6	0	0
6lu7_EM_101051955_uff_E=818.70	-4.2	0	0
6lu7_EM_101117774_uff_E=807.85	-6.6	0	0
6lu7_EM_101277340_uff_E=1021.77	-5.6	0	0
6lu7_EM_101280173_uff_E=850.66	-7.3	0	0
6lu7_EM_101281096_uff_E=800.79	-5.9	0	0
6lu7_EM_101281312_uff_E=748.65	-7.1	0	0
6lu7_EM_101289764_uff_E=820.21	-7.4	0	0
6lu7_EM_101304457_uff_E=445.10	-7	0	0
6lu7_EM_101599479_uff_E=918.48	-7.4	0	0
6lu7_EM_101616678_uff_E=812.75	-7.3	0	0
6lu7_EM_101669621_uff_E=347.91	-6.8	0	0
6lu7_EM_101688442_uff_E=149.61	-5.6	0	0
6lu7_EM_101691231_uff_E=352.84	-6.8	0	0
6lu7_EM_101701119_uff_E=841.00	-7.2	0	0
6lu7_EM_101707493_uff_E=1080.37	-7.1	0	0
6lu7_EM_101711017_uff_E=1320.08	-6.3	0	0
6lu7_EM_101862378_uff_E=785.20	-6.5	0	0
6lu7_EM_101862379_uff_E=772.73	-6.5	0	0
6lu7_EM_101905232_uff_E=716.93	-7.2	0	0
6lu7_EM_101916323_uff_E=1328.12	-6.1	0	0
6lu7_EM_101926854_uff_E=1808.55	-5.7	0	0
6lu7_EM_101936045_uff_E=710.60	-6.2	0	0
6lu7_EM_101937309_uff_E=434.05	-7.2	0	0
6lu7_EM_102004681_uff_E=647.79	-8.8	0	0
6lu7_EM_102004748_uff_E=526.01	-6.3	0	0
6lu7_EM_102049211_uff_E=713.57	-7.3	0	0
6lu7_EM_102066925_uff_E=591.89	-5.6	0	0
6lu7_EM_102067840_uff_E=349.22	-6.7	0	0
6lu7_EM_102115826_uff_E=1286.26	-5.6	0	0
6lu7_EM_102121496_uff_E=965.18	-6.6	0	0
6lu7_EM_102121497_uff_E=1045.48	-5	0	0
6lu7_EM_102121498_uff_E=914.53	-5.6	0	0
6lu7_EM_102121829_uff_E=911.04	-5	0	0
6lu7_EM_102132636_uff_E=193.60	-6.9	0	0
6lu7_EM_102132637_uff_E=467.79	-7.3	0	0
6lu7_EM_102132638_uff_E=364.98	-6.7	0	0
6lu7_EM_102316663_uff_E=757.83	-7.1	0	0
6lu7_EM_102505446_uff_E=555.35	-7.2	0	0
6lu7_EM_122209598_uff_E=405.92	-6.8	0	0
6lu7_EM_122209599_uff_E=450.81	-7.2	0	0
6lu7_EM_122391444_uff_E=2621.43	-7.3	0	0
6lu7_EM_122391445_uff_E=2128.96	-7.5	0	0
6lu7_EM_122391446_uff_E=2064.48	-7	0	0
6lu7_EM_122391447_uff_E=2101.18	-7.1	0	0
6lu7_EM_129636740_uff_E=183.94	-5.5	0	0
6lu7_EM_129670266_uff_E=359.21	-7.1	0	0
6lu7_EM_129686415_uff_E=384.81	-7.1	0	0
6lu7_EM_129834462_uff_E=725.86	-7.4	0	0
6lu7_EM_129881881_uff_E=135.57	-5	0	0
6lu7_EM_130475803_uff_E=305.08	-6.5	0	0
6lu7_EM_130475831_uff_E=722.55	-7.2	0	0
6lu7_EM_132282051_uff_E=1919.93	-7.1	0	0
6lu7_EM_132556616_uff_E=682.39	-7.3	0	0
6lu7_EM_132556617_uff_E=606.96	-7.5	0	0
6lu7_EM_132556618_uff_E=546.73	-7.3	0	0
6lu7_EM_134814038_uff_E=634.93	-7.5	0	0
6lu7_EM_135408753_uff_E=93.68	-5.7	0	0
6lu7_EM_137347660_uff_E=2285.95	-7.2	0	0
