# Supplementary material for: Screening of Potent Phytochemical Inhibitors Against SARS-CoV-2 Main Protease: An Integrative Computational Approach
Source: Front Bioinform. 2021 Oct 5;1:717141. doi: 10.3389/fbinf.2021.717141 (PMC9581031; doi:10.3389/fbinf.2021.717141)
Supplement: Supplementary file 2 [file DataSheet1.PDF]

Serial no	Plant name	Chemical name	Pubchem CID
01	*Pteris multifida* Poir.	sucrose[1]	5988
		caffeic acid[1]	689043
		pteroside[1]	10476201
		4,5-dicaffeoyl quinic acid[1]	13887346
		pteroside A[1]	169727
02	*Pueraria montana var. lobata* (Willd)Maesen et S. M. Almeida ex Sanjappa et Predeep	kakkalide[2]	5490351
		daidzin[2]	107971
		genistin[2]	5281377
		rutin[2]	5280805
		robinin[2]	5281693
		nicotiflorin[2]	5318767
		apigenin triacetate[3]	18721
		puerarin[3]	5281807
		formononetin[3]	5280378
		puerarol[3]	44257531
		genistein[3]	5280961
		ononin[3]	442813
		azukisaponin I[3]	14103656
		biochanin A[3]	5280373
		glycitein[3]	5317750

		glycitin[3]	187808
		irisolidone[3]	5281781
		luteolin[3]	5280445
		soyasaponin IV[3]	24721354
		tectorigenin[3]	5281811
		tectorigenin-7-O-xylosylglucoside[3]	100968221
		daidzein[3]	5281708
		baptisiasaponin I[3]	102317160
03	*Punica granatum* Linn	pelletierine[4]	92987
		punigluconin[4]	21637585
		casuarine[4]	9859098
		granatin A[4]	131752596
		strictinin[4]	73330
		corilagin[4]	73568
		pelargonidin-3-glucoside[4]	443648
		cyanidin-3,5-diglucoside[4]	44256718
		ursolic acid[4]	64945
		maslinic acid[4]	73659
		asiatic acid[4]	119034
		Punicalin[5]	5388496
		Gallic acid[5]	370
		friedelin[5]	91472

		ellagic acid[5]	5281855
04	*Quercus acutissima* Carruth.	epicatechin[6]	72276
		epigallocatechin[6]	72277
		epigallocatechin gallate[6]	65064
		ellagic acid[6]	5281855
		catechin[6]	9064
		gallic acid[6]	370
		ellagitannin[6]	10033935
05	*Raphanus sativus* Linn	pyrrolidine[7]	31268
		phenethylamine[7]	1001
		N-methylphenethylamine[7]	11503
		sinapine[7]	5280385
		scopoletin[7]	5280460
		erythorbic acid[7]	54675810
		caffeic[7]	689043
		β-Sitosterol[8]	222284
		stigmasterol[8]	5280794
		quercetin[8]	5280343
		kaempferol[8]	5280863
		reserpine[9]	5770
		yohimbine[9]	8969

06	*Rauvolfia verticillata* (Lour.) Baill	sarpagine[10]	12314884
		ajmaline[10]	6100671
		ajmalicine[10]	441975
		sandwicine[11]	15559808
		raunescine[11]	251566
07	*Rheum officinale* Baill	kaempferol[12]	5280863
		3',5',5,7-tetrahydroxyflavanone[12]	52945930
		gallic acid[12]	370
		4-Hydroxybenzoic acid[12]	135
		aloe-emodin[13]	10207
		rhein[13]	10168
		chrysophanol[13]	10208
		physcion[13]	10639
		emodin[13]	3220
		rhaponticin[14]	637213
		epicatechin-3-O-gallate[14]	107905
		quercetin-3-O-l-rhamnoside[15]	5280459
		Gallic acid[16]	370
		Methyl gallate[16]	7428
		Ellagic acid[16]	5281855
		Tellimagrandin I[16]	442690

08	*Rosa chinensis* Jacq	Tellimagrandin II[16]	151590
		Rugosin A[16]	16132354
		Rugosin A methyl ester[16]	102045132
		Isoquercitrin[16]	5280804
		Isoquercitrin[16]	5280804
		Quercetin-3- *O* -xyloside[16]	5320863
		Quercetin-3- *O* -arabinoside[16]	12309865
		Kaempferol-3- *O* -rhamnoside[16]	5835713
		kaempferol-3- *O* -galactoside[16]	5462193
		kaempferol-3- *O* -arabinoside[16]	5481882
		rutin[16]	5280805
		quinic acid[16]	6508
		5-galloylquinic acid[16]	14520970
		casuarictin[16]	73644
		glucose[16]	5793
		tiliroside[17]	5320686
		kaempferol[17]	5280863
		quercetin[17]	5280343
		catechin[17]	9064
		eudesmin[17]	234823
		loliolide[18]	100332
		daucosterol[18]	5742590

09	*Rosa laevigata* Michx	euscaphic acid[18]	471426
		beta-sitosterol[18]	222284
		betulinic acid[18]	64971
		kajiichigoside[18]	14019178
		rubuside[18]	44557350
		tomentic acid[18]	73193
		rosamutin[18]	21122581
		nigaichigoside[18]	16118969
		ketol[19]	14367662
		diethyl malate[19]	24197
		p-coumaric acid[19]	637542
		catechin[19]	9064
		kaemferol[19]	5280863
		quercetin[19]	5280343
		2,4-decadienal[20]	5283349
		ethyl laurate[20]	7800
		hexanoic acid[20]	8892
		citronellyl isobutyrate[20]	60985
		3-methyl-5-propylnonane[20]	545955
		ethyl tetradecanoate[20]	31283
		octanoic acid[20]	379

		6,10,14-trimethyl-2-pentadecanone[20]	10408
		ethyl pentadecanoate[20]	38762
		methyl palmitate[20]	8181
		ethyl palmitate[20]	12366
		ethyl 9-hexadecenoate[20]	5364759
		1-acetoxyhexadecane[20]	12393
		heptadecane[20]	12398
		methyl stearate[20]	8201
		methyl 11-octadecenoate[20]	5364432
		ethyl octadecanoate[20]	8122
		ethyl oleate[20]	5363269
		ethyl linoleate[20]	5282184
		oleyl alcohol[20]	5284499
		phytol[20]	5280435
		9-Hexacosene[20]	5363630
		hexacosane[20]	12407
		cis-9-hexadecenoic acid[20]	445638
		heptadecanoic acid[20]	10465
		linoleic acid[20]	5280450
		linolenic acid[20]	5280934

		Heptaethylene-glycol[20]	79718
		1,2-Hexadecene epoxide[20]	23741
		rubuside J[21]	44557482
		polystachyol[22]	92016157
		liquiritigenin[22]	114829
		syringaldehyde[22]	8655
		vanillin[22]	1183
		4-hydroxybenzaldehyde[22]	126
10	*Rubia cordifolia* L.	purpurin[23]	6683
		munjistin[23]	160476
		xanthopurpurin[23]	196978
		pseudopurpurin[23]	442765
		Alizarin[23]	6293
		mollugin[23]	124219
		β- sitosterol[23]	222284
		daucosterol[23]	5742590
		1, 3, 6-trihydroxy-2-methyl-9, 10anthra-quinone-3-O-(6'-Oacetyl)-α-L-rhamnosyl (1→2)-βD-glucoside[23]	57335470
		1-hydroxy 2-methyl anthraquinone[23]	160817
		nordamnacanthal[23]	160712
		physcion[23]	10639

		rubiatriol[23]	21582929
		1, 3- dimethoxy 2-carboxy anthraquinone[23]	129670266
		1, 4-dihydroxy 2 methylanthraquinone[23]	99300
		1, 5-dihydroxy 2 methylanthraquinone[23]	182449
		rubicoumaric acid[23]	5377693
		rubifolic acid[23]	91895456
		lucidin primeveroside[23]	160180
		rubiarbonol A[23]	12019473
		rubiarbonol B[23]	12019474
		rubiarbonol C[23]	21672545
		rubiarbonol D[23]	21672546
		rubiarbonol E[23]	21582934
		rubiarbonol F[23]	21582935
		furomollugin[23]	10354359
		rubilactone[23]	132415
		eugenol[23]	3314
		Epoxymollugin[23]	24814354
		atraric acid[23]	78435

		Rubiprasin A[24]	21594201
		Rubiprasin B[24]	21594133
		scopoletol[24]	5280460
		rubiadin-1-methyl ether[25]	96191
		rubiadin[25]	124062
11	*Rubus ellipticus var. obcordatus (*Franch.) Focke	Rubuside A[26]	44557350
		Rubuside B[26]	44557351
		Rubuside C[26]	44557352
		Rubuside D[26]	44557353
		Rubuside E[26]	44557413
		Rubuside F[26]	44557414
		Rubuside G[26]	44557415
		Rubuside H[26]	44557416
		Rubuside I[26]	44557481
		Rubuside J[26]	44557482
		quadranoside VIII[26]	10675744
		sericoside[26]	76972524
		sericic acid[26]	124214
		buergericic acid[26]	46882793

		pinfaensin[26]	190934
		rosamutin[26]	21122581
		kaji-ichigoside F1[26]	14019178
		niga-ichigoside F1[26]	16118969
		trachelosperoside A1[26]	21637743
		pedunculoside[26]	14286954
		ziyu-glycoside[26]	71609288
		euscaphic acid[26]	471426
		gallic acid[27]	370
		chlorogenic acid[27]	1794427
		catechin[27]	9064
		caffeic acid[27]	689043
12	*Rumex dentatus* Linn.	Palmitic acid methyl ester[28]	8181
		Stearic acid methyl ester[28]	8201
		Linoleic acid[28]	5280450
		Endocrocin[28]	160483
		chrysophanol[28]	10208
		emodin[28]	3220
		physcion[28]	10639

		p-hydroxybenzoic acid[29]	135
		syringic acid[29]	10742
		vanillin[29]	1183
		benzoic acid[29]	243
		ferulic acid[29]	445858
		cinnamic acid[29]	444539
		kaempferol 3-O-rutinoside[30]	5318767
		isorhamnetin 3-O-rutinoside[30]	5481663
13	*Sabia yunnanensis* Franch.	ferulic acid[31]	445858
		syringic acid[31]	10742
		sinapic acid[31]	637775
		tyrosol[31]	10393
		daphnetin[31]	5280569
		p-hydroxybenzoic acid[31]	135
		p-hydroxycinnamic acid[31]	637542
		glycosmisic acid[31]	14274765
		fraxetin[31]	5273569
		2,5-dihydroxybenzoic acid[31]	3469
		skimmin[31]	99693
		umbelliferone[32]	5281426

		salicylic acid[32]	338
		emodin[32]	3220
		oleanolic acid[32]	10494
		5-hydroxymethyl-furaldehyde[32]	237332
		scopoletin[32]	5280460
		isofraxidin[32]	5318565
		cleomiscosin D[32]	13965876
		vanillic acid[32]	8468
		quercetin[32]	5280343
		succinic acid[32]	1110
		daucosterol[32]	5742590
		uracil[32]	1174
14	*Salix matsudana* Koidz	3,6-dimethoxy-5 ,7-dihydroxyflavone[33]	5481646
		3,5,7-trimethoxyflavone[33]	117900
		5-hydroxy7 ,8-dimethoxyflavone[33]	188316
		5-hydroxy-3,7,8,2 -tetramethoxyflavone[33]	14887327
		palmitic acid[33]	985
		β- sitosterol[33]	222284
		salicin[33]	439503

		caffeic acid[33]	689043
		matsudone A[34]	70697882
		luteolin[34]	5280445
		isoquercitrin[34]	5280804
		7-methoxyflavone[34]	466268
		luteolin 7-O-glucoside[34]	5280637
		4',7-dihydroxyflavone[34]	5282073
		leonuriside A[34]	14237625
		piceoside[34]	92123
15	*Sarcococca ruscifolia* Stapf	sarcovagine D[35]	102049211
		pachysamine H[35]	54326635
		pachysamine A[35]	197857
		terminaline [35]	177562
		sarcorucinine E[36]	132556616
		sarcorucinine F[36]	132556617
		sarcorucinine G[36]	132556618
		pachysamine M[36]	46939340
		epipachysamine D[36]	10433924

		ponasteroneA[37]	115127
		posterone[37]	165839
		calonysterone[37]	101281312
		lupeol[37]	259846
16	*Schefflera arboricola* Hayata	ursolic acid[38]	64945
		oleanolic acid [38]	10494
		3-oxo oleanolic acid[38]	470665
		sericic acid[38]	124214
		b-sitosterol[38]	222284
		α-Pinene[39]	6654
		β-Pinene[39]	14896
		Myrcene[39]	31253
		o-Cymene[39]	10703
		Limonene[39]	22311
		β-Phellandrene[39]	11142
		Cryptone[39]	92780
		trans-Carveol[39]	94221
		Carvone[39]	7439
		α-Cubebene[39]	86609
		α-Copaene[39]	19725

		β-Bourbonene[39]	62566
		β-Cubebene[39]	93081
		β-Caryophyllene[39]	5281515
		Aromadendrene[39]	91354
		α-Humulene[39]	5281520
		γ-Muurolene[39]	12313020
		Germacrene D[39]	5317570
		β-Selinene[39]	442393
		γ-Amorphene[39]	12313019
		α-Selinene[39]	10856614
		γ-Cadinene[39]	6432404
		δ-Cadinene[39]	441005
		cis-Calamenene[39]	6429077
		Spathulenol[39]	92231
		Caryophyllene oxide[39]	1742210
		Humulene epoxide I[39]	5352470
		Humulene epoxide II[39]	10704181
		1-epi-Cubenol[39]	519857

		Caryophylla-3(15),7(14)-dien-6-ol[39]	527418
		trans-Calamenen-10-ol[39]	10798883
		E-Caryophyllene[39]	5281522
		α-Cyperone[39]	6452086
		Nootkatone[39]	1268142
17	*Schisandra henryi* C.B.Clarke	henricine A[40]	42604340
		henricine B[40]	42604341
		wulignan A2[40]	13844292
		epiwulignan A1[40]	13844293
		deoxyschisandrin[40]	43595
		wulignan A1[40]	13844295
		schisantherin A[40]	151529
		schisandrol A[40]	11102092
		furofuran[41]	22416599
		furan[41]	8029
		dibenzylbutyrolactone[41]	99938

		dibenzocyclooctadiene[41]	307918
		schisandrin[41]	23915
		schisandrin C[41]	443027
		gomisin A[41]	3001662
		gomisin G[41]	14992067
		schisantherin B[41]	6438572
		lanostane[41]	9548665
		cycloartane[41]	160497
		benzoylgomisin Q[41]	14605164
		isoanwulignan[41]	15658444
		schisantherin E[41]	13844274
		angeloylgomisin Q[41]	14992071
		gomisin F[41]	51003489
		angeloylgomisin O[41]	91864462
		schisantherin D[41]	163067

		enshicine[41]	13844288
		kadsuric acid[41]	5384417
		nigranoic acid[41]	10814237
		isoschisandrolic acid[41]	137347660
18	*Scutellaria discolor* Colebr	5,7-Dihydroxy-8,2_-dimethoxyflavone[42]	13889021
		5,7-Dihydroxy-8,2_,6_-trimethoxyflavone[42]	14180789
		7-Hydroxy-5,8,2_-trimethoxyflavone[42]	21637563
		5,2_-Dihydroxy-6,7,6_-trimethoxyflavanone[42]	13889020
		5,7-Dihydroxy-8,2_-dimethoxyflavanone[42]	101669621
		7-Hydroxy-5,8,2_-trimethoxyflavanone[42]	146156172
		2_,4_-Dihydroxy-2,3,6_-trimethoxychalcone[42]	21636239
		wogonin[2]	5281703
		norwogonin[2]	5281674
		5,2'-Dihydroxy-7,8,6'-trimethoxyflavanone[2]	13889019
		5,7,2'-trihydroxy-8-methoxyflavone[2]	5321205

		Pinocembrin[2]	68071
		chrysin[2]	5281607
		apigenin[2]	5280443
		luteolin[2]	5280445
19	*Senecio scandens* Buch.Ham. ex D. Don	Quercetin[43]	5280343
		Kaempferol[43]	5280863
		Hyperoside[43]	5281643
		Linarin[43]	5317025
		Rutin[43]	5280805
		Lupenone[43]	92158
		Emodin[43]	3220
		Isorhamnetin[43]	5281654
		Luteolin[43]	5280445
		Isoquercitrin[43]	5280804
		Adonifoline[43]	15736564
		Senecionine[43]	5280906
		Seneciphylline[43]	5281750

		Seneciphylline N-oxide[43]	6442619
		Neoplatyphylline[43]	6912281
		7-Tigloylplatynecine[43]	6428020
		Jacobine[43]	442741
		Usaramine[43]	5281756
		Senecionine N-oxide[43]	5380876
		Jacozine N-oxide[43]	132282051
		Senkirkine[43]	5281752
		Monocrotaline[43]	9415
		1,2-dihydroxybenzene[43]	289
		P-hydroxyphenyl acetic acid[43]	127
		Vanillic acid[43]	8468
		Pyromucic acid[43]	6919
		Hydroquinone[43]	785
		Protocatechuic acid ethyl ester[43]	77547

		Caffeic acid[43]	689043
		Gentisic acid[43]	3469
		P-hydroxybenzoic acid[43]	135
		P-hydroxycinnamic acid[43]	637542
		Trans-caffeic acid[43]	2518
		Salicylic acid[43]	338
		4-Hydroxy-3,5-dimethoxybenzoicacid[43]	10742
		Sinapic acid[43]	637775
		1-Hydroxy-4-oxo-2,5-cyclohexadiene-1-acetic acid[43]	6453213
		Protocatechuic acid[43]	72
		Chlorogenic acid[43]	1794427
		Coumaroylquinic acid[43]	9945785
		Feruloylquinic acid[43]	10133609
		Dicaffeoylquinic acid[43]	12358846
		Ferulic acid[43]	445858
		Coniferin[43]	5280372

		α-Pinene[43]	440968
		Cyclohexane[43]	8078
		β-Pinene[43]	440967
		1,3-Cyclohexadiene[43]	11605
		(E)/(Z)ocimene[43]	5281553
		α-Terpinene[43]	7462
		γ-Terpinene[43]	7461
		Caryophyllene[43]	5281515
		Nerolidol[43]	5284507
		Methyl salicylate[43]	4133
		γ-Elemene[43]	6432312
		α-Farnesene[43]	5281516
		2-Pentadecanone[43]	61303
		2-Naphthylamine[43]	7057
		Nonacosane[43]	12409

		Tetracosane[43]	12592
		Triacontane[43]	12535
		Eicosane[43]	8222
		Heptadecane[43]	12398
		Hexadecane[43]	11006
		Octacosane[43]	12408
		Pentadecanoic acid[43]	13849
		Hexadecanoic acid[43]	985
		9-Octadecynoic acid[43]	68167
		(3E)-3-icosene[43]	5365051
		Oleic acid[43]	445639
		1-Nonene[43]	31285
		1-Ethenyl-1-methyl-2,4-bis(1-methylethenyl)-cyclohexane[43]	6918391
		α-Bergamotene[43]	6429302
		1,4,9,9-tetramethyl-4,7-methanoazulene[43]	101731

		4,7-Dimethyl-1-(1-methylethyl)-Naphthalene[43]	528708
		Sesquirosefuran[43]	5366078
		Isoaromadendrene epoxide[43]	534398
		Caryophyllene oxide[43]	1742210
		Trans-2-Undecen-1-ol[43]	5365004
		1,4-Dimethyl-7-(1-methylethenyl)-azulene[43]	3083592
		Humulane-1,6-dien-3-ol[43]	5353015
		Tetradecanoic acid[43]	11005
		4,5-Bis(hydroxymethyl)-3,6-dimethylcyclohexene[43]	578229
		(2Z,6E)-farnesol[43]	1549108
		Phthalic acid[43]	1017
		(Z)-9,17-Octadecadienal[43]	5365667
		9,12,15-Octadecatrienal[43]	5283384
		Phenanthrene[43]	995
		Hexahydrofarnesyl acetone[43]	10408
		Phytol[43]	5280435

		Linoleic acid[43]	5280450
		Heneicosane[43]	12403
		Heptacosane[43]	11636
		Jacaranone[43]	73307
		Senecio lactone[43]	92469142
		Oleanolic[43]	6453932
		β-Amyrenonol[43]	20055661
		β-Amyrin[43]	73145
		β-Sitosterol[43]	222284
		stigmasterol[43]	5280794
		β-Sitostanol[43]	6743
		α-Sitosterol[43]	9548595
		24-Hydroxycycloart-25-en-3-one[43]	15767709
		24-methyl-cholestan-3-one[43]	14160302
		Ergosta-4,6,8(14),22-tetraen-3-one[43]	6441416
		(E)-24-methyl-cholest-22-en-3-one[43]	14283236

		6-Hydroxystigmast-4-en-3-one[43]	71307329
		-Pinoresinol[43]	73399
		-epi-Pinoresinol[43]	637584
		Tortoside A[43]	101701119
		Caruilignan D[43]	10850329
		lirioresinol-A[43]	10049223
		stigmasta-4,22-dien-3-one[44]	6442194
		stigmast-4-en-3-one[44]	5484202
		cis-ferulic acid[44]	1548883
		methyl ferulate[44]	5357283
		4-hydroxy-3-methoxyacetophenone[44]	2214
		3-methoxyisonicotinic acid[44]	27282457
		pentacosanoic acid[45]	10468
		sucrose[45]	5988
20	*Senna occidentalis* (L.)	aloe-emodin[46]	10207
		emodin[46]	3220

		kaempferol[46]	5280863
		obtusifolin[46]	3083575
		obtusin[46]	155380
		physcion[46]	10639
		anthraquinones[46]	6780
		apigenin[46]	5280443
		campesterol[46]	173183
		chrysoobtusin[46]	155381
		chrysophanic acid[46]	10208
		chrysoeriol[46]	5280666
		funiculosin[46]	10151
		quercetin[46]	5280343
		rhein[46]	10168
		rubrofusarin[46]	72537
		sitosterol[46]	222284
		Nonanoic acid[46]	8158
		Dodecanoic acid[46]	3893
		Tetradecanoic acid[46]	11005

		n-Hexadecanoic acid[46]	985
		10-Octadecenoic acid methyl ester[46]	25642
		9,12-Octadecadienoic acid[46]	5280450
		Oleic acid 3-hydroxypropyl ester[46]	5352775
		Hexanoic acid[46]	8892
		Octanoic acid[46]	379
		n-Decanoic acid[46]	2969
		9-Oxononanoic acid[46]	75704
		3-ethyl-2-hydroxy-2-cyclopenten-1-one[46]	62752
		Tetradecanoic acid[46]	11005
		Phytol[46]	5280435
		linoleic acid	5280450
		linolenic acid[47]	5280934
		mannitol[47]	6251
		matteucinol[47]	160490
		Cassiaoccidentalin A[47]	503734

		Cassiaoccidentalin B[47]	70698280
		Cassiaoccidentalin C[47]	44258219
		1,8-dihydroxyanthraquinone[47]	2950
		bianthraquinone[47]	6737485
		germichrysone[47]	90474067
		1,8-dihydroxy-2-methyl anthraquinone[47]	11253808
		N-methylmorpholine[47]	7972
		galactomannan[47]	439336
		Helminthosporin[47]	97560
21	*Senna tora* (L.) Roxb	γ-butyrolactone[48]	7302
		methyl-4-methyloctanoate[48]	519186
		1-pyrrolidinylacetic acid[48]	414564
		p-vinylguaiacol[48]	332
		undec-10-enoic acid[48]	5634
		1,E-8,Z-10-pentadecatriene[48]	5365582
		lauric acid[48]	3893
		Z-9-tetradecenal[48]	5364471
		myristic acid[48]	11005

		6,9-pentadecadien-1-ol[48]	548865
		methyl-14-methylpentadecanoate[48]	21205
		1,E-11,Z-13-octadecatriene[48]	5365585
		isopropylmyristate[48]	8042
		2-dodecyl-1,3-propanediol[48]	534446
		palmitic acid[48]	985
		octyl-10-undecenoate[48]	534598
		9,12-octadecadien-1-ol[48]	5462912
		vinyl stearyl ether[48]	13585
		stearic acid, methyl ester[48]	8201
		n-nonadecanol[48]	80281
		stearic acid[48]	5281
		cis-oleic acid[48]	445639
		1,1-bis (dodecyloxy)hexadecane[48]	41920
		methionine[49]	6137
		tryptophan[49]	6305
		torachrysone gentiobioside[49]	503732
		Lignoceric acid[49]	11197

		Linoleic acid[49]	5280450
		Emodin[50]	3220
		stigmasterol[50]	5280794
		β-sitosterol-β-D-glucoside[50]	5742590
		succinic acid[50]	1110
		d-tartaric acid[50]	439655
		uridine[50]	6029
		ononitol monohydrate[50]	129711244
		euphol[50]	441678
		obtusifolin[50]	3083575
		rubrofusarin[50]	72537
		aurantio-obtusin[50]	155011
		rubrofusarin-6-β-gentiobioside[50]	14189963
		torachrysone gentiobioside[50]	503732
		toralactone[50]	5321980
		torachrysone[50]	5321977
		cassitoroside[50]	131753095
		isorubrofusarin gentiobioside[50]	85211061
		4,8-dimethyl-undecane[51]	28454
		5-ethyl-2,2,3-trimethyl-heptane[51]	545799

		3-ethyl-5-methyl-1-propyl-cyclohexane[51]	58552693
		Eicosane[51]	8222
		Cyclohexyl-benzene[51]	13229
		Linoleic acid ethyl ester[51]	5282184
		Phthalic acid isobutyl octyl ester[51]	6423815
		Oxalic acid isobutyl heptadecyl ester[51]	6420712
22	*Sida acuta* Burm. f.	cryptolepine[52]	82143
		quindoline[52]	98912
		quindolinone[52]	129686415
		cryptolepinone[52]	178034
		ecdysterone[52]	5459840
		beta-sistosterol[52]	222284
		Evofolin B[52]	5317306
		loliolid[52]	100332
		4-ketopinoresinol[52]	44578390
		quinazoline[53]	9210
		choline[53]	305

		betaine[53]	247
		vasicine[53]	72610
		Ephedrine[54]	9294
		11-Methoxyquindoline[54]	10538510
		Hentriacontane[54]	12410
		Nonacosane[54]	12409
		Pristane[54]	15979
		Phytane[54]	12523
		Sterculic acid[54]	12921
		Malvalic acid[54]	10416
		Linoleic acid[54]	5280450
		Scopoletin[54]	5280460
		Heraclenol[54]	73253
		Acanthoside B[54]	443024
		Sinapic acid[54]	637775
		Syringic acid[54]	10742
		Vanillic acid[54]	8468

		Di-(2-ethylhexyl)phthalate[54]	8343
		Cholesterol[54]	5997
		Stigmasterol[54]	5280794
		Stigmast-7-enol[54]	3080632
		Vomifoliol[54]	5280462
		Taraxasterone[54]	14485465
		α-amyrine[54]	73170
23	*Stephania delavayi* Diels	Stephodeline[55]	102066925
		Delavayine A[55]	10380207
		Delavayine B[55]	10713200
		Delavayine C[55]	10589811
24	*Taxillus sutchuenensis* (Lecomte) Danser	isosakuranetin[56]	160481
		viscumneoside I[56]	42608078
		quercetin 3,3′,4′-trimethyl ether[56]	5383438
		kaempferol-3,7-bisrhamnoside[56]	5486199
		hannokinol[56]	11034432
		meso-hannokinol[56]	25763835
		oleanic acid[56]	10494
		tremulacin[56]	442544
25	*Tetrastigma hypoglaucum* Planch.	β-sitosterol[57]	222284

		palmitic acid[57]	985
		pentacosane[57]	12406
		daucosterol[57]	5742590
		resveratrol[57]	445154
		gallic acid[57]	370
		ethyl gallate[57]	13250
		catechin[57]	9064
26	*Thunia alba* (Lindl.) Rchb. f.	lusianthridin[58]	442702
		coelonin[58]	11390848
		thunalbene[58]	25756094
		batatasin-III[59]	10466989
		3,7-dihydroxy-2,4-dimethoxyphenanthrene[59]	10445823
		cirrhopetalanthrin[59]	442695
		Flavanthrin[59]	102004681
27	*Toricellia tiliifolia* DC	betulinic acid[60]	64971
		daucosterol[60]	5742590
		gallic acid[60]	370
		3, 5-dimethoxybenzaldehyde[60]	81747
		4-hydroxy-3-methoxycinnamaldehyde[60]	5280536
		3, 5-dimethoxy-4-hydroxycinnamaldehyde[60]	5376357

		syringaresinol[60]	100067
28	*Tradescantia pallida* (Rose) D.R.Hunt	spathulenol[61]	92231
		caryophyllene oxide[61]	1742210
		b-caryophyllene[61]	5281515
		a-copaene[61]	70678558
29	*Tecoma capensis* (Thunb.)	quercetin 4'-O-methyl ether[62]	5281699
		rutin[62]	5280805
		myricetin[62]	5281672
		kaempferol 4'-O-methyl ether[62]	5281666
		luteolin[62]	5280445
		apigenin[62]	5280443

Reference:

1. Harinantenaina, L. & Matsunami, Æ. K. Chemical and biologically active constituents of Pteris multifida. 452–455 (2008) doi:10.1007/s11418-008-0265-9.

2. Hayata, Y. NII-Electronic Library Service. *Chem. Pharm. Bull.* 2091 (2002).

3. Sanjappa, W., Flavonoid, P., Tungmunnithum, D., Intharuksa, A. & Sasaki, Y. Traditional Uses and Potential Biological Activities. (2020).

4. Sharrif, M. M. & Hamed, H. K. Chemical composition of the plant Punica granatum L. (Pomegranate) and its effect on heart and cancer. *J. Med. Plants Res.* **6**, 5306–5310 (2012).

5. Haque, N., Sofi, G., Ali, W., Rashid, M. & Itrat, M. A comprehensive review of phytochemical and pharmacological profile of Anar (Punica granatum Linn): A heaven's fruit. *J. Ayurvedic Herb. Med.* **1**, 22–26 (2015).

6. Burlacu, E., Nisca, A. & Tanase, C. A Comprehensive Review of Phytochemistry and

Biological Activities of Quercus Species. 1–24.

7. Gutiérrez, R. M. P. & Perez, R. L. Raphanus sativus (Radish): their chemistry and biology. *ScientificWorldJournal.* **4**, 811–837 (2004).

8. No, P. *et al.* EXTRACTS AND THEIR INSECTICIDAL ACTIVITY AGAINST MEALYBUG , PHENACOCCUS. **8**, 6–11 (2019).

9. Duan, W. J. *et al.* Preparative separation of two alkaloids from devil pepper radix (Rauvolfia verticillata [Lour.] Baill.) by pH-zone-refining counter-current chromatography. *Acta Chromatogr.* **30**, 81–84 (2018).

10. Hong, B., Li, W. J., Song, A. H. & Zhao, C. J. Determination of indole alkaloids and highly volatile compounds in rauvolfia verticillata by HPLC-UV and GC-MS. *J. Chromatogr. Sci.* **51**, 926–930 (2013).

11. Hong, B., Li, W. J. & Zhao, C. J. Chemical constituents of Rauvolfia verticillata. *Yaoxue Xuebao* **47**, 764–768 (2012).

12. Gao, L., Xu, X. & Yang, J. Chemical constituents of the roots of Rheum officinale. *Chem. Nat. Compd.* **49**, 603–605 (2013).

13. Cai, Y., Sun, M., Xing, J. & Corke, H. Antioxidant phenolic constituents in roots of Rheum officinale and Rubia cordifolia: Structure-radical scavenginq activity relationships. *J. Agric. Food Chem.* **52**, 7884–7890 (2004).

14. ZHENG, Q. *et al.* Review of Rhubarbs: Chemistry and Pharmacology. *Chinese Herb. Med.* **5**, 9–32 (2013).

15. Qing, L. Sen *et al.* Identification of flavonoid glycosides in Rosa chinensis flowers by liquid chromatography-tandem mass spectrometry in combination with 13C nuclear magnetic resonance. *J. Chromatogr. A* **1249**, 130–137 (2012).

16. Luo, Y. *et al.* One injection to profile the chemical composition and dual-antioxidation activities of Rosa chinensis Jacq. *J. Chromatogr. A* **1613**, 460663 (2020).

17. Liu, Y. *et al.* Catechins and lignan from the flower buds of Rosa chinensis Jacq. *Phytochem. Lett.* **38**, 46–48 (2020).

18. Mehboob, H. *et al.* A Review on Secondary Metabolites of Rosa laevigata Michaux: An Important Medicinal Plant. *Biochem. Anal. Biochem.* **06**, 3–5 (2017).

19. Constituents, T. C. & Part, A. ~IIIII~m~1I111111111111111 11111111111. 11–13 (1991).

20. Yang, J. *et al.* Chemical Composition and Aromatic Profiles of Essential Oil from Rosa laevigata by GC-MS/GC-O Analysis. *Adv. J. Food Sci. Technol.* **11**, 147–152 (2016).

21. Li, S. P. *et al.* Flavonoids and triterpenoids from the roots of Rosa laevigata. *J. Mex. Chem. Soc.* **58**, 374–377 (2014).

22. Li, X. *et al.* Antioxidant compounds from Rosa laevigata fruits. *Food Chem.* **130**, 575–580 (2012).

23. Siril, E. A. & Devi, P. M. Traditional and Modern Use of Indian Madder (Rubia cordifolia L .): An Overview. *Int. J. Pharm. Sci. Rev. Res.* **25**, 154–164 (2014).

24. Patil, R., Mohan, M., Kasture, V. & Kasture, S. Rubia cordifolia: a review. *Orient. Pharm. Exp. Med.* **9**, 1–13 (2009).

25. Verma, A., Kumar, B., Alam, P., Singh, V. & Kumar Gupta, S. Rubia Cordifolia-a Review on Pharmaconosy and Phytochemistry. *Int. J. Pharm. Sci. Res.* **7**, 2720 (2016).

26. Li, W. *et al.* Triterpenoid saponins from Rubus ellipticus var. obcordatus. *J. Nat. Prod.* **72**, 1755–1760 (2009).

27. Badhani, A., Rawat, S., Bhatt, I. D. & Rawal, R. S. Variation in Chemical Constituents and Antioxidant Activity in Yellow Himalayan (Rubus ellipticusSmith) and Hill Raspberry (Rubus Niveus Thunb.). *J. Food Biochem.* **39**, 663–672 (2015).

28. Elfotoh, M. A. A. *et al.* Lipophilic constituents of Rumex vesicarius L. and Rumex dentatus L. *Antioxidants* **2**, 167–180 (2013).

29. Elzaawely, A. A. & Tawata, S. Antioxidant capacity and phenolic content of Rumex dentatus L. Grown in Egypt. *J. Crop Sci. Biotechnol.* **15**, 59–64 (2012).

30. Hawas, U. W., Ahmed, E. F., Abdelkader, A. F. & Taie, H. A. A. A. Biological activity of flavonol glycosides from Rumex dentatus plant, an Egyptian Xerophyte. *J. Med. Plants Res.* **5**, 4239–4243 (2011).

31. Deng, Y., Zheng, Y., Chen, B., Zhang, G. L. & Wu, F. E. Sabian, a novel flavonoid from Sabia yunnanensis. *J. Asian Nat. Prod. Res.* **7**, 741–745 (2005).

32. Deng, Y., Tang, T. J., Li, X. & Wu, F. A new aporphine alkaloid form Sabia yunnanensis. *Nat. Prod. Res.* **21**, 28–32 (2007).

33. Zheng, S. Studies on Chem i cal Con stit u ents of Salix matsudana. 437–441 (2002).

34. Li, X. *et al.* Isolation and characterization of phenolic compounds from the leaves of Salix matsudana. *Molecules* **13**, 1530–1537 (2008).

35. He, K. & Du, J. Two new steroidal alkaloids from the roots of Sarcococca ruscifolia. *J. Asian Nat. Prod. Res.* **12**, 233–238 (2010).

36. Zhang, P. *et al.* Pregnane alkaloids from Sarcococca ruscifolia and their cytotoxic activity. *Phytochem. Lett.* **14**, 31–34 (2015).

37. Yi, X. *et al.* Cytotoxic androstane derivatives from Sarcococca ruscifolia. *Fitoterapia* **144**, 104604 (2020).

38. Li, H. *et al.* A new bibenzyl derivative with nuclear factor-kappaB inhibitory activity from Schefflera arboricola (Araliaceae). *Nat. Prod. Res.* **29**, 1139–1144 (2015).

39. Ngoc, T. D. & Le Nguyen, T. Chemical Composition and Antimicrobial Activity of Essential Oils from the Leaves and Stems of Schefflera arboricola (Hayata) Merr. Collected in Vietnam. *J. Essent. Oil-Bearing Plants* **22**, 1401–1406 (2019).

40. Liu, H. T., Xu, L. J., Peng, Y., Yang, X. W. & Xiao, P. G. Two new lignans from Schisandra henryi. *Chem. Pharm. Bull.* **57**, 405–407 (2009).

41. Szopa, A., Barnaś, M. & Ekiert, H. Phytochemical studies and biological activity of three Chinese Schisandra species (Schisandra sphenanthera, Schisandra henryi and Schisandra rubriflora): current findings and future applications. *Phytochem. Rev.* **18**, 109–128 (2019).

42. Shang, X. *et al.* The genus Scutellaria an ethnopharmacological and phytochemical review. *J. Ethnopharmacol.* **128**, 279–313 (2010).

43. Wang, D., Huang, L. & Chen, S. Senecio scandens Buch.-Ham.: A review on its ethnopharmacology, phytochemistry, pharmacology, and toxicity. *J. Ethnopharmacol.* **149**, 1–23 (2013).

44. Leu, Y. L., Lin, C. L. & Kuo, P. C. Constituents from senecio scandens and their antioxidant bioactivity. *Arch. Pharm. Res.* **34**, 377–382 (2011).

45. Wang, C. F., Li, J. P., Zhang, Y. B. & Zhang, Z. Z. Chemical constituents from the roots of senecio scandens. *Chem. Nat. Compd.* **47**, 243–245 (2011).

46. Manikandaselvi, S., Vadivel, V. & Brindha, P. Studies on physicochemical and nutritional properties of aerial parts of Cassia occidentalis L. *J. Food Drug Anal.* **24**, 508–515 (2016).

47. Yadav, J. P. *et al.* Fitoterapia Cassia occidentalis L .: A review on its ethnobotany , phytochemical and pharmacological pro fi le. *Fitoterapia* **81**, 223–230 (2010).

48. FO, A., ZS, O. & CV, N. Phytochemical and Antibacterial Potentials of Senna tora Leaf and Seed Extracts against Some Clinically Isolated Bacteria. *J. Bacteriol. Parasitol.* **09**, 3–6 (2018).

49. Pawar, H. A. & Lalitha, K. G. Extraction, Characterization, and Molecular Weight Determination of Senna tora (L.) Seed Polysaccharide. *Int. J. Biomater.* **2015**, (2015).

50. Sarwa, K. Phytochemical and Biological Potential of Cassia tora Linn. *European J. Med. Plants* **4**, 946–963 (2014).

51. Zhang, Y. *et al.* Chemical components and antioxidant activity of the volatile oil from Cassia tora L. seed prepared by supercritical fluid extraction. *J. Food Lipids* **14**, 411–423 (2007).

52. Karou, S. D. *et al.* Sida acuta Burm. f.: A medicinal plant with numerous potencies. *African J. Biotechnol.* **6**, 2953–2959 (2007).

53. Prakash, A., Varma, R. K. & Ghosal, S. Alkaloidal constituents of Sida acuta S. humilis, S. rhombifolia and S. spinosa. *Planta Med.* **43**, 384–388 (1981).

54. Abat, J. K., Kumar, S. & Mohanty, A. Ethnomedicinal, Phytochemical and

Ethnopharmacological Aspects of Four Medicinal Plants of Malvaceae Used in Indian Traditional Medicines: A Review. *Medicines* **4**, 75 (2017).

55. Semwal, D. K. *et al.* The genus Stephania (Menispermaceae): Chemical and pharmacological perspectives. *J. Ethnopharmacol.* **132**, 369–383 (2010).

56. Yang, L., Lin, J., Zhou, B., Liu, Y. & Zhu, B. Activity of compounds from Taxillus sutchuenensis as inhibitors of HCV NS3 serine protease. **6419**, (2016).

57. Liu, D., Ju, J. & Yang, J. Studies on chemical constituents from Tetrastigma hypoglaucum. 4–6 (2003).

58. Yan, H. G. *et al.* Determination of phenanthrenes and stilbenoid in the ethyl acetate extract of: Thunia alba (Lindl) by HPLC-DAD. *Anal. Methods* **8**, 4867–4871 (2016).

59. Majumder, P. L., Roychowdhury, M. & Chakraborty, S. Thunalbene, a stilbene derivative from the orchid Thunia alba. *Phytochemistry* **49**, 2375–2378 (1998).

60. Zhao, H. H., He, J. T., Liu, Z. X. & Huang, J. G. Cytotoxicity of chemical constituents from Torricellia tiliifolia DC. on Spodoptera litura (SL-1) cells. *Pestic. Biochem. Physiol.* **144**, 19–26 (2018).

61. Menegazzo, R. F. *et al.* Chemical composition of Tradescantia pallida (Rose) D.R. Hunt var. purpurea Boom (Commelinaceae) essential oil. *Nat. Prod. Res.* **0**, 1–5 (2020).

62. Hamed, M. M., Mohamed, M. A. & Ibrahim, M. T. Cytotoxic Activity Assesment of Secondary Metabolites from Tecomaria capensis v . aurea. **8**, 1173–1182 (2016).

Serial no	Plant name	Chemical name	Pubchem CID
01	*Smilax glabra* Roxb	Astilbin[1]	119258
		Neoastilbin[1]	442437
		Taxifolin[1]	439533
		-epicatechin[1]	72276
		Isoastilbin[1]	316844
		Isoengelitin[1]	101937309
		Quercetin[1]	5280343
		Naringenin[1]	932
		Kukulkanin B[1]	6439187
		Sinensin[1]	10456395
		Cinchonain Ia[1]	10456516
		Apigenin[1]	5280443
		Myricetin[1]	5281672
		4,4',6-trihydroxyauron[1]	11623165
		Smilachromanone[1]	102132638
		Sakuranetin[1]	73571
		Arthromerin B[1]	44257058
		(2S,3S)-glucodistylin[1]	3035567
		Cinchonain Ib[1]	442675
		Luteolin[1]	5280445
		Aureusidin[1]	5281220
		Syringic acid[1]	10742

		2-methyl-succinic acid[1]	10349
		Ferulic acid[1]	445858
		Lignoceric acid[1]	11197
		5-O-caffeoylshikimic acid [1]	5281762
		β-sitosterol[1]	222284
		Stigmasterol[1]	5280794
		Daucosterol[1]	5742590
		Smilagenin[1]	91439
		Diosgenin[1]	99474
		-secoisolariciresinol[1]	65373
		4-ketopinoresinol[1]	44578390
		Kompasinol A[1]	102505446
		Aiphanol[1]	10366595
		Trans-caffeic acid[1]	2518
		Juncusyl ester B[1]	101688442
		Methylcaffeate[1]	689075
		Piceatannol[1]	667639
		Smiglastilbene[1]	102132636
		Smiglabrone A[1]	102132637
		Vanillin[1]	1183
		p-hydroxy-benzaldehyde[1]	126
		Acetovanillone[1]	2214

		-scytalone[1]	3477029
		Glucosyringic acid[1]	10383888
		Protocatechuic acid[1]	72
		3-methoxygallic acid[1]	54690297
		Hydroxytyrosol[1]	82755
		Trans-piceid[1]	5281718
		Heptanoic acid,isopropyl ester[1]	520710
		Pentanoic acid,propyl ester[1]	67328
		Butanedioic acid,diethyl ester[1]	11974586
		Caproic acid,methyl ester[1]	7824
		Hexanedioic acid,ethyl methyl ester[1]	573721
		Pentanoic acid4-oxo-,butyl ester[1]	16331
		Nonanoic acid,ethyl ester[1]	22676887
		Dimethyl suberate[1]	15611
		Dimethyl azelaate[1]	15612
		Dimethyl pimelate[1]	74416
		3-methyldodecane[1]	28469
		Butanedioic acid,dibutyl ester[1]	8830
		Pentanedioic acid,dibutyl ester[1]	81101
		Dodecanoic acid,ethyl ester[1]	7800
		Butanoic acid,octyl ester[1]	61030
		Hexadecane[1]	11006
		Tetradecanoic acid, ethyl ester[1]	31283

		2,4-dimethyl-3-hexanone[1]	86770
		Pentadecanoic acid, methyl ester[1]	23518
		Decanedioic acid,diethyl ester[1]	8049
		Pentadecanoic acid,ethyl ester[1]	38762
		Hexadecenoic acid[1]	5282743
		Heptadecanoic acid[1]	10465
		Hexadecanoic acid,ethyl ester[1]	12366
		Eicosane[1]	8222
		Heptadecanoic acid, methyl ester[1]	15609
		7-octadecenoic acid, methyl ester[1]	5364440
		16-octadecenoic acid,methyl ester[1]	5364513
		Octadecanoic acid,methyl ester[1]	8201
		9-octadecenoic acid,ethyl ester[1]	5364430
		Octadecanoic acid-2methyl,methyl ester[1]	102828
		Octadecanoic acid,ethyl ester[1]	8122
		Nonane[1]	8141
		L-linalool[1]	443158
		1-terpineol[1]	11468
		L-borneol[1]	1201518
		Terpinen-4-ol[1]	11230
		α-Terpineol[1]	442501

		(E,E)-2,4-decadienal[1]	5283349
		α-cedrol[1]	65575
		β-eudesmol[1]	91457
		Myristic acid[1]	11005
		Methyl palmitate[1]	8181
		Methyl linolenate[1]	5319706
		Linoleic acid[1]	5280450
		Stearic acid[1]	5281
		Docosane[1]	12405
		Tricosane[1]	12534
		5-hydroxy methyl furfural[1]	237332
		Nicotinamide[1]	936
		5-hydroxymaltol[1]	70627
		5-hydroxyuridine[1]	94196
		Galactose[1]	6036
		Rhamnose[1]	25310
		syringaresinol[2]	100067
		lasiodiplodin[2]	14562696
		de-O-methyllasiodiplodin[2]	14562693
		lyoniresinol[2]	11711453
		trans-resveratrol[2]	445154

		dihydrokaempferol[2]	122850
02	*Solanum nigrum* Linn	b-Pinene[3]	14896
		1(7)-p-Menthene[3]	517973
		Limonene[3]	22311
		a-Pinene[3]	6654
		2-Methylisoborneol[3]	16913
		a-Phellandrene[3]	7460
		para-Cymene[3]	7463
		Lauric acid[3]	3893
		c-Eudesmol[3]	6432005
		a-Cadinol[3]	10398656
		Linalyl butanoate[3]	62321
		Dillapiole[3]	10231
		Laurenene[3]	13818582
		5-Epi-Paradisol[3]	15560332
		Phytol[3]	5280435
		glycine[4]	750
		proline[4]	145742
		solasodine[4]	442985
		Solamargine[4]	73611

		Solasonine[4]	119247
		tigogenin[4]	99516
		solanigroside C[4]	16083118
		solanigroside D[4]	16083121
		solanigroside E[4]	16083119
		solanigroside F[4]	16083122
		solanigroside G[4]	16083120
		solanigroside H[4]	16083123
		degalactotigonin[4]	162401
		gallic acid[5]	370
		protocatechuic acid[5]	72
		epigallocatechin[5]	72277
		chlorogenic acid[5]	1794427
		gentisic acid[5]	3469
		vanillic acid[5]	8468
		caffeic acid[5]	689043
		syringic acid[5]	10742
		epicatechin[5]	72276
		Epigallocatechin gallate[5]	65064
		gallocatechin gallate[5]	5276890
		p-coumaric acid[5]	637542

		ferulic acid[5]	445858
		rutin[5]	5280805
		m-coumaric acid[5]	637541
		luteolin[5]	5280445
		myricetin[5]	5281672
		quercetin[5]	5280343
		apigenin[5]	5280443
		kaempferol[5]	5280863
		hesperetin[5]	72281
		adenosine[6]	60961
		uttroside B[6]	44566638
		diosgenin[6]	99474
03	*Solena amplexicaulis* (Lam.) Gandhi	Hexahydropyridine, 1-methyl-4-[4,5dihydroxyphenyl][7]	619267
		1-Octanamine[7]	8143
		1-Tetradecanamine[7]	16217
		Carane[7]	79043
		Pentane-2,4-dione, 3-(1-adamantyl)[7]	584545
		Phytol[7]	5280435
		1,3-Cyclopentanedione[7]	77466
		Undecane[7]	14257

		1,2,4-Triazino[5,6-E] [1,2,4]-triazine-3,6-dione, hexahydro[7]	536727
		4-Hydroxyphenyl 3-nitrobenzoate[7]	541526
		Taurolidine[7]	29566
		4-(4-Ethoxyphenyl) but-3-en-2-one[7]	6143289
		Trehalose[7]	7427
		9-Tetradecen-1-ol,acetate[7]	5363249
		Hexadecanoic acid, methyl ester[7]	8181
		1-Methyl-3 ethyladamantane[7]	519330
		9-Octadecenoic acid (Z)-, methyl ester[7]	5364509
		Heptadecanoic acid, 10-methyl-,methyl ester[7]	554139
		Dodecanoic acid[7]	3893
		Tetradecanoic acid[7]	11005
		1,2-Benzenedicarboxylic acid,bis(2-methylpropyl) ester[7]	6782
		Pentadecanoic acid, 14-methyl-,methyl ester[7]	21205
		n-Hexadecanoic acid[7]	985
		10,13-Octadecadienoic acid, methyl ester[7]	5365678
		trans-13-Octadecenoic acid, methyl ester[7]	543418
		9,17-Octadecadienal, (Z)-[7]	5365667

		Phthalicacid, di(2-propylpentyl)ester[7]	191964
04	*Sophora japonica* Linn	Kaempferol[8]	5280863
		Kaempferol 3,7-diglucoside[8]	6325460
		Kaempferol-3-O-β-D-sophoroside[8]	5282155
		Kaempferol 3-O- robinobioside[8]	15944778
		Quercetin[8]	5280343
		Tamarixetin (4′-O-methyl quercetin)[8]	5281699
		Isoquercitrin[8]	5280804
		Quercitrin[8]	5280459
		Rutin[8]	5280805
		Isorhamnetin[8]	5281654
		Narcissin[8]	5481663
		Apigenin[8]	5280443
		Isoscutellarein[8]	5281665
		Genistein[8]	5280961
		Genistin[8]	5281377
		Sophoricoside[8]	5321398
		Sophorabioside[8]	11968944

		Prunetin[8]	5281804
		Daidzin[8]	107971
		Daidzein[8]	5281708
		Di-O-methyldaidzein[8]	136419
		Biochanin A[8]	5280373
		Sissotrin[8]	5280781
		Formononetin[8]	5280378
		Afrormosin[8]	5281704
		Ononin[8]	442813
		Glycitin[8]	187808
		Tectoridin[8]	5281810
		Irisolidone[8]	5281781
		Pseudobaptigenin[8]	5281805
		Calycosin[8]	5280448
		Cajanin[8]	5281706
		Pratensein[8]	5281803
		Orobol[8]	5281801
		7, 3’-di-O-methylorobol[8]	13845970

		5-hydroxypseudobaptigenin-7-O-glucoside[8]	44257299
		calycosin-7-O-glucoside[8]	5318267
		Dihydroformononetin[8]	14730796
		Sophorol[8]	6452639
		Betulin[8]	72326
		Sophoradiol[8]	9846221
		Kakkasaponin II[8]	102500447
		Kaikasaponin I[8]	102120183
		Kaikasaponin III[8]	188384
		Soyasaponin I[8]	122097
		Dehydrosoyasaponin I[8]	656760
		Phaseoside IV[8]	101679107
		Soyasaponin III[8]	21607811
		Azukisaponin I[8]	14103656
		Azukisaponin II[8]	13326387
		Kaikasaponin II[8]	101538997
		Soyasapogenol B[8]	115012
		Matrine[8]	91466
		Sophocarpine[8]	115269
		N-methylcytisine[8]	670971

		Cytisine[8]	10235
		Puerol A[8]	14691941
		Puerol B[8]	56776306
		Maltol[8]	8369
		Soyamaloside A[8]	122209598
		Soyamaloside B[8]	122209599
		Soyamaloside C[8]	44715841
		Maackiain[8]	91510
		Sophojaponicin[8]	6326060
		Trifolirhizin[8]	442827
		Anhydropisatin[8]	11822566
		Medicagol[8]	5319322
		Medicarpin[8]	336327
		β-sitosterol[8]	222284
		Stigmasterol[8]	5280794
		Daucosterol[8]	5742590
		Octacosanol[8]	68406
		Eicosanol[8]	12404
		Hexacosanic acid[8]	10469
		Behenic acid[8]	8215

		Pyrocatechol[8]	289
		Protocatechuic acid[8]	72
		Gallic acid[8]	370
		1,6-di-O-galloyl-β-D-glucose[8]	91227631
		N, N'-diferuloyl-putrescine[8]	5321825
		N-p-coumaroyl-N'-feruloylputrescine[8]	44241259
		N, N'-dicoumaroylputrescine[8]	44241258
05	*Stemona tuberosa* Lour	tuberostemonine K[9]	11552547
		tuberospironine[9]	11537361
		tuberostemonine[9]	100781
		neotuberostemonine[9]	11667940
		croomine[9]	3085457
		stemoninine[9]	15983991
		Mannose[10]	18950
		Glucose[10]	5793
		Sucrose[10]	5988
		Maltose[10]	6255
		Octanoic acid[10]	379
		Nonanoic acid[10]	8158

		Decanoic acid[10]	2969
		Hendecanoic acid[10]	8180
		Lauric acid[10]	3893
		12-tridecenoic acid[10]	5282737
		Tridecanoic acid[10]	12530
		Myristoleic acid[10]	5281119
		Myristic acid[10]	11005
		Pentadecanoic acid[10]	13849
		Palmitoleic acid[10]	445638
		Palmitic acid[10]	985
		Heptadecanoic acid[10]	10465
		Linoleic acid[10]	5280450
		Oleic acid[10]	445639
		Stearic acid[10]	5281
		Nonadecanoic acid[10]	12591
		8-Eicosenoic acid[10]	15708976
		Arachidic acid[10]	10467
		Heneicosanoic acid[10]	16898
		Erucic acid[10]	5281116
		Behenic acid[10]	633932

06	*Stephania tetrandra* S. Moore	N-methylcoclaurine[11]	440595
		Juziphine[11]	14526072
		Coclaurine[11]	160487
		Protosinomenine[11]	102067840
		Reticuline[11]	439653
		Oblongine[11]	173713
		Fangchinoline[11]	73481
		Cycleanorine[11]	3082494
		Cycleanine[11]	121313
		Stephibaberine[11]	9938773
		Cepharanthine[11]	10206
		Obaberine[11]	100231
		Isotetrandrine[11]	457825
		2-Norcepharanthine[11]	181384
		Fenfangjine A[11]	102121497
		Fenfangjine B[11]	102121498
		Fenfangjine C[11]	102121829
		Dicentrine[11]	101300
		Tazopsine[11]	11653181
		Oxonantenine[11]	3084224
		Cassameridine[11]	12302502
		Nantenine[11]	197001

		Cassythicine[11]	442194
		Isoboldine[11]	133323
		Corytuberine[11]	160500
		Dehydrocrebanine[11]	149600
		Dehydrodicentrine[11]	3084326
		Roemerine[11]	119204
		Fenfangjine G[11]	102004748
		Stepharanine[11]	10358881
		Dehydrodiscretamine[11]	102316663
		Cyclanoline chloride[11]	28564
		Stepholidine[11]	6917970
		Discretamine[11]	22955
		Isoscoulerine[11]	188442
		Corydalmine[11]	161665
		Tetrahydrojatrorrhizine[11]	185605
		Tetrahydropalmatine[11]	5417
		Capaurine[11]	94149
		Cyclanoline[11]	3082134

		Stephenanthrine[11]	161379
		Argentinine[11]	10085878
		Fenfangjine F[11]	10781192
		b-sitosterol[11]	222284
		Stigmasterol[11]	5280794
		Oxofangchirine[12]	128108
		Cycleahomine[12]	102121496
		-curine[12]	253793
		-Dicentrine[12]	630859
		Isocorydine[12]	10143
		Crebanine[12]	333544
		Oxocrebanine[12]	3084713
07	*Strobilanthes cusia* (Nees) J.B.Imlay	indirubin[13]	10177
		indigotin[13]	10215
		4(3H)-quinazolinone[13]	135408753
		indican[13]	441564
		1H-indole-3-carboxylic acid[13]	69867
		2-benzoxazolinone[13]	6043
		2-hydroxy-1,4-benzoxazin-3-one[13]	322636

		baphicacanthin A[13]	130475803
		baphicacanthin B[13]	130475831
		Verbasoside[13]	11754080
		-syringaresinol[13]	332426
		-episyringaresinol[13]	45482321
		Eleutheroside E[13]	71312557
		Acteoside[13]	5281800
		adenosine[13]	60961
		Uridine[13]	6029
		Isoacteoside[13]	6476333
		Martynoside[13]	5319292
		Isomartynoside[13]	91895373
		stigmasta-5,22-diene-3β,7β-diol[13]	6442694
		β-sitosterol[13]	222284
		lupenone[13]	92158
		lupeol[13]	259846
		betulin[13]	72326
		2,3-dihydroxy-12-oleanen-28-oic acid[13]	54067425
		4-hydroxy-3-methoxybenzoic acid[13]	8468
		lauric acid[13]	3893

		11,12-dihydroxy-7,9-octadecadienoic acid[13]	129881881
		sucrose[13]	5988
		Chrysophanol[13]	10208
		monobutyl phthalate[13]	8575
		dibutyl phthalate[13]	3026
		Nicotinamide[13]	936
		Squalene[13]	638072
08	*Swertia bimaculata*(Sieb.et Zucc.)Hook.f.et Thoms	decussatin(1-hydroxy-3,7,8-trimethoxyxanthone)[14]	5378284
		corymbiferin[14]	71437983
		1-hydroxy-2,3,4,7-tetramethoxyxanthone[14]	5318358
		2-hydroxy-1,3,4,7-tetramethoxyxanthone[14]	101304457
		norswertianolin[14]	5281659
		swertianolin[14]	5281662
		Bellidifolin[15]	5281623
		Oleanolic acid[15]	10494
		Coniferaldehyde[15]	5280536
		Sinapaldehyde[15]	5280802

		5-(3′′ -Hydroxypropyl)-7-methoxy-2-(3′ ,4′-methylenedioxyphenyl)benzofuran[15]	485186
		5-Carbethoxyethenyl-7-methoxy-2-(3,4-methylenedioxyphenyl)benzofuran[15]	134814038
		3-Acetoxymethyl-5-[(E)-2-formylethen-1-yl]-2-(4-hydroxy-3-methoxyphenyl)-7-methoxy-2,3-dihydrobenzofuran[15]	75202444
		Simulanol[15]	22297418
		2,6,2',6'-tetramethoxy-4,4'-bis(2,3-epoxy-1-hydroxypropyl)biphenyl[15]	85350942
		Buddlenol A [15]	50909267
		-8-Hydroxypinoresinol[15]	3010930
		(+)-(7R,7'R,7''R,7'''S,8S,8'S,8''S,8'''S)-4'',4'''-dihydroxy-3,3',3'',3''',5,5'-hexamethoxy-7,9':7',9-diepoxy-4,8'':4',8'''-bisoxy-8,8'-dineolignan-7'',7''',9'',9'''-tetraol[15]	56673402
		(-)-(7R,7'R,7''R,8S,8'S,8''S)-4',4''-dihydroxy-3,3',3'',5-tetramethoxy-7,9':7',9-diepoxy-4,8''-oxy-8,8'-sesquineolignan-7'',9''-diol[15]	56680033
		(+)-(7R,7'R,7''R,7'''R,8S,8'S,8''S,8'''S)-4'',4'''-dihydroxy-	56659551

		3,3',3'',3''',5,5'-hexamethoxy-7,9':7',9-diepoxy-4,8'':4',8'''-bisoxy-8,8'-dineolignan-7'',7''',9'',9'''-tetraol[15]	
09	*Symplocos paniculata* (Thunb.) Miq	Ursolic acid[16]	64945
		corosolic acid[16]	6918774
		2α, 3α, 19α, 23-tetrahydroxyurs-12-ene-28-oic acid[16]	182497
		Oleic acid[16]	445639
		linoleic acid[16]	5280450
		Symplocososide B[16]	101371233
		stigmasterol[16]	5280794
		lupeol[16]	259846
		salirepin D[16]	539422
		Octacos-1-ene[17]	87821
		β-Amyrin[17]	73145
		Betulin[17]	72326
		α-amyrin[17]	73170
		Betulic acid[17]	64971
		β –sitosterol[17]	222284
		Symplocoside[17]	44257110
		-epiafzelechin[17]	443639
		Loturine[17]	5281404
		Ellagic acid[17]	5281855

		Salireposide[17]	117440
10	*Taraxacum mongolicum* Hand.-Mazz	apigenin[18]	5280443
		luteolin[18]	5280445
		quercetin[18]	5280343
		luteolin-7-β-D-glucopyranoside[18]	5280637
		gynuramide II[18]	76312320
		1-linoleylglycerol[18]	5283469
		gingerglycolipid B[18]	10009754
		gingerglycolipid A[18]	10349562
		taraxasterol[18]	115250
		β-sitosterol[18]	222284
		stigmasterol[18]	5280794
		arnidiol[19]	10478550
		psi -taraxasterol[19]	5270605
		faradiol[19]	9846222
		taraxacin[19]	5241825
		taraxinic acid[19]	9921439
		β-amyrin[19]	73145
		Alpha- amyrin[19]	73170
		hesperetin[19]	72281
		artemetin[19]	5320351
		isoetin[19]	5281649

		genkwanin[19]	5281617
		Caffeic acid[19]	689043
		chlorogenic acid[19]	1794427
		3,5-di-O-caffeoylquinic acid[19]	6474310
		ferulic acid[19]	445858
		cichoric acid[19]	5281764
		4,5-di-O-caffeoylquinic acid[19]	6474309
		p-hydroxybenzoic acid[19]	135
		p-coumaric acid[19]	637542
		syringic acid[19]	10742
		gallic acid[19]	370
		caffeic acid ethyl ester[19]	5317238
11	*Taxus wallichiana* Zucc	n-Eicosane[20]	8222
		docosane[20]	12405
		n-pentacosane[20]	12406
		Santolina triene[20]	519872
		1-Hepten-3-ol[20]	21057
		2-hexenol[20]	5318042
		n-heptan-2-ol[20]	10976
		n-heptanol[20]	8129

		2-octen-1-ol[20]	5318599
		1-octanol[20]	957
		-verbenol[20]	61126
		myrtenol[20]	10582
		geraniol[20]	637566
		eugenol[20]	3314
		globulol[20]	12304985
		n-Heptanal[20]	8130
		benzaldehyde[20]	240
		n-octanal[20]	454
		-2-octenal[20]	5283324
		2-nonenal[20]	5283335
		dodecanal[20]	8194
		anisaldehyde[20]	31244
		Hexanoic acid[20]	8892
		benzoic acid[20]	243
		(Z)-3-hexenyl acetate[20]	5363388
		methyl benzoate[20]	7150
		octyl formate[20]	8176

		benzyl acetate[20]	8785
		methyl salicylate[20]	4133
		n-octyl acetate[20]	8164
		sabinyl acetate[20]	94266
		(E)-2-hexenyl-n-hexanoate[20]	5352973
		anisyl acetate[20]	7695
		(Z)-3-hexenyl benzoate[20]	5367706
		geranyl tiglate[20]	5367785
		n-amyl anisoate[20]	243793
		geranyl-n-heptanoate[20]	6450452
		β-Caryophyllene[20]	5281515
		α-humulenen[20]	6508206
		caryophyllene oxide[20]	1742210
		(E,E)farnesol[20]	445070
		(Z)-sabinene hydrate[20]	62367
		Taxol[20]	36314
		Baccatin III[20]	65366
		1β-Hydroxybaccatin I[20]	146158535
		10-Deacetylcephalomannine[20]	6440548
		10-Deacetyltaxol[20]	155831

		19-Hydroxybaccatin III[20]	5318151
		10-Deacetyl-7-xylosyltaxol[20]	24791040
		10-Deacetyl-7-Xylosyltaxol C[20]	24793433
		Cephalomannine[20]	6436208
		1-Hydroxy-2-deacetoxytaxinine J[20]	5321656
		2′-Deacetoxyaustrospicatine[20]	14446178
		7,2′-Bisdeacetoxyaustrospicatine[20]	15226622
		Taxacustin[20]	102115826
		Taxayuntin[20]	101916323
		7,9-Dideacetyltaxayuntin[20]	101711017
		Tasumatrol B[20]	10962444
		Taxamairin F[20]	76327123
		Taxusabietane A[20]	76330776
		10-Deacetylbaccatin III[20]	154272
		Brevifoliol[20]	178222
		2-Acetoxybrevifoliol[20]	100991413
		Wallifoliol[20]	21673419
		19-Debenzoyl-19-acetyltaxinine M[20]	101926854
		5-Deacetyl-1-hydroxybaccatin I[20]	14707724
		2-Deacetoxytaxinine B[20]	6442229
		7-O-acetyltaxine A[20]	101117774

		Taxusin[20]	167825
		13-Acetyl-13-decinnamoyltaxchinin B[20]	10439805
		7-Xylosyl-10-deacetyltaxol C[20]	15222314
		Baccatin IV[20]	15275710
		α-conidendrin[20]	457194
		β-conidendrin[20]	12303845
		hydroxymatairesinol[20]	10948757
		isoliovil[20]	44147426
		(–)-secoisolariciresinol[20]	65373
		β-sitosterol[20]	222284
		daucosterol[20]	5742590
		ponasterone[20]	115127
		ecdysone[20]	19212
		amentoflavone[20]	5281600
		sciadopitysin[20]	5281696
12	*Thalictrum foliolosum* DC	Thalrugosidine[21]	5321920
		thalrugosaminine[21]	5321919
		thalisopine[21]	179390
		thalirugidine[21]	44584027
		thalirugine[21]	44584030

		8-oxyberberine[21]	11066
		noroxyhydrastinine[21]	89047
		thalicarpine[21]	21470
		thalidasine[21]	159795
		Berberine[21]	2353
		jatrorrhizine[21]	72323
		palmatine[21]	19009
		columbamine[21]	72310
		dehydrodiscretamine[21]	102316663
		rugosinone[21]	442350
		xanthoplanine[21]	14262868
		reticuline[22]	439653
13	*Tinospora sagittata* (Oliv.) Gagnep	Tinophylloloside[23]	15694364
		Tinospinoside C[23]	56951689
		Tinospinoside D[23]	71473354
		Columbin[23]	188289
		Fibleucin[23]	56678199
		Isocolumbin[23]	24721165
		Tinoside[23]	184514
		6-Hydroxycolumbin[23]	101607218

		Tinospinoside E[23]	71473390
		Tinospin E[23]	71473355
		Tinosagittone A[23]	101862378
		Tinosagittone B[23]	101862379
		Jatrorrhizine[23]	72323
		Demethyleneberberine[23]	363209
		Columbamine[23]	72310
		Stepharanine[23]	10358881
		Dehydrodiscretamine[23]	102316663
		Dehydrocorydalmine[23]	3083983
		Magnoflorine[23]	73337
		Menisperine[23]	161487
		Tembetarine[23]	167718
		Reticuline[23]	439653
		2-Deoxy-20β-hydroxyecdysone[23]	9912297
14	*Toona sinensis* (A. Juss.) Roem.	Ursolic acid[24]	64945
		Betulonic acid[24]	122844
		Gedunin[24]	12004512
		7-Deacetylgedunin[24]	3034112
		Proceranone[24]	101277340
		Grandifoliolenone[24]	101289764

		Bourjotinolone A[24]	21603611
		Piscidinol A[24]	12004524
		Hispidol B[24]	13967183
		Ocotillone[24]	12313665
		Methyl shoreate[24]	44421646
		Shoreic acid[24]	12315515
		Richenone[24]	101599479
		Cabralealactone[24]	44421647
		Cylindrictone D[24]	25104959
		Hollongdione[24]	15559638
		Bourjotinolone B[24]	101280173
		Sapellin E acetate[24]	73348891
		Azadirone[24]	10906239
		Toosendanin[24]	9851101
		Phytol[24]	5280435
		Matairesinol[24]	119205
		Lyoniresinol[24]	11711453
		Scopoletin[24]	5280460
		4, 7-Dimethoxy-5-methylcoumarin[24]	185740
		(+)-Catechin[24]	9064
		(−)-Epicatechin[24]	72276
		Procyanidin B3[24]	146798

		Procyanidin B4[24]	147299
		Quercetin[24]	5280343
		Quercitrin[24]	5280459
		Isoquercitrin[24]	5280804
		Rutin[24]	5280805
		Kaempferol[24]	5280863
		Astragalin[24]	5282102
		Myricetin[24]	5281672
		Myricitrin[24]	5281673
		Loropetalin D[24]	44259006
		5,7-Dihydroxy-8-methoxy flavone[24]	5281703
		Gallic acid[24]	370
		Methyl gallate[24]	7428
		Ethyl gallate[24]	13250
		Syringic acid[24]	10742
		Aloeemodin[24]	10207
		Daucosterol[24]	5742590
		beta-Sitosterol[24]	222284
		Adenosine[24]	60961
		Methylthiirane[25]	21766881
		Isogeraniol[25]	5362876
		(-)-cis-Sabinol[25]	42626427
		α-Limonene diepoxide[25]	232703

		Isopinocarveol[25]	102667
		2-Isopropylidene-3-methylhexa-3,5-dienal[25]	5368460
		β-Cyclocitral[25]	9895
		Z,Z,Z-4,6,9-Nonadecatriene[25]	5362863
		Cyclohexene,1-(2-nitro-2-propenyl)-[25]	561668
		p-Eugenol[25]	3314
		α-Cubebene[25]	86609
		α-Copaene[25]	19725
		β-Bourbonene[25]	62566
		α-Guaiene[25]	5317844
		Humulen-(v1)[25]	5362885
		β-Caryophyllene[25]	1742210
		Isoledene[25]	530426
		Aristolene[25]	530421
		β-Guaiene[25]	15560252
		Seychellene[25]	519743
		(+)-Aromadendrene[25]	11095734
		Curcumene[25]	92139
		Longifolene-(V4)-[25]	570529
		Himachala-2,4-diene[25]	583791
		α-Himachalene[25]	11830551

		Cadina-1,3,5-triene[25]	6429077
		δ-Cadinene[25]	441005
		Cadina-1,4-diene[25]	6427091
		Cadala-1(10),3,8-triene[25]	593889
		Eudesma-3,7(11)-diene[25]	6432648
		(-)-Spathulenol[25]	13854255
		Cedren-13-ol, 8-[25]	519545
		Aromadendrene oxide-(1)[25]	528759
		α-Copaen-11-ol[25]	101281096
		Tetracyclo[6.3.2.0(2,5).0(1,8)]tridecan-9-ol, 4,4-dimethyl[25]	585744
		Cubenol[25]	519857
		tau -Cadinol[25]	12302222
		Tricyclo[5.2.2.0(1,6)]undecan-3-ol, 2-methylene-6,8,8-trimethyl[25]	535346
		Eudesm-4(14)-en-11-ol[25]	91457
		Ledene oxide-(II)[25]	534497
		δ-Cadinol[25]	6431456
		Murolan-3,9(11)-diene-10-peroxy[25]	536595
		2,2,6-Trimethyl-1-[(1E)-3-methyl-1,3-butadienyl] -5-methylene-7-oxabicyclo[4.1.0]heptane[25]	5363734
		Cycloisolongifolene, 8,9-dehydro[25]	594593
		Androst-2,16-diene[25]	617873

		Menthol, 1'-(butyn-3-one-1-yl)-,(1S,2S,5R)-[25]	536442
		5,8,11,14,17-Eicosapentaenoic acid, methyl ester, (all-Z)[25]	6421261
		Androst-5,7-dien-3-ol-17-one[25]	276591
		Strophanthidol[25]	258412
		l-(+)-Ascorbic acid 2,6-dihexadecanoate[25]	54722209
		9,12-Octadecadienoic acid, methyl ester, (E,E)-[25]	5362793
		2-Hydroxy-1-(hydroxymethyl)ethyl (9E,12E,15E)-9,12,15-octadecatrienoate[25]	5367459
		6,9,12,15-Docosatetraenoic acid, methyl ester[25]	5362672
		1,1-Dimethyltetradecyl hydrosulfide[25]	545889
		2-(7-Heptadecynyloxy)tetrahydro-2H-pyran[25]	543312
		Heptadecane, 9-octyl-[25]	292286
		Corynan-17-ol,18,19-didehydro-10-methoxy-, acetate ester[25]	550058
		1,7-Dicyclopentyl-4-n-octylheptane[25]	294711
		Heptadecane, 9-hexyl[25]	296566
		Tetracosane, 11-decyl-[25]	294707
		17-Pentatriacontene[25]	5365022
		3-Ethyl-5-(2'-ethylbutyl)octadecane[25]	292285

		Propanoic acid, 2-(3-acetoxy-4,4,14-trimethylandrost-8-en-17-yl)-[25]	631957
15	*Trachycarpus fortunei* (Hook.) H. Wendl	Styrene[26]	7501
		Trimeprazine[26]	5574
		Evocarpine[26]	5317303
		Magnoshinin[26]	442896
		Voacamine[26]	11953931
		Protoveratrine A[26]	8931
		Stearamide[26]	31292
		1-(4-Fluorophenyl)-2-(methylthio)-1H-imidazole-5-carboxylic acid[26]	2113884
		Phosphatidylethanolamine alkenyl[26]	134733228
		Umbelliferone[26]	5281426
		Buddledin A[26]	5281514
		Pentamidine[26]	4735
		Istamycin C1[26]	46174030
		Alpha-Oxo-benzeneacetic acid[26]	11915
		Monodechloroaminopyrrolnitrin[26]	194654
		Alpha-Amylcinnamaldehyde[26]	31209
		Podocarpic acid[26]	93017
		Stearidonic acid[26]	5312508
		Allogibberic acid[26]	442001
		Abacavir[26]	441300

		(9Z)-(13S)-12,13-Epoxyoctadeca-9,11-dienoic acid[26]	5280703
		(9Z,12Z)-(8R)-Hydroxyoctadeca-9,12-dienoic acid[26]	5281117
		Timolol[26]	33624
		Dibenzo[h,rst]pentaphene[26]	9121
		Aspidospermine[26]	227613
16	*Tremella fuciformis*	9,19-Cyclolanost-24-en-3-ol, (3.beta.)[27]	91746587
		Lanosterol[27]	246983
		Acetic acid,7-Isopropenyl-1,4adimethyl-3-oxo-2,3,4,4a,5,6,7,8octahydronaphthalen-2-yl ester[27]	540645
		2(1H)Naphthalenone, 3,5,6,7,8,8a-hexahydro4,8a-dimethyl-6-(1-methylethenyl)-[27]	612605
		Lupeol[27]	259846
17	*Tripterygium wilfordii* Hook. f.	Wilfortrine[28]	73321
		wilfordine[28]	442556
		wilforgine[28]	14108469
		wilforine[28]	601100
		wilfori acids E[29]	15459518
		cangoronine[29]	101616678
		orthosphenic acid[29]	20056194
		wilforic acid B[29]	101707493

		celastrol[29]	122724
		triptocallic acid D[29]	44575705
		3-epikatonic acid[29]	10434225
		abruslactone A[29]	44575701
		dulcioic acid[29]	101051955
		regelindiol A[29]	129521
		hypodiol[29]	101936045
		regelin D[30]	129520
		regelindiol B[30]	134697
		euonine[30]	162486
		peritassine A[30]	76535542
		hypoglaunine E[30]	71717032
		hypoglaunine B[30]	44583769
		hypoglaunine A[30]	78190042
		wilfordinine A[30]	101018697
		adenosine[31]	60961
		β-sitosterol-palmitate[31]	13747834
		monopalmitin[31]	14900
		triptohairic acid[31]	100929735
		hypolide[31]	173273
		wilforlide B[31]	174362
		wilforlide A[31]	158477
		salaspermic acid[31]	44593364

		Tripterygiumine A[32]	101905231
		Tripterygiumine Q[32]	101905232
		Triptergulide A[32]	122391444
		Triptergulide B[32]	122391445
		Triptergulide C[32]	122391446
		Triptergulide D[32]	122391447
		Triptolide[33]	107985
		Tripdiolide[33]	294491
		Triptonide[33]	65411
		Triptolidenol[33]	3086461
		Tripdioltonide[33]	197582
		Tripterifordin[33]	72369
		Tripterinin[33]	129834462
		Triptophenolide methyl ether[33]	156286
		Neotriptophenolide[33]	133766
		Triptonoterpene[33]	101691231
		Triptonoterpene methyl ether[33]	15011611
18	*Tropaeolum majus* Linn	violaxanthin[34]	448438
		lutein[34]	5281243
		neoxanthin[34]	5281247
		antheraxanthin[34]	5281223
		zeaxanthin[34]	5280899
		zeinoxanthin[34]	5281234

		β–cryptoxanthin[34]	5281235
		β–caroten[34]	6384256
		α-Thujene[35]	17868
		Myrcene[35]	31253
		Benzene acetonitrile[35]	8794
		Benzyl isothiocyanate[35]	2346
		Isocaryophyllene[35]	5281522
		neochlorogenic acid[36]	5280633
		cis-3-p-CoQA[36]	92135690
		chlorogenic acid[36]	1794427
		Astragalin[37]	5282102
		Isoquercitrin[37]	5280804
		4-p-Coumaroylquinic acid[37]	5281766
		5-p-Coumaroylquinic acid[37]	164893
19	*Typhonium blumei* Nicolson et Sivadasan	Palmitic acid[38]	985
		Palmitic acid methyl ester[38]	8181
		Stearic acid[38]	5281
		Stearic acid methyl ester[38]	8201
		Undecylenic acid[38]	5634
		Undecylenic acid methyl ester[38]	8138
		cis-Vaccenic acid[38]	5282761
		cis-Vaccenic acid methyl ester[38]	5364505

		Oleic acid[38]	445639
		Oleic acid methyl ester[38]	5364509
		Petroselinic acid[38]	5281125
		Petroselinic acid methyl ester[38]	5362717
		Linoleic acid[38]	5280450
		Linoleic acid methyl ester[38]	5284421
		Conjugated (9Z,11E)-linoleic acid[38]	5280644
		α-Linolenic acid[38]	5280934
		α-Linolenic acid methyl ester[38]	5319706
		γ-Linolenic acid[38]	5280933
		cis-5,8,11,14,17-Eicosapentaenoic acid (EPA)[38]	446284
		cis-4,7,10,13,16,19-Docosahexaenoic acid (DHA)[38]	3144
		2-Hydroxystearic acid[38]	69417
		12-Hydroxystearic acid[38]	7789
		12-Hydroxystearic acid methyl ester[38]	8840
		12-Oxostearic acid methyl ester[38]	137570
		2-Hydroxy-9Z-octadecenoic acid[38]	9796304
		12R-Hydroxy-9Z-octadecenoic acid[38]	643684
		12R-Hydroxy-9E-octadecenoic acid[38]	445641
		Doxorubicin[38]	31703
20	*Uncaria laevigata* Wall. ex G. Don	uncarine D[39]	168985

		uncarine A[39]	188999
		uncarine E[39]	9885603
		ursolic acid[39]	64945
		3β,6β,19α-trihydroxyurs-12-en-28-oic acid[39]	45359677
21	*Uncaria rhynchophylla* (Miq.) Miq. ex Havil	geissoschizine methyl ether[40]	6443046
		sitsirikine[40]	3050539
		isocorynoxeine[40]	3037448
		isorhynchophylline[40]	3037048
		cadambine[40]	21723831
		corynoxeine[40]	44568160
		rhynchophylline[40]	5281408
		akuammigine[40]	1268096
		corynantheine[40]	3037997
		dihydrocorynantheine[40]	3039336
		hirsuteine[40]	3037151
		hirsutine[40]	3037884
		augustine[40]	157561
		Sucrose[41]	5988
		Chlorogenic acid[41]	1794427
		Cryptochlorogenic acid[41]	9798666
		Procyanidin b1[41]	11250133
		Catechin[41]	9064

		Epicatechin[41]	72276
		Rutin[41]	5280805
		Hyperoside[41]	5281643
		Corynoxine[41]	10475115
		strictosidine[41]	161336
		Vincosamide[41]	10163855
		Oleanolic acid[41]	10494
		corynantheidine[39]	3000341
		yohimbine[39]	8969
		angustoline[39]	3084765
		angustidine[39]	3084770
		18,19-dehydrocorynoxinic acid B[39]	24970641
		uncarinic acid C[39]	44583694
		uncarinic acid D[39]	44583695
		uncarinic acid B[39]	100967916
		trifolin[39]	5282149
		cleomiscosin B[39]	156875
		cleomiscosin D[39]	13965876
		Corynan[42]	6857493
		Vallesiachotamine[42]	5384527
		Strictosamide[42]	10345799
		Formosanan[42]	9548846
		Dihydrocadambine[42]	162138

		corynoxine B[43]	10091424
		oleanderolide[44]	11113483
		ursolic acid[44]	64945
		dibutyl phthalate[44]	3026
		loliolide[44]	100332
		salicylic acid[44]	338
		noreugenin[44]	5375252
		ferulic acid[44]	445858
		ethyl caffeate[44]	5317238
		methyl caffeate[44]	689075
22	*Urtica angustifolia* Fisch. ex Hornem.	Daucosterol[45]	5742590
		b-Sitosterol[45]	222284
		p-Coumaric acid[45]	637542
		5-Hydroxymethyl furfural[45]	237332
		Scopoletin[45]	5280460
		Gentisic acid[46]	3469
		Protocatechuic acid[46]	72
		Vanillic acid[46]	8468
		Quinic acid[46]	6508
		Ferulic acid[46]	445858
		Caffeic acid[46]	689043
		Esculetin[46]	5281416
		Secoisolariciresinol[46]	65373

		Chrysoeriol[46]	5280666
		Kaempferol[46]	5280863
		Kaempferol 3-O-glucoside[46]	5282102
		Quercitrin[46]	5280459
		Quercetin 3-O-glucoside[46]	5280804
		Quercetin 3-O-rutinoside[46]	5280805
		Isorhamnetin[46]	5281654
		Catechin[46]	9064

Reference:

1. Hua, S. *et al.* Ethnomedicine, Phytochemistry and Pharmacology of Smilax glabra: An Important Traditional Chinese Medicine. *Am. J. Chin. Med.* **46**, 261–297 (2018).
2. Lu, C. L. *et al.* Inhibitory effects of chemical compounds isolated from the rhizome of smilax glabra on nitric oxide and tumor necrosis factor-α production in lipopolysaccharide-induced RAW264.7 cell. *Evidence-based Complement. Altern. Med.* **2015**, (2015).
3. Taherpour, A. (Arman) *et al.* Chemical composition analysis of the essential oil of Solanumn nigrum L. by HS/SPME method and calculation of the biochemical coefficients of the components. *Arab. J. Chem.* **10**, S2372–S2375 (2017).
4. Mohamed Saleem, T. S. *et al.* Solanum nigrum Linn. - A review. *Pharmacogn. Rev.* **3**, 342–345 (2009).
5. Huang, H. C., Syu, K. Y. & Lin, J. K. Chemical composition of Solanum nigrum linn extract and induction of autophagy by leaf water extract and its major flavonoids in AU565 breast cancer cells. *J. Agric. Food Chem.* **58**, 8699–8708 (2010).
6. E, P. S. *et al.* Newsletter Newsletter Potawale et al . *Image (Rochester, N.Y.)* **196**, 185–196 (2008).
7. Krishnamoorthy, K. & Subramaniam, P. Phytochemical Profiling of Leaf, Stem, and Tuber Parts of Solena amplexicaulis (Lam.) Gandhi Using GC-MS . *Int. Sch. Res. Not.* **2014**, 1–13 (2014).
8. He, X. *et al.* Local and traditional uses, phytochemistry, and pharmacology of Sophora japonica L.: A review. *J. Ethnopharmacol.* **187**, 160–182 (2016).
9. Jiang, R. W. *et al.* Alkaloids and chemical diversity of Stemona tuberosa. *J. Nat. Prod.* **69**, 749–754 (2006).
10. Chen, G. *et al.* Chemical composition of diaspores of the myrmecochorous plant Stemona tuberosa

Lour. *Biochem. Syst. Ecol.* **64**, 31–37 (2016).

11. Jiang, Y., Liu, M., Liu, H. & Liu, S. *A critical review: traditional uses, phytochemistry, pharmacology and toxicology of Stephania tetrandra S. Moore (Fen Fang Ji)*. *Phytochemistry Reviews* vol. 19 (Springer Netherlands, 2020).

12. Zhang, Y. *et al.* History of uses, phytochemistry, pharmacological activities, quality control and toxicity of the root of Stephania tetrandra S. Moore: A review. *J. Ethnopharmacol.* **260**, 112995 (2020).

13. Qin, M.-Z., Liu, Y., Wu, W., Oberhänsli, T. & Wang-Müller, Q. Citation: Qin MZ (2020) The Chemical Components and Pharmacological Functions of Strobilanthes Cusia (Nees) Kuntze. *Herb Med* **6**, 1–5 (2020).

14. Li, J., Zhao, Y. L., Huang, H. Y. & Wang, Y. Z. Phytochemistry and Pharmacological Activities of the Genus Swertia (Gentianaceae): A Review. *Am. J. Chin. Med.* **45**, 667–736 (2017).

15. Dong, M. *et al.* Chemical Compounds from Swertia bimaculata. *Chem. Nat. Compd.* **54**, 964–969 (2018).

16. Chitra, S. Symplocos paniculata Miq. - A Review. *Int. J. Curr. Res. Biosci. Plant Biol.* **5**, 7–20 (2018).

17. Desf, S., Nyagrodhadi, T., Curna, K. & Curna, N. 3. literature review 3.1. 16–40 (2005).

18. Li, W., Lee, C., Kim, Y. H., Ma, J. Y. & Shim, S. H. Chemical constituents of the aerial part of Taraxacum mongolicum and their chemotaxonomic significance. *Nat. Prod. Res.* **6419**, 0 (2017).

19. Chun, H. PT. *Chinese Herb. Med.* (2018) doi:10.1016/j.chmed.2018.08.003.

20. Sharma, H. & Garg, M. A review of traditional use, phytoconstituents and biological activities of Himalayan yew, Taxus wallichiana. *J. Integr. Med.* **13**, 80–90 (2015).

21. Sharma, N. *et al.* Thalictrum foliolosum: A lesser unexplored medicinal herb from the Himalayan region as a source of valuable benzyl isoquinoline alkaloids. *J. Ethnopharmacol.* **255**, 112736 (2020).

22. & Wolfman, L. S. B. A. 済無No Title No Title. *J. Chem. Inf. Model.* **53**, 1689–1699 (2013).

23. Chi, S. *et al.* Genus Tinospora: Ethnopharmacology, Phytochemistry, and Pharmacology. *Evidence-based Complement. Altern. Med.* **2016**, (2016).

24. Peng, W. *et al.* Toona sinensis: a comprehensive review on its traditional usages, phytochemisty, pharmacology and toxicology. *Rev. Bras. Farmacogn.* (2018) doi:10.1016/j.bjp.2018.07.009.

25. Congjin, C., Yue, L., Dankui, L., Zhangfa, T. & Guoen, Y. Chemical composition of Toona sinensis essential oil and DPPH scavenging activity of different fractions in ethanol extract of Toona sinensis from China. **17**, (2013).

26. Ahmed, S., Liu, H., Ahmad, A. & Akram, W. Characterization of Anti-bacterial Compounds from the Seed Coat of Chinese Windmill Palm Tree (Trachycarpus fortunei). **8**, 1–11 (2017).

27. Ohiri, R. C. GC/MS analysisof Tremella fuciformis. **89**, (2017).

28. Ouyang, X. K., Jin, M. C. & He, C. H. Simultaneous determination of four sesquiterpene alkaloids in Tripterygium wilfordii Hook. F. extracts by high-performance liquid chromatography. *Phytochem.*

Anal. **18**, 320–325 (2007).

29. Chen, Y., Yang, G. Z., Zhao, S. & Li, Y. C. Diterpenoids from tripterygium wilfordii Hook. f. *Linchan Huaxue Yu Gongye/Chemistry Ind. For. Prod.* **25**, 35–38 (2005).

30. Gao, C. *et al.* Chemical constituents from the roots of Tripterygium wilfordii and their cytotoxic activity. *J. Asian Nat. Prod. Res.* **19**, 725–731 (2017).

31. Faridah DN, Andarwulan N, Purnomo EP, Saifatah L, K. S. Ournal of. *Asian J. Chem.* **26**, 70–73 (2014).

32. Lv, H. *et al.* The genus Tripterygium: A phytochemistry and pharmacological review. *Fitoterapia* **137**, (2019).

33. Xu, R., Fidler, J. M. & Musser, J. H. Bioactive compounds from Tripterygium wilfordii. *Stud. Nat. Prod. Chem.* **32**, 773–801 (2005).

34. Butnariu, M. & Bostan, C. Antimicrobial and anti-inflammatory activities of the volatile oil compounds from tropaeolum majus l. (nasturtium). *African J. Biotechnol.* **10**, 5900–5909 (2011).

35. Rebiai, A. PCBS J ournal. (2014) doi:10.13140/2.1.5049.9208.

36. Garzón, G. A., Manns, D. C., Riedl, K., Schwartz, S. J. & Padilla-Zakour, O. Identification of phenolic compounds in petals of nasturtium flowers (Tropaeolum majus) by high-performance liquid chromatography coupled to mass spectrometry and determination of oxygen radical absorbance capacity (ORAC). *J. Agric. Food Chem.* **63**, 1803–1811 (2015).

37. Bazylko, A. *et al.* Comparison of antioxidant, anti-inflammatory, antimicrobial activity and chemical composition of aqueous and hydroethanolic extracts of the herb of Tropaeolum majus L. *Ind. Crops Prod.* **50**, 88–94 (2013).

38. Korinek, M. *et al.* Anti-allergic hydroxy fatty acids from Typhonium blumei explored through ChemGPS-NP. *Front. Pharmacol.* **8**, (2017).

39. Zhang, Q., Zhao, J. J., Xu, J., Feng, F. & Qu, W. Medicinal uses, phytochemistry and pharmacology of the genus Uncaria. *J. Ethnopharmacol.* **173**, 48–80 (2015).

40. Wei, X. *et al.* Indole Alkaloids Inhibiting Neural Stem Cell from Uncaria rhynchophylla. *Nat. Products Bioprospect.* (2017) doi:10.1007/s13659-017-0141-y.

41. Zhang, J. *et al.* Chemical and biological comparison of different sections of Uncaria rhynchophylla (Gou-Teng). (2017) doi:10.1177/1469066717694044.

42. Constituents, C. Uncaria rhynchophylla. (1992).

43. Xian, Y. *et al.* Bioassay-Guided Isolation of Neuroprotective Compounds from Uncaria rhynchophylla against Beta-Amyloid-Induced Neurotoxicity. **2012**, (2012).

44. Wang, Y. *et al.* Phytochemical constituents from Uncaria rhynchophylla in human carboxylesterase 2 inhibition: Kinetics and interaction mechanism merged with docking simulations. *Phytomedicine* (2018) doi:10.1016/j.phymed.2018.10.006.

45. Ibrahim, M. *et al.* Investigations of phytochemical constituents and their pharmacological properties isolated from the genus urtica: Critical review and analysis. *Crit. Rev. Eukaryot. Gene Expr.* **28**, 25–66 (2018).

46. Kregiel, D., Pawlikowska, E. & Antolak, H. Urtica spp.: Ordinary plants with extraordinary properties. *Molecules* **23**, 1–21 (2018).

TABLE S3 The binding energy of the all compounds generated from AutoDock Vina.

Ligand	Binding Affinity	rmsd/ub	rmsd/lb
6lu7_EM_72_uff_E=70.72	-5.8	0	0
6lu7_EM_126_uff_E=57.33	-5.1	0	0
6lu7_EM_127_uff_E=89.11	-5.8	0	0
6lu7_EM_135_uff_E=62.95	-5.8	0	0
6lu7_EM_240_uff_E=52.88	-5	0	0
6lu7_EM_243_uff_E=59.30	-5.9	0	0
6lu7_EM_247_uff_E=88.22	-3.8	0	0
6lu7_EM_289_uff_E=55.42	-5.2	0	0
6lu7_EM_305_uff_E=103.14	-3.3	0	0
6lu7_EM_332_uff_E=99.78	-5.3	0	0
6lu7_EM_338_uff_E=73.50	-6.1	0	0
6lu7_EM_370_uff_E=77.82	-5.7	0	0
6lu7_EM_379_uff_E=30.18	-4.9	0	0
6lu7_EM_454_uff_E=27.41	-4.2	0	0
6lu7_EM_595_uff_E=124.04	-4.9	0	0
6lu7_EM_750_uff_E=27.51	-3.8	0	0
6lu7_EM_785_uff_E=52.91	-4.7	0	0
6lu7_EM_932_uff_E=195.80	-6.9	0	0
6lu7_EM_936_uff_E=74.86	-5.2	0	0
6lu7_EM_957_uff_E=39.46	-4.3	0	0
6lu7_EM_985_uff_E=57.46	-5.1	0	0
6lu7_EM_995_uff_E=161.17	-6.1	0	0
6lu7_EM_1001_uff_E=91.78	-5.2	0	0
6lu7_EM_1017_uff_E=151.39	-6	0	0
6lu7_EM_1110_uff_E=26.16	-4.7	0	0
6lu7_EM_1174_uff_E=44.82	-5.2	0	0
6lu7_EM_1183_uff_E=79.78	-5.1	0	0
6lu7_EM_2214_uff_E=155.34	-5.4	0	0
6lu7_EM_2346_uff_E=79.77	-4.7	0	0
6lu7_EM_2353_uff_E=579.86	-5.4	0	0
6lu7_EM_2518_uff_E=98.61	-6	0	0
6lu7_EM_2950_uff_E=185.78	-7.1	0	0
6lu7_EM_2969_uff_E=38.20	-5	0	0
6lu7_EM_3026_uff_E=212.61	-5.4	0	0
6lu7_EM_3220_uff_E=196.51	-7.3	0	0
6lu7_EM_3314_uff_E=169.59	-5.3	0	0
6lu7_EM_3469_uff_E=78.01	-6	0	0
6lu7_EM_3893_uff_E=43.33	-5.2	0	0
6lu7_EM_4133_uff_E=86.76	-5.4	0	0
6lu7_EM_4735_uff_E=245.94	-6.5	0	0
6lu7_EM_5417_uff_E=594.17	-6.6	0	0
6lu7_EM_5574_uff_E=318.49	-5.1	0	0
6lu7_EM_5634_uff_E=57.21	-5.2	0	0
6lu7_EM_5770_uff_E=859.40	-6.4	0	0
6lu7_EM_5793_uff_E=184.96	-4.6	0	0
6lu7_EM_5950_uff_E=33.09	-3.9	0	0
6lu7_EM_5951_uff_E=74.76	-4.1	0	0
6lu7_EM_5960_uff_E=39.14	-4.5	0	0
6lu7_EM_5962_uff_E=63.08	-4.6	0	0
6lu7_EM_5988_uff_E=487.38	-5.3	0	0
6lu7_EM_5997_uff_E=549.32	-7.4	0	0
6lu7_EM_6029_uff_E=306.70	-6.2	0	0
6lu7_EM_6036_uff_E=190.36	-5.3	0	0
6lu7_EM_6043_uff_E=312.52	-5.4	0	0
6lu7_EM_6057_uff_E=109.06	-5.8	0	0
6lu7_EM_6106_uff_E=72.66	-4.7	0	0
6lu7_EM_6137_uff_E=94.18	-4	0	0
6lu7_EM_6140_uff_E=104.20	-5.6	0	0
6lu7_EM_6251_uff_E=158.46	-4.8	0	0
6lu7_EM_6255_uff_E=385.08	-6	0	0
6lu7_EM_6274_uff_E=256.87	-5	0	0
6lu7_EM_6287_uff_E=55.97	-4.2	0	0
6lu7_EM_6288_uff_E=64.57	-4.7	0	0
6lu7_EM_6293_uff_E=182.03	-7.1	0	0

6lu7_EM_6305_uff_E=333.02	-6	0	0
6lu7_EM_6306_uff_E=69.94	-4.3	0	0
6lu7_EM_6322_uff_E=70.50	-4.8	0	0
6lu7_EM_6508_uff_E=156.41	-5.7	0	0
6lu7_EM_6654_uff_E=633.64	-5.1	0	0
6lu7_EM_6683_uff_E=197.40	-7.2	0	0
6lu7_EM_6780_uff_E=133.80	-6.9	0	0
6lu7_EM_6782_uff_E=255.99	-5.7	0	0
6lu7_EM_6919_uff_E=208.82	-4.9	0	0
6lu7_EM_7057_uff_E=95.90	-6.5	0	0
6lu7_EM_7150_uff_E=68.70	-5.1	0	0
6lu7_EM_7302_uff_E=151.57	-3.8	0	0
6lu7_EM_7427_uff_E=388.09	-5.9	0	0
6lu7_EM_7428_uff_E=94.73	-5.7	0	0
6lu7_EM_7439_uff_E=108.57	-5	0	0
6lu7_EM_7460_uff_E=154.38	-5.1	0	0
6lu7_EM_7461_uff_E=63.24	-5.3	0	0
6lu7_EM_7462_uff_E=122.04	-4.9	0	0
6lu7_EM_7463_uff_E=91.91	-5.6	0	0
6lu7_EM_7501_uff_E=71.29	-4.9	0	0
6lu7_EM_7695_uff_E=106.64	-5.1	0	0
6lu7_EM_7800_uff_E=53.04	-4.3	0	0
6lu7_EM_7824_uff_E=33.29	-3.7	0	0
6lu7_EM_7972_uff_E=103.11	-3.2	0	0
6lu7_EM_8029_uff_E=199.87	-3.3	0	0
6lu7_EM_8042_uff_E=83.23	-5	0	0
6lu7_EM_8049_uff_E=65.24	-4.1	0	0
6lu7_EM_8078_uff_E=30.11	-3.6	0	0
6lu7_EM_8129_uff_E=36.31	-4.3	0	0
6lu7_EM_8130_uff_E=24.07	-3.8	0	0
6lu7_EM_8138_uff_E=49.33	-4.2	0	0
6lu7_EM_8141_uff_E=23.72	-3.6	0	0
6lu7_EM_8143_uff_E=37.18	-4	0	0
6lu7_EM_8158_uff_E=33.81	-4.6	0	0
6lu7_EM_8164_uff_E=33.74	-4.1	0	0
6lu7_EM_8176_uff_E=33.04	-4	0	0
6lu7_EM_8180_uff_E=40.64	-4.7	0	0
6lu7_EM_8181_uff_E=66.14	-4.2	0	0
6lu7_EM_8194_uff_E=41.12	-4.2	0	0
6lu7_EM_8369_uff_E=63.99	-4.4	0	0
6lu7_EM_8468_uff_E=150.75	-5.5	0	0
6lu7_EM_8575_uff_E=210.98	-5.9	0	0
6lu7_EM_8655_uff_E=168.50	-4.8	0	0
6lu7_EM_8785_uff_E=85.97	-5.6	0	0
6lu7_EM_8794_uff_E=234.72	-4.5	0	0
6lu7_EM_8830_uff_E=60.42	-4.2	0	0
6lu7_EM_8892_uff_E=24.06	-4.7	0	0
6lu7_EM_8969_uff_E=497.11	-7.3	0	0
6lu7_EM_9064_uff_E=204.84	-7.4	0	0
6lu7_EM_9121_uff_E=435.87	-7.2	0	0
6lu7_EM_9210_uff_E=86.84	-5	0	0
6lu7_EM_9294_uff_E=150.09	-5	0	0
6lu7_EM_9415_uff_E=463.48	-6.6	0	0
6lu7_EM_9895_uff_E=172.51	-5.4	0	0
6lu7_EM_10143_uff_E=597.01	-7.2	0	0
6lu7_EM_10151_uff_E=237.56	-7.1	0	0
6lu7_EM_10168_uff_E=201.65	-7.3	0	0
6lu7_EM_10177_uff_E=620.08	-7.4	0	0
6lu7_EM_10206_uff_E=1064.03	-7.2	0	0
6lu7_EM_10207_uff_E=212.95	-7.4	0	0
6lu7_EM_10208_uff_E=215.65	-7.4	0	0
6lu7_EM_10215_uff_E=571.00	-6.9	0	0
6lu7_EM_10231_uff_E=304.43	-5.1	0	0
6lu7_EM_10235_uff_E=197.51	-5.7	0	0
6lu7_EM_10349_uff_E=28.89	-4.7	0	0
6lu7_EM_10393_uff_E=97.81	-5.6	0	0

6lu7_EM_10408_uff_E=120.66	-4.6	0	0
6lu7_EM_10416_uff_E=2180.77	-4.8	0	0
6lu7_EM_10465_uff_E=60.66	-4.8	0	0
6lu7_EM_10494_uff_E=689.79	-6.9	0	0
6lu7_EM_10582_uff_E=649.92	-5.3	0	0
6lu7_EM_10639_uff_E=212.65	-7.3	0	0
6lu7_EM_10703_uff_E=80.24	-5.2	0	0
6lu7_EM_10742_uff_E=110.08	-5.7	0	0
6lu7_EM_10976_uff_E=41.95	-4.4	0	0
6lu7_EM_11005_uff_E=51.08	-4.6	0	0
6lu7_EM_11006_uff_E=47.83	-3.8	0	0
6lu7_EM_11066_uff_E=599.93	-7.2	0	0
6lu7_EM_11142_uff_E=124.80	-5.4	0	0
6lu7_EM_11230_uff_E=155.64	-4.8	0	0
6lu7_EM_11468_uff_E=134.28	-5.7	0	0
6lu7_EM_11503_uff_E=100.47	-4.8	0	0
6lu7_EM_11605_uff_E=117.50	-3.9	0	0
6lu7_EM_11915_uff_E=102.80	-6.4	0	0
6lu7_EM_12398_uff_E=50.10	-3.7	0	0
6lu7_EM_12523_uff_E=140.35	-4.3	0	0
6lu7_EM_12530_uff_E=46.39	-4.4	0	0
6lu7_EM_12921_uff_E=2186.45	-4.4	0	0
6lu7_EM_13229_uff_E=89.44	-5.5	0	0
6lu7_EM_13250_uff_E=88.97	-5.7	0	0
6lu7_EM_13849_uff_E=53.38	-4.2	0	0
6lu7_EM_14257_uff_E=30.71	-4	0	0
6lu7_EM_14896_uff_E=713.31	-5.1	0	0
6lu7_EM_15611_uff_E=58.50	-4.7	0	0
6lu7_EM_15612_uff_E=60.32	-4.1	0	0
6lu7_EM_15979_uff_E=127.95	-4.4	0	0
6lu7_EM_16217_uff_E=55.80	-4.3	0	0
6lu7_EM_16331_uff_E=42.40	-4.2	0	0
6lu7_EM_16913_uff_E=506.65	-5.3	0	0
6lu7_EM_17868_uff_E=1384.84	-4.8	0	0
6lu7_EM_18721_uff_E=459.52	-7	0	0
6lu7_EM_18950_uff_E=195.52	-5	0	0
6lu7_EM_19009_uff_E=523.49	-6.7	0	0
6lu7_EM_19212_uff_E=710.49	-7.2	0	0
6lu7_EM_19725_uff_E=770.21	-5.9	0	0
6lu7_EM_21057_uff_E=42.23	-3.9	0	0
6lu7_EM_21205_uff_E=77.38	-4.4	0	0
6lu7_EM_22311_uff_E=102.50	-5.2	0	0
6lu7_EM_22955_uff_E=436.93	-7.3	0	0
6lu7_EM_23518_uff_E=62.94	-4.7	0	0
6lu7_EM_23741_uff_E=1468.22	-3.9	0	0
6lu7_EM_23915_uff_E=955.76	-7	0	0
6lu7_EM_24197_uff_E=70.53	-4.5	0	0
6lu7_EM_25310_uff_E=179.95	-5.2	0	0
6lu7_EM_28454_uff_E=85.05	-4	0	0
6lu7_EM_28469_uff_E=168.40	-5.5	0	0
6lu7_EM_28565_uff_E=522.34	-6.7	0	0
6lu7_EM_29566_uff_E=1102.39	-6.1	0	0
6lu7_EM_31209_uff_E=261.93	-5.1	0	0
6lu7_EM_31244_uff_E=74.15	-4.7	0	0
6lu7_EM_31253_uff_E=90.99	-4.2	0	0
6lu7_EM_31268_uff_E=212.35	-3.1	0	0
6lu7_EM_31283_uff_E=83.49	-3.8	0	0
6lu7_EM_31285_uff_E=24.64	-3.9	0	0
6lu7_EM_31703_uff_E=616.59	-7	0	0
6lu7_EM_33032_uff_E=55.55	-4.8	0	0
6lu7_EM_33624_uff_E=517.75	-5.3	0	0
6lu7_EM_38762_uff_E=63.64	-4	0	0
6lu7_EM_43595_uff_E=911.77	-6.1	0	0
6lu7_EM_60961_uff_E=543.04	-6.5	0	0
6lu7_EM_60985_uff_E=117.40	-4.6	0	0
6lu7_EM_61030_uff_E=46.56	-4.4	0	0

6lu7_EM_61126_uff_E=654.84	-5.2	0	0
6lu7_EM_61303_uff_E=48.96	-4.1	0	0
6lu7_EM_62321_uff_E=132.77	-4.8	0	0
6lu7_EM_62367_uff_E=1462.27	-5	0	0
6lu7_EM_62566_uff_E=899.87	-5.7	0	0
6lu7_EM_62752_uff_E=193.41	-4.5	0	0
6lu7_EM_64945_uff_E=805.65	-7.2	0	0
6lu7_EM_64971_uff_E=793.31	-7.4	0	0
6lu7_EM_65064_uff_E=325.39	-7.3	0	0
6lu7_EM_65366_uff_E=1548.87	-6.8	0	0
6lu7_EM_65373_uff_E=444.03	-6.2	0	0
6lu7_EM_65411_uff_E=4795.40	-7.4	0	0
6lu7_EM_65575_uff_E=580.54	-6	0	0
6lu7_EM_67328_uff_E=33.86	-3.9	0	0
6lu7_EM_68071_uff_E=214.20	-7	0	0
6lu7_EM_68167_uff_E=87.11	-4.4	0	0
6lu7_EM_69867_uff_E=287.71	-6.2	0	0
6lu7_EM_70627_uff_E=83.63	-5	0	0
6lu7_EM_72276_uff_E=230.94	-7.5	0	0
6lu7_EM_72277_uff_E=237.97	-7.3	0	0
6lu7_EM_72281_uff_E=283.48	-7.4	0	0
6lu7_EM_72310_uff_E=445.58	-7.3	0	0
6lu7_EM_72323_uff_E=720.42	-7.1	0	0
6lu7_EM_72326_uff_E=948.71	-7.2	0	0
6lu7_EM_72369_uff_E=673.25	-7.3	0	0
6lu7_EM_72537_uff_E=237.73	-7.1	0	0
6lu7_EM_72610_uff_E=267.25	-5.9	0	0
6lu7_EM_73145_uff_E=694.14	-7.1	0	0
6lu7_EM_73170_uff_E=3374.53	-6.8	0	0
6lu7_EM_73193_uff_E=781.69	-6.8	0	0
6lu7_EM_73253_uff_E=479.66	-7.1	0	0
6lu7_EM_73307_uff_E=87.58	-4.8	0	0
6lu7_EM_73330_uff_E=771.59	-6.8	0	0
6lu7_EM_73337_uff_E=577.41	-6.6	0	0
6lu7_EM_73399_uff_E=626.81	-7.4	0	0
6lu7_EM_73481_uff_E=958.07	-6.7	0	0
6lu7_EM_73568_uff_E=749.04	-6.5	0	0
6lu7_EM_73571_uff_E=212.55	-7.2	0	0
6lu7_EM_73659_uff_E=837.07	-7.2	0	0
6lu7_EM_74416_uff_E=54.58	-3.9	0	0
6lu7_EM_75704_uff_E=40.47	-4.5	0	0
6lu7_EM_77466_uff_E=115.76	-4.1	0	0
6lu7_EM_77547_uff_E=94.40	-5.8	0	0
6lu7_EM_78435_uff_E=265.89	-5.4	0	0
6lu7_EM_79043_uff_E=1556.99	-4.7	0	0
6lu7_EM_81101_uff_E=64.17	-4.3	0	0
6lu7_EM_81747_uff_E=93.93	-4.9	0	0
6lu7_EM_82143_uff_E=474.62	-7.1	0	0
6lu7_EM_82755_uff_E=106.56	-5.7	0	0
6lu7_EM_86609_uff_E=1517.00	-6.4	0	0
6lu7_EM_86770_uff_E=57.18	-4.2	0	0
6lu7_EM_89047_uff_E=304.37	-5.9	0	0
6lu7_EM_91354_uff_E=1698.37	-6.3	0	0
6lu7_EM_91439_uff_E=860.89	-6.2	0	0
6lu7_EM_91457_uff_E=194.77	-5.9	0	0
6lu7_EM_91466_uff_E=243.56	-6.6	0	0
6lu7_EM_91472_uff_E=973.63	-6.8	0	0
6lu7_EM_91510_uff_E=496.67	-6.7	0	0
6lu7_EM_92123_uff_E=298.25	-6.6	0	0
6lu7_EM_92139_uff_E=113.33	-5.5	0	0
6lu7_EM_92158_uff_E=853.30	-6.6	0	0
6lu7_EM_92231_uff_E=1727.92	-6.3	0	0
6lu7_EM_92780_uff_E=122.60	-4.6	0	0
6lu7_EM_92987_uff_E=68.07	-4.4	0	0
6lu7_EM_93017_uff_E=281.53	-6.8	0	0
6lu7_EM_93081_uff_E=1562.49	-6.1	0	0

6lu7_EM_94149_uff_E=617.77	-6.9	0	0
6lu7_EM_94196_uff_E=314.16	-6.2	0	0
6lu7_EM_94221_uff_E=122.65	-5.2	0	0
6lu7_EM_94266_uff_E=1497.23	-5.2	0	0
6lu7_EM_96191_uff_E=252.44	-6.9	0	0
6lu7_EM_97560_uff_E=229.53	-7	0	0
6lu7_EM_98912_uff_E=394.93	-6.8	0	0
6lu7_EM_99300_uff_E=197.16	-7.2	0	0
6lu7_EM_99474_uff_E=741.60	-7	0	0
6lu7_EM_99516_uff_E=880.45	-6.9	0	0
6lu7_EM_99693_uff_E=326.11	-7.3	0	0
6lu7_EM_99938_uff_E=469.76	-7.5	0	0
6lu7_EM_100067_uff_E=737.59	-7.2	0	0
6lu7_EM_100231_uff_E=1073.13	-7.3	0	0
6lu7_EM_100332_uff_E=283.69	-6.1	0	0
6lu7_EM_100781_uff_E=713.26	-7.5	0	0
6lu7_EM_101300_uff_E=623.26	-6.8	0	0
6lu7_EM_101731_uff_E=505.04	-5.8	0	0
6lu7_EM_102667_uff_E=738.29	-5.3	0	0
6lu7_EM_107905_uff_E=319.94	-6.9	0	0
6lu7_EM_107971_uff_E=542.73	-6.8	0	0
6lu7_EM_107985_uff_E=4889.50	-7.2	0	0
6lu7_EM_114829_uff_E=185.86	-7.5	0	0
6lu7_EM_115012_uff_E=928.34	-7	0	0
6lu7_EM_115127_uff_E=732.66	-6.9	0	0
6lu7_EM_115250_uff_E=783.38	-7.2	0	0
6lu7_EM_115269_uff_E=289.50	-6.4	0	0
6lu7_EM_117440_uff_E=372.64	-6.7	0	0
6lu7_EM_117900_uff_E=505.47	-7.1	0	0
6lu7_EM_119034_uff_E=876.98	-6.7	0	0
6lu7_EM_119204_uff_E=448.72	-6.8	0	0
6lu7_EM_119205_uff_E=472.04	-6.3	0	0
6lu7_EM_119258_uff_E=429.33	-6	0	0
6lu7_EM_121313_uff_E=1058.49	-7.4	0	0
6lu7_EM_122724_uff_E=726.35	-7.1	0	0
6lu7_EM_122844_uff_E=855.65	-7.3	0	0
6lu7_EM_122850_uff_E=233.92	-7.2	0	0
6lu7_EM_124062_uff_E=170.95	-7.2	0	0
6lu7_EM_124214_uff_E=953.23	-7.4	0	0
6lu7_EM_124219_uff_E=353.67	-7.5	0	0
6lu7_EM_128108_uff_E=1024.40	-7.3	0	0
6lu7_EM_129520_uff_E=810.45	-6.1	0	0
6lu7_EM_129521_uff_E=984.56	-6.9	0	0
6lu7_EM_132415_uff_E=347.31	-7.3	0	0
6lu7_EM_133323_uff_E=492.05	-6.6	0	0
6lu7_EM_133766_uff_E=535.42	-6.8	0	0
6lu7_EM_134697_uff_E=745.24	-6	0	0
6lu7_EM_136419_uff_E=353.87	-6.4	0	0
6lu7_EM_145742_uff_E=182.22	-4.4	0	0
6lu7_EM_146798_uff_E=440.35	-6.6	0	0
6lu7_EM_147299_uff_E=483.46	-7	0	0
6lu7_EM_149600_uff_E=550.32	-7.4	0	0
6lu7_EM_151529_uff_E=931.35	-6.7	0	0
6lu7_EM_154272_uff_E=1545.99	-7.1	0	0
6lu7_EM_155011_uff_E=382.26	-7.2	0	0
6lu7_EM_155380_uff_E=462.41	-6.9	0	0
6lu7_EM_155381_uff_E=515.34	-6.7	0	0
6lu7_EM_156286_uff_E=509.39	-7.2	0	0
6lu7_EM_156875_uff_E=413.76	-7.5	0	0
6lu7_EM_157561_uff_E=2088.64	-6.4	0	0
6lu7_EM_158477_uff_E=958.72	-6.6	0	0
6lu7_EM_159795_uff_E=1059.74	-7.3	0	0
6lu7_EM_160180_uff_E=578.99	-6.2	0	0
6lu7_EM_160476_uff_E=331.53	-7.2	0	0
6lu7_EM_160481_uff_E=211.16	-7.2	0	0
6lu7_EM_160483_uff_E=376.76	-7.4	0	0

6lu7_EM_160487_uff_E=329.16	-7.4	0	0
6lu7_EM_160490_uff_E=259.28	-7.2	0	0
6lu7_EM_160497_uff_E=2091.17	-6.6	0	0
6lu7_EM_160500_uff_E=524.91	-7.3	0	0
6lu7_EM_160712_uff_E=202.48	-7.1	0	0
6lu7_EM_160817_uff_E=182.64	-7.5	0	0
6lu7_EM_161336_uff_E=739.52	-6.7	0	0
6lu7_EM_161379_uff_E=420.26	-7.1	0	0
6lu7_EM_161487_uff_E=647.75	-6.9	0	0
6lu7_EM_161665_uff_E=511.20	-6.9	0	0
6lu7_EM_162138_uff_E=872.33	-6.7	0	0
6lu7_EM_163067_uff_E=949.38	-7	0	0
6lu7_EM_164893_uff_E=258.39	-6.9	0	0
6lu7_EM_165839_uff_E=616.53	-6.7	0	0
6lu7_EM_167718_uff_E=503.12	-6.7	0	0
6lu7_EM_167825_uff_E=729.84	-6.3	0	0
6lu7_EM_168985_uff_E=608.48	-6.9	0	0
6lu7_EM_169727_uff_E=535.79	-7	0	0
6lu7_EM_173183_uff_E=573.30	-6.9	0	0
6lu7_EM_173273_uff_E=449.08	-7	0	0
6lu7_EM_173713_uff_E=479.14	-6.9	0	0
6lu7_EM_174362_uff_E=837.92	-7.1	0	0
6lu7_EM_177562_uff_E=566.07	-7.4	0	0
6lu7_EM_178034_uff_E=498.56	-7.3	0	0
6lu7_EM_178222_uff_E=732.35	-6.8	0	0
6lu7_EM_179390_uff_E=1116.22	-7.1	0	0
6lu7_EM_181384_uff_E=1041.29	-7.3	0	0
6lu7_EM_182449_uff_E=220.58	-7.5	0	0
6lu7_EM_182497_uff_E=943.83	-7.5	0	0
6lu7_EM_185605_uff_E=513.90	-7.2	0	0
6lu7_EM_185740_uff_E=223.68	-5.9	0	0
6lu7_EM_187808_uff_E=646.30	-6.9	0	0
6lu7_EM_188289_uff_E=843.41	-7.4	0	0
6lu7_EM_188316_uff_E=397.63	-7.1	0	0
6lu7_EM_188442_uff_E=438.43	-7.3	0	0
6lu7_EM_188999_uff_E=599.86	-7.3	0	0
6lu7_EM_194654_uff_E=432.12	-6.1	0	0
6lu7_EM_196978_uff_E=150.91	-7.2	0	0
6lu7_EM_197001_uff_E=638.74	-7.1	0	0
6lu7_EM_197582_uff_E=1944.57	-7.2	0	0
6lu7_EM_197857_uff_E=574.93	-7.1	0	0
6lu7_EM_222284_uff_E=590.88	-7.5	0	0
6lu7_EM_227613_uff_E=755.45	-6.7	0	0
6lu7_EM_232703_uff_E=3020.63	-4.7	0	0
6lu7_EM_234823_uff_E=545.69	-6.7	0	0
6lu7_EM_237332_uff_E=252.62	-4.6	0	0
6lu7_EM_243793_uff_E=110.97	-4.9	0	0
6lu7_EM_246983_uff_E=914.38	-7.4	0	0
6lu7_EM_251566_uff_E=805.76	-6.7	0	0
6lu7_EM_253793_uff_E=890.29	-6.8	0	0
6lu7_EM_258412_uff_E=773.28	-7.1	0	0
6lu7_EM_259846_uff_E=928.19	-6.6	0	0
6lu7_EM_276591_uff_E=430.82	-6.7	0	0
6lu7_EM_294491_uff_E=4877.37	-7.3	0	0
6lu7_EM_294711_uff_E=491.05	-4.9	0	0
6lu7_EM_307918_uff_E=304.34	-6.8	0	0
6lu7_EM_322636_uff_E=107.16	-5.7	0	0
6lu7_EM_332426_uff_E=800.44	-7	0	0
6lu7_EM_333544_uff_E=626.23	-7.1	0	0
6lu7_EM_336327_uff_E=346.46	-7.1	0	0
6lu7_EM_363209_uff_E=428.28	-7.3	0	0
6lu7_EM_414564_uff_E=237.63	-4.2	0	0
6lu7_EM_439336_uff_E=590.39	-7.4	0	0
6lu7_EM_439503_uff_E=324.93	-6.4	0	0
6lu7_EM_439533_uff_E=241.36	-7.2	0	0
6lu7_EM_439653_uff_E=417.03	-6.8	0	0

6lu7_EM_439655_uff_E=76.10	-4.6	0	0
6lu7_EM_440595_uff_E=342.56	-6.6	0	0
6lu7_EM_440967_uff_E=713.31	-5.1	0	0
6lu7_EM_440968_uff_E=633.64	-5.1	0	0
6lu7_EM_441005_uff_E=241.83	-5.9	0	0
6lu7_EM_441300_uff_E=1759.65	-6.8	0	0
6lu7_EM_441564_uff_E=475.10	-7.1	0	0
6lu7_EM_441678_uff_E=776.43	-7.3	0	0
6lu7_EM_441975_uff_E=545.68	-7.1	0	0
6lu7_EM_442001_uff_E=515.21	-7.1	0	0
6lu7_EM_442194_uff_E=543.98	-7	0	0
6lu7_EM_442350_uff_E=689.95	-7	0	0
6lu7_EM_442393_uff_E=144.71	-6.2	0	0
6lu7_EM_442437_uff_E=418.68	-7.3	0	0
6lu7_EM_442501_uff_E=147.23	-5.1	0	0
6lu7_EM_442544_uff_E=628.51	-7.2	0	0
6lu7_EM_442675_uff_E=374.79	-7.1	0	0
6lu7_EM_442695_uff_E=773.81	-5.7	0	0
6lu7_EM_442702_uff_E=236.82	-7	0	0
6lu7_EM_442741_uff_E=1904.06	-6.6	0	0
6lu7_EM_442765_uff_E=378.47	-7.4	0	0
6lu7_EM_442813_uff_E=528.17	-5.6	0	0
6lu7_EM_442827_uff_E=692.08	-6.9	0	0
6lu7_EM_442896_uff_E=738.69	-6	0	0
6lu7_EM_442985_uff_E=717.71	-6	0	0
6lu7_EM_443024_uff_E=850.21	-7.2	0	0
6lu7_EM_443027_uff_E=870.68	-6.8	0	0
6lu7_EM_443158_uff_E=118.52	-4.5	0	0
6lu7_EM_443639_uff_E=225.40	-7.4	0	0
6lu7_EM_443648_uff_E=558.56	-7.3	0	0
6lu7_EM_444539_uff_E=86.61	-5.8	0	0
6lu7_EM_445070_uff_E=148.85	-5.5	0	0
6lu7_EM_445154_uff_E=172.22	-6.9	0	0
6lu7_EM_445638_uff_E=85.31	-4.4	0	0
6lu7_EM_445639_uff_E=80.35	-5.2	0	0
6lu7_EM_445641_uff_E=103.56	-4.6	0	0
6lu7_EM_445858_uff_E=177.42	-6.1	0	0
6lu7_EM_446284_uff_E=172.66	-5.7	0	0
6lu7_EM_448438_uff_E=3509.77	-6.8	0	0
6lu7_EM_457194_uff_E=530.67	-7.2	0	0
6lu7_EM_457825_uff_E=1030.07	-6.9	0	0
6lu7_EM_466268_uff_E=207.82	-6.8	0	0
6lu7_EM_470665_uff_E=756.48	-6.8	0	0
6lu7_EM_471426_uff_E=896.18	-7.3	0	0
6lu7_EM_485186_uff_E=638.68	-7.3	0	0
6lu7_EM_503732_uff_E=730.31	-6	0	0
6lu7_EM_503734_uff_E=592.03	-5.9	0	0
6lu7_EM_517973_uff_E=80.62	-4.8	0	0
6lu7_EM_519186_uff_E=65.09	-4	0	0
6lu7_EM_519330_uff_E=125.36	-6.1	0	0
6lu7_EM_519545_uff_E=534.83	-5.9	0	0
6lu7_EM_519743_uff_E=562.91	-6.2	0	0
6lu7_EM_519857_uff_E=221.98	-6	0	0
6lu7_EM_519872_uff_E=70.85	-4.4	0	0
6lu7_EM_520710_uff_E=59.55	-4.7	0	0
6lu7_EM_527418_uff_E=740.60	-5.8	0	0
6lu7_EM_528708_uff_E=188.62	-6.3	0	0
6lu7_EM_530421_uff_E=1568.36	-5.6	0	0
6lu7_EM_530426_uff_E=1658.89	-5.9	0	0
6lu7_EM_534446_uff_E=99.80	-4	0	0
6lu7_EM_535346_uff_E=654.25	-5.9	0	0
6lu7_EM_536442_uff_E=185.24	-5.9	0	0
6lu7_EM_536595_uff_E=212.88	-6	0	0
6lu7_EM_536727_uff_E=208.75	-5.7	0	0
6lu7_EM_540645_uff_E=213.98	-6.2	0	0
6lu7_EM_541526_uff_E=190.55	-6.9	0	0

6lu7_EM_543312_uff_E=184.58	-4.3	0	0
6lu7_EM_545799_uff_E=126.49	-4.5	0	0
6lu7_EM_545889_uff_E=91.62	-4.3	0	0
6lu7_EM_545955_uff_E=88.84	-4.4	0	0
6lu7_EM_548865_uff_E=59.07	-5	0	0
6lu7_EM_550058_uff_E=478.86	-6.7	0	0
6lu7_EM_561668_uff_E=105.71	-4.8	0	0
6lu7_EM_570529_uff_E=582.59	-6.2	0	0
6lu7_EM_573721_uff_E=53.33	-4.3	0	0
6lu7_EM_578229_uff_E=173.01	-5.2	0	0
6lu7_EM_583791_uff_E=275.98	-6.2	0	0
6lu7_EM_584545_uff_E=142.22	-6.4	0	0
6lu7_EM_585744_uff_E=1354.31	-6	0	0
6lu7_EM_593889_uff_E=252.74	-6.1	0	0
6lu7_EM_594593_uff_E=1675.88	-6.2	0	0
6lu7_EM_612605_uff_E=208.84	-5.9	0	0
6lu7_EM_619267_uff_E=220.05	-6.2	0	0
6lu7_EM_630859_uff_E=623.26	-7.2	0	0
6lu7_EM_637213_uff_E=449.70	-7.4	0	0
6lu7_EM_637541_uff_E=90.97	-5.9	0	0
6lu7_EM_637542_uff_E=218.20	-5.6	0	0
6lu7_EM_637566_uff_E=110.10	-4.9	0	0
6lu7_EM_637584_uff_E=545.43	-6.8	0	0
6lu7_EM_637775_uff_E=206.17	-5.9	0	0
6lu7_EM_638072_uff_E=248.91	-6.1	0	0
6lu7_EM_643684_uff_E=131.80	-4.4	0	0
6lu7_EM_667639_uff_E=172.66	-6.7	0	0
6lu7_EM_670971_uff_E=211.83	-5.8	0	0
6lu7_EM_689043_uff_E=98.60	-5.8	0	0
6lu7_EM_689075_uff_E=109.04	-5.5	0	0
6lu7_EM_1201518_uff_E=477.10	-5.3	0	0
6lu7_EM_1268096_uff_E=550.14	-7.5	0	0
6lu7_EM_1268142_uff_E=184.78	-6	0	0
6lu7_EM_1548883_uff_E=248.44	-5.8	0	0
6lu7_EM_1549108_uff_E=157.02	-5.5	0	0
6lu7_EM_1742210_uff_E=2180.22	-6	0	0
6lu7_EM_1794427_uff_E=254.42	-6.9	0	0
6lu7_EM_2113884_uff_E=484.54	-5.6	0	0
6lu7_EM_3000341_uff_E=673.45	-6.2	0	0
6lu7_EM_3001662_uff_E=918.86	-6.9	0	0
6lu7_EM_3010930_uff_E=646.91	-7.3	0	0
6lu7_EM_3034112_uff_E=2310.32	-7.2	0	0
6lu7_EM_3035567_uff_E=428.72	-6.5	0	0
6lu7_EM_3037048_uff_E=550.54	-6.1	0	0
6lu7_EM_3037151_uff_E=509.55	-6.7	0	0
6lu7_EM_3037448_uff_E=538.47	-6.1	0	0
6lu7_EM_3037884_uff_E=522.37	-6.7	0	0
6lu7_EM_3037997_uff_E=478.47	-6.7	0	0
6lu7_EM_3039336_uff_E=490.68	-6.7	0	0
6lu7_EM_3050539_uff_E=499.66	-6.6	0	0
6lu7_EM_3080632_uff_E=630.00	-6.5	0	0
6lu7_EM_3082134_uff_E=539.38	-6.7	0	0
6lu7_EM_3082494_uff_E=1012.62	-7.5	0	0
6lu7_EM_3083575_uff_E=259.76	-7	0	0
6lu7_EM_3083592_uff_E=514.46	-6.5	0	0
6lu7_EM_3083983_uff_E=442.13	-7.3	0	0
6lu7_EM_3084224_uff_E=555.47	-7.1	0	0
6lu7_EM_3084326_uff_E=609.20	-7.3	0	0
6lu7_EM_3084713_uff_E=539.21	-7.2	0	0
6lu7_EM_3084765_uff_E=575.31	-7.1	0	0
6lu7_EM_3084770_uff_E=502.76	-7.4	0	0
6lu7_EM_3085457_uff_E=639.83	-7.3	0	0
6lu7_EM_3086461_uff_E=4896.28	-7.5	0	0
6lu7_EM_3477029_uff_E=168.39	-6.3	0	0
6lu7_EM_5241825_uff_E=447.45	-6.7	0	0
6lu7_EM_5270605_uff_E=938.06	-6.8	0	0

6lu7_EM_5273569_uff_E=194.54	-6.1	0	0
6lu7_EM_5276890_uff_E=308.17	-4.2	0	0
6lu7_EM_5280343_uff_E=380.43	-7.5	0	0
6lu7_EM_5280372_uff_E=406.64	-6.5	0	0
6lu7_EM_5280373_uff_E=372.64	-7.3	0	0
6lu7_EM_5280378_uff_E=337.60	-7.1	0	0
6lu7_EM_5280385_uff_E=358.68	-5.6	0	0
6lu7_EM_5280435_uff_E=165.89	-4.5	0	0
6lu7_EM_5280443_uff_E=233.26	-7.3	0	0
6lu7_EM_5280445_uff_E=242.10	-6.7	0	0
6lu7_EM_5280448_uff_E=410.89	-5.5	0	0
6lu7_EM_5280450_uff_E=147.99	-5.4	0	0
6lu7_EM_5280459_uff_E=587.34	-7.3	0	0
6lu7_EM_5280460_uff_E=186.56	-6	0	0
6lu7_EM_5280462_uff_E=222.28	-5.7	0	0
6lu7_EM_5280536_uff_E=169.29	-5.4	0	0
6lu7_EM_5280569_uff_E=104.71	-6.5	0	0
6lu7_EM_5280633_uff_E=251.74	-6.9	0	0
6lu7_EM_5280637_uff_E=456.82	-6.2	0	0
6lu7_EM_5280644_uff_E=94.22	-5.2	0	0
6lu7_EM_5280666_uff_E=324.01	-7.2	0	0
6lu7_EM_5280703_uff_E=2512.28	-5.7	0	0
6lu7_EM_5280781_uff_E=577.00	-6.7	0	0
6lu7_EM_5280794_uff_E=546.19	-7.3	0	0
6lu7_EM_5281600_uff_E=626.01	-7.4	0	0
6lu7_EM_5281607_uff_E=229.60	-7.4	0	0
6lu7_EM_5281617_uff_E=247.18	-7	0	0
6lu7_EM_5281623_uff_E=224.59	-6.9	0	0
6lu7_EM_5281643_uff_E=608.46	-6.7	0	0
6lu7_EM_5281649_uff_E=365.00	-7.4	0	0
6lu7_EM_5281654_uff_E=450.92	-7.5	0	0
6lu7_EM_5281659_uff_E=405.71	-5.7	0	0
6lu7_EM_5281662_uff_E=420.82	-7.5	0	0
6lu7_EM_5281665_uff_E=241.42	-7.4	0	0
6lu7_EM_5281666_uff_E=378.68	-6.9	0	0
6lu7_EM_5281672_uff_E=388.01	-7.5	0	0
6lu7_EM_5281673_uff_E=592.73	-7.2	0	0
6lu7_EM_5281674_uff_E=238.34	-7.4	0	0
6lu7_EM_5281696_uff_E=737.84	-7.2	0	0
6lu7_EM_5281699_uff_E=450.91	-7.1	0	0
6lu7_EM_5281703_uff_E=315.73	-6.8	0	0
6lu7_EM_5281704_uff_E=426.96	-7.3	0	0
6lu7_EM_5281706_uff_E=383.08	-7.1	0	0
6lu7_EM_5281708_uff_E=321.26	-7.2	0	0
6lu7_EM_5281718_uff_E=365.16	-7.5	0	0
6lu7_EM_5281750_uff_E=452.19	-7.1	0	0
6lu7_EM_5281752_uff_E=535.71	-7	0	0
6lu7_EM_5281756_uff_E=488.53	-6.6	0	0
6lu7_EM_5281762_uff_E=260.55	-7.3	0	0
6lu7_EM_5281764_uff_E=272.33	-7.1	0	0
6lu7_EM_5281766_uff_E=255.77	-6.9	0	0
6lu7_EM_5281781_uff_E=464.25	-6.9	0	0
6lu7_EM_5281800_uff_E=559.11	-6.6	0	0
6lu7_EM_5281801_uff_E=363.65	-7.4	0	0
6lu7_EM_5281803_uff_E=446.60	-7.2	0	0
6lu7_EM_5281804_uff_E=373.68	-6.8	0	0
6lu7_EM_5281805_uff_E=483.40	-7.1	0	0
6lu7_EM_5281807_uff_E=524.81	-7.3	0	0
6lu7_EM_5281810_uff_E=643.16	-7.4	0	0
6lu7_EM_5281811_uff_E=448.75	-7	0	0
6lu7_EM_5281855_uff_E=227.58	-7.5	0	0
6lu7_EM_5282073_uff_E=197.42	-7.5	0	0
6lu7_EM_5282102_uff_E=602.48	-7.2	0	0
6lu7_EM_5282149_uff_E=605.92	-6.2	0	0
6lu7_EM_5282155_uff_E=788.62	-6	0	0
6lu7_EM_5282737_uff_E=47.52	-4.9	0	0

6lu7_EM_5282743_uff_E=59.81	-4.7	0	0
6lu7_EM_5282761_uff_E=74.80	-4.3	0	0
6lu7_EM_5283324_uff_E=23.87	-4	0	0
6lu7_EM_5283335_uff_E=24.66	-3.9	0	0
6lu7_EM_5283349_uff_E=30.78	-4.2	0	0
6lu7_EM_5283384_uff_E=76.41	-4.7	0	0
6lu7_EM_5284421_uff_E=115.86	-4.3	0	0
6lu7_EM_5284499_uff_E=95.04	-4.2	0	0
6lu7_EM_5280802_uff_E=128.00	-5.3	0	0
6lu7_EM_5280804_uff_E=610.61	-6.8	0	0
6lu7_EM_5280805_uff_E=751.59	-5	0	0
6lu7_EM_5280863_uff_E=362.50	-7.4	0	0
6lu7_EM_5280899_uff_E=704.15	-7.5	0	0
6lu7_EM_5280906_uff_E=466.78	-6.9	0	0
6lu7_EM_5280933_uff_E=157.30	-4.9	0	0
6lu7_EM_5280934_uff_E=142.21	-5.4	0	0
6lu7_EM_5280961_uff_E=356.74	-7.3	0	0
6lu7_EM_5281117_uff_E=147.06	-4.8	0	0
6lu7_EM_5281119_uff_E=95.80	-4.8	0	0
6lu7_EM_5281125_uff_E=103.73	-4.6	0	0
6lu7_EM_5281220_uff_E=505.22	-7	0	0
6lu7_EM_5281223_uff_E=2122.76	-7.4	0	0
6lu7_EM_5281234_uff_E=623.80	-7.1	0	0
6lu7_EM_5281235_uff_E=709.09	-7.4	0	0
6lu7_EM_5281243_uff_E=655.10	-7.5	0	0
6lu7_EM_5281247_uff_E=2418.43	-7.4	0	0
6lu7_EM_5281377_uff_E=560.81	-7.3	0	0
6lu7_EM_5281404_uff_E=350.13	-5.9	0	0
6lu7_EM_5281408_uff_E=550.96	-6.8	0	0
6lu7_EM_5281416_uff_E=104.17	-6.5	0	0
6lu7_EM_5281426_uff_E=97.00	-6	0	0
6lu7_EM_5281514_uff_E=848.59	-5.8	0	0
6lu7_EM_5281515_uff_E=740.89	-5.9	0	0
6lu7_EM_5281516_uff_E=164.36	-5.1	0	0
6lu7_EM_5281520_uff_E=195.77	-5.9	0	0
6lu7_EM_5281522_uff_E=720.33	-5.9	0	0
6lu7_EM_5281553_uff_E=113.15	-4.7	0	0
6lu7_EM_5284507_uff_E=158.07	-5	0	0
6lu7_EM_5312508_uff_E=148.38	-5.1	0	0
6lu7_EM_5317025_uff_E=649.05	-7.2	0	0
6lu7_EM_5317238_uff_E=109.82	-5.6	0	0
6lu7_EM_5317303_uff_E=246.15	-5.8	0	0
6lu7_EM_5317306_uff_E=280.44	-6.6	0	0
6lu7_EM_5317570_uff_E=260.17	-5.9	0	0
6lu7_EM_5317750_uff_E=411.31	-7.5	0	0
6lu7_EM_5317844_uff_E=261.31	-6.2	0	0
6lu7_EM_5318042_uff_E=33.39	-4.1	0	0
6lu7_EM_5318151_uff_E=1433.79	-6.6	0	0
6lu7_EM_5318267_uff_E=589.28	-7.2	0	0
6lu7_EM_5318358_uff_E=444.69	-6.6	0	0
6lu7_EM_5318565_uff_E=274.44	-5.7	0	0
6lu7_EM_5318599_uff_E=38.64	-4	0	0
6lu7_EM_5318767_uff_E=736.07	-6.7	0	0
6lu7_EM_5319292_uff_E=591.85	-7.3	0	0
6lu7_EM_5319322_uff_E=576.06	-8.3	0	0
6lu7_EM_5319706_uff_E=138.20	-4.9	0	0
6lu7_EM_5320351_uff_E=780.08	-6.7	0	0
6lu7_EM_5320686_uff_E=664.98	-6.3	0	0
6lu7_EM_5320863_uff_E=600.81	-7.5	0	0
6lu7_EM_5321205_uff_E=440.54	-7.3	0	0
6lu7_EM_5321398_uff_E=560.16	-7.5	0	0
6lu7_EM_5321656_uff_E=874.19	-7	0	0
6lu7_EM_5321825_uff_E=270.36	-6.1	0	0
6lu7_EM_5321919_uff_E=1187.31	-7.5	0	0
6lu7_EM_5321920_uff_E=993.69	-7.4	0	0
6lu7_EM_5321977_uff_E=353.12	-6.5	0	0

6lu7_EM_5321980_uff_E=270.56	-6.8	0	0
6lu7_EM_5352470_uff_E=1651.26	-5.9	0	0
6lu7_EM_5352973_uff_E=47.62	-4.5	0	0
6lu7_EM_5353015_uff_E=242.20	-5.8	0	0
6lu7_EM_5357283_uff_E=187.18	-5.5	0	0
6lu7_EM_5362793_uff_E=111.38	-5	0	0
6lu7_EM_5362863_uff_E=137.15	-4.1	0	0
6lu7_EM_5362876_uff_E=119.24	-5.2	0	0
6lu7_EM_5362885_uff_E=683.67	-6.1	0	0
6lu7_EM_5363249_uff_E=54.76	-4.6	0	0
6lu7_EM_5363388_uff_E=62.04	-4	0	0
6lu7_EM_5363734_uff_E=1632.70	-5.8	0	0
6lu7_EM_5364471_uff_E=74.84	-3.9	0	0
6lu7_EM_5364759_uff_E=57.67	-4.4	0	0
6lu7_EM_5365004_uff_E=50.96	-4.4	0	0
6lu7_EM_5365582_uff_E=84.64	-4.6	0	0
6lu7_EM_5365585_uff_E=79.49	-4.3	0	0
6lu7_EM_5365667_uff_E=95.06	-4	0	0
6lu7_EM_5365678_uff_E=65.99	-4.4	0	0
6lu7_EM_5366078_uff_E=337.57	-5.4	0	0
6lu7_EM_5367706_uff_E=112.03	-4.7	0	0
6lu7_EM_5367785_uff_E=150.45	-5.8	0	0
6lu7_EM_5368460_uff_E=213.29	-4.7	0	0
6lu7_EM_5375252_uff_E=140.97	-6	0	0
6lu7_EM_5378284_uff_E=389.84	-6.7	0	0
6lu7_EM_5380876_uff_E=505.90	-6.9	0	0
6lu7_EM_5383438_uff_E=608.53	-6.8	0	0
6lu7_EM_5384417_uff_E=729.61	-6.9	0	0
6lu7_EM_5384527_uff_E=521.35	-6.7	0	0
6lu7_EM_5459840_uff_E=741.05	-7.5	0	0
6lu7_EM_5462193_uff_E=632.32	-4.8	0	0
6lu7_EM_5462912_uff_E=86.58	-4.6	0	0
6lu7_EM_5481646_uff_E=527.88	-7.2	0	0
6lu7_EM_5481663_uff_E=826.93	-7	0	0
6lu7_EM_5481882_uff_E=586.70	-6.7	0	0
6lu7_EM_5484202_uff_E=569.80	-6.3	0	0
6lu7_EM_5486199_uff_E=842.59	-5.6	0	0
6lu7_EM_5490351_uff_E=758.81	-6.8	0	0
6lu7_EM_5742590_uff_E=785.84	-7.2	0	0
6lu7_EM_5835713_uff_E=613.39	-6.7	0	0
6lu7_EM_6100671_uff_E=968.50	-7.2	0	0
6lu7_EM_6143289_uff_E=121.55	-5.3	0	0
6lu7_EM_6325460_uff_E=827.44	-7.2	0	0
6lu7_EM_6326060_uff_E=692.55	-6.1	0	0
6lu7_EM_6384256_uff_E=709.09	-7.4	0	0
6lu7_EM_6421261_uff_E=202.20	-5	0	0
6lu7_EM_6423815_uff_E=214.81	-5	0	0
6lu7_EM_6427091_uff_E=149.30	-5.8	0	0
6lu7_EM_6428020_uff_E=376.38	-5.7	0	0
6lu7_EM_6429077_uff_E=180.69	-5.9	0	0
6lu7_EM_6429302_uff_E=713.72	-5.6	0	0
6lu7_EM_6431456_uff_E=209.47	-6.3	0	0
6lu7_EM_6432005_uff_E=264.33	-6.1	0	0
6lu7_EM_6432312_uff_E=179.25	-5.3	0	0
6lu7_EM_6432404_uff_E=194.04	-6.3	0	0
6lu7_EM_6432648_uff_E=217.68	-6	0	0
6lu7_EM_6438572_uff_E=936.53	-6	0	0
6lu7_EM_6439187_uff_E=395.09	-6.7	0	0
6lu7_EM_6441416_uff_E=485.86	-7.2	0	0
6lu7_EM_6442194_uff_E=520.30	-7.1	0	0
6lu7_EM_6442229_uff_E=752.25	-7.3	0	0
6lu7_EM_6442619_uff_E=523.70	-7.3	0	0
6lu7_EM_6442694_uff_E=571.67	-7.1	0	0
6lu7_EM_6443046_uff_E=544.56	-7	0	0
6lu7_EM_6450452_uff_E=116.52	-4.4	0	0
6lu7_EM_6452086_uff_E=220.04	-5.8	0	0

6lu7_EM_6452639_uff_E=358.36	-7.4	0	0
6lu7_EM_6453213_uff_E=79.30	-4.8	0	0
6lu7_EM_6474309_uff_E=311.67	-6.9	0	0
6lu7_EM_6474310_uff_E=607.28	-5.7	0	0
6lu7_EM_6476333_uff_E=563.20	-5	0	0
6lu7_EM_6508206_uff_E=247.53	-6.2	0	0
6lu7_EM_6737485_uff_E=459.52	-6.1	0	0
6lu7_EM_6857493_uff_E=456.55	-6.8	0	0
6lu7_EM_6912281_uff_E=539.51	-7	0	0
6lu7_EM_6917970_uff_E=432.85	-7.1	0	0
6lu7_EM_6918391_uff_E=152.08	-5.7	0	0
6lu7_EM_6918774_uff_E=706.68	-5.2	0	0
6lu7_EM_9548595_uff_E=638.65	-6.9	0	0
6lu7_EM_9548665_uff_E=679.09	-6	0	0
6lu7_EM_9548846_uff_E=550.59	-6.7	0	0
6lu7_EM_9796304_uff_E=139.93	-5.1	0	0
6lu7_EM_9798666_uff_E=249.33	-7	0	0
6lu7_EM_9846221_uff_E=733.38	-7.3	0	0
6lu7_EM_9846222_uff_E=864.24	-8.6	0	0
6lu7_EM_9851101_uff_E=2511.90	-7.3	0	0
6lu7_EM_9859098_uff_E=382.69	-5.3	0	0
6lu7_EM_9885603_uff_E=585.25	-7.2	0	0
6lu7_EM_9912297_uff_E=752.10	-7.4	0	0
6lu7_EM_9921439_uff_E=477.15	-6.6	0	0
6lu7_EM_9938773_uff_E=1007.31	-7.1	0	0
6lu7_EM_9945785_uff_E=249.76	-6.9	0	0
6lu7_EM_10049223_uff_E=787.06	-6.8	0	0
6lu7_EM_10085878_uff_E=382.01	-6.7	0	0
6lu7_EM_10091424_uff_E=592.80	-6.7	0	0
6lu7_EM_10133609_uff_E=323.37	-6.9	0	0
6lu7_EM_10163855_uff_E=736.81	-5.2	0	0
6lu7_EM_10345799_uff_E=781.14	-7.2	0	0
6lu7_EM_10354359_uff_E=407.91	-6.5	0	0
6lu7_EM_10358881_uff_E=364.10	-7.3	0	0
6lu7_EM_10366595_uff_E=423.11	-6.6	0	0
6lu7_EM_10380207_uff_E=406.42	-5.8	0	0
6lu7_EM_10383888_uff_E=395.25	-6	0	0
6lu7_EM_10398656_uff_E=220.84	-5.7	0	0
6lu7_EM_10433924_uff_E=618.00	-7.5	0	0
6lu7_EM_10434225_uff_E=812.32	-7.5	0	0
6lu7_EM_10445823_uff_E=383.37	-6.8	0	0
6lu7_EM_10456395_uff_E=459.05	-7.1	0	0
6lu7_EM_10456516_uff_E=386.16	-6.9	0	0
6lu7_EM_10466989_uff_E=137.98	-6.6	0	0
6lu7_EM_10475115_uff_E=586.19	-6.6	0	0
6lu7_EM_10476201_uff_E=560.06	-5.8	0	0
6lu7_EM_10478550_uff_E=954.35	-5.5	0	0
6lu7_EM_10538510_uff_E=463.27	-7.2	0	0
6lu7_EM_10589811_uff_E=373.48	-6.3	0	0
6lu7_EM_10675744_uff_E=989.61	-5.2	0	0
6lu7_EM_10704181_uff_E=1678.38	-5.8	0	0
6lu7_EM_10713200_uff_E=422.84	-7.1	0	0
6lu7_EM_10781192_uff_E=455.86	-6.9	0	0
6lu7_EM_10798883_uff_E=210.82	-6.1	0	0
6lu7_EM_10814237_uff_E=2111.36	-6.8	0	0
6lu7_EM_10850329_uff_E=582.66	-6	0	0
6lu7_EM_10856614_uff_E=191.36	-5.9	0	0
6lu7_EM_10906239_uff_E=886.50	-7.4	0	0
6lu7_EM_10948757_uff_E=524.86	-6.7	0	0
6lu7_EM_10962444_uff_E=1254.89	-5.8	0	0
6lu7_EM_11034432_uff_E=222.93	-6.7	0	0
6lu7_EM_11095734_uff_E=1698.37	-6.3	0	0
6lu7_EM_11102092_uff_E=951.99	-5.8	0	0
6lu7_EM_11113483_uff_E=1086.98	-6.4	0	0
6lu7_EM_11250133_uff_E=498.07	-7.1	0	0
6lu7_EM_11253808_uff_E=196.06	-7.4	0	0

6lu7_EM_11390848_uff_E=298.77	-6.9	0	0
6lu7_EM_11537361_uff_E=640.76	-7.5	0	0
6lu7_EM_11552547_uff_E=867.34	-7.4	0	0
6lu7_EM_11623165_uff_E=376.96	-7.4	0	0
6lu7_EM_11653181_uff_E=574.56	-7.2	0	0
6lu7_EM_11667940_uff_E=738.09	-7.2	0	0
6lu7_EM_11711453_uff_E=596.41	-7	0	0
6lu7_EM_11754080_uff_E=496.15	-7.2	0	0
6lu7_EM_11822566_uff_E=569.42	-5.8	0	0
6lu7_EM_11830551_uff_E=241.87	-6.1	0	0
6lu7_EM_11968944_uff_E=745.11	-4.4	0	0
6lu7_EM_11974586_uff_E=441.51	-5.4	0	0
6lu7_EM_12004512_uff_E=2292.11	-7.5	0	0
6lu7_EM_12004524_uff_E=882.98	-4.4	0	0
6lu7_EM_12019473_uff_E=921.34	-7.2	0	0
6lu7_EM_12019474_uff_E=884.47	-7.3	0	0
6lu7_EM_12302222_uff_E=230.48	-5.6	0	0
6lu7_EM_12302502_uff_E=518.77	-6.1	0	0
6lu7_EM_12303845_uff_E=593.93	-7.1	0	0
6lu7_EM_12304985_uff_E=1741.23	-6	0	0
6lu7_EM_12309865_uff_E=638.48	-5.1	0	0
6lu7_EM_12313019_uff_E=272.33	-6.9	0	0
6lu7_EM_12313020_uff_E=173.65	-6.3	0	0
6lu7_EM_12313665_uff_E=976.48	-6.6	0	0
6lu7_EM_12314884_uff_E=676.73	-6.6	0	0
6lu7_EM_12315515_uff_E=829.59	-7	0	0
6lu7_EM_12358846_uff_E=327.66	-7.4	0	0
6lu7_EM_13818582_uff_E=914.89	-6.1	0	0
6lu7_EM_13844274_uff_E=856.33	-5.9	0	0
6lu7_EM_13844288_uff_E=498.19	-6.9	0	0
6lu7_EM_13844292_uff_E=391.86	-6.6	0	0
6lu7_EM_13844293_uff_E=491.57	-7.4	0	0
6lu7_EM_13844295_uff_E=440.69	-6.6	0	0
6lu7_EM_13845970_uff_E=462.51	-6.9	0	0
6lu7_EM_13854255_uff_E=1730.77	-6.3	0	0
6lu7_EM_13889019_uff_E=430.82	-6.5	0	0
6lu7_EM_13889020_uff_E=397.64	-6.4	0	0
6lu7_EM_13889021_uff_E=458.73	-6.7	0	0
6lu7_EM_13965876_uff_E=498.53	-7.3	0	0
6lu7_EM_13967183_uff_E=920.30	-7.1	0	0
6lu7_EM_14019178_uff_E=1057.45	-5.5	0	0
6lu7_EM_14160302_uff_E=526.85	-7.3	0	0
6lu7_EM_14180789_uff_E=569.27	-6.6	0	0
6lu7_EM_14237625_uff_E=332.30	-6.4	0	0
6lu7_EM_14262868_uff_E=614.35	-6.9	0	0
6lu7_EM_14274765_uff_E=542.06	-7.1	0	0
6lu7_EM_14283236_uff_E=492.45	-7.4	0	0
6lu7_EM_14286954_uff_E=950.77	-6	0	0
6lu7_EM_14446178_uff_E=983.92	-6.9	0	0
6lu7_EM_14520970_uff_E=220.40	-7.4	0	0
6lu7_EM_14526072_uff_E=350.93	-6.8	0	0
6lu7_EM_14562693_uff_E=309.83	-7.2	0	0
6lu7_EM_14562696_uff_E=416.45	-6.7	0	0
6lu7_EM_14605164_uff_E=1024.75	-6.3	0	0
6lu7_EM_14691941_uff_E=306.85	-7.2	0	0
6lu7_EM_14707724_uff_E=2433.83	-5.7	0	0
6lu7_EM_14730796_uff_E=205.78	-6.8	0	0
6lu7_EM_14887327_uff_E=643.49	-6.4	0	0
6lu7_EM_14992067_uff_E=934.96	-7.2	0	0
6lu7_EM_14992071_uff_E=1000.37	-5.4	0	0
6lu7_EM_15011611_uff_E=492.71	-6.3	0	0
6lu7_EM_15226622_uff_E=880.19	-6.8	0	0
6lu7_EM_15275710_uff_E=1469.51	-5.9	0	0
6lu7_EM_15459518_uff_E=969.49	-5.2	0	0
6lu7_EM_15559638_uff_E=660.89	-7.2	0	0
6lu7_EM_15560252_uff_E=281.90	-6.1	0	0

6lu7_EM_15560332_uff_E=350.66	-5.8	0	0
6lu7_EM_15658444_uff_E=405.93	-6.3	0	0
6lu7_EM_15694364_uff_E=987.91	-7.3	0	0
6lu7_EM_15736564_uff_E=1991.94	-7.2	0	0
6lu7_EM_15767709_uff_E=2245.00	-6.8	0	0
6lu7_EM_15944778_uff_E=791.28	-6.2	0	0
6lu7_EM_15983991_uff_E=724.12	-7.5	0	0
6lu7_EM_16118969_uff_E=1079.65	-5.2	0	0
6lu7_EM_20055661_uff_E=689.02	-6.3	0	0
6lu7_EM_20056194_uff_E=1163.08	-5.7	0	0
6lu7_EM_21122581_uff_E=1085.20	-4.5	0	0
6lu7_EM_21582929_uff_E=944.80	-5.7	0	0
6lu7_EM_21582934_uff_E=902.33	-6.7	0	0
6lu7_EM_21582935_uff_E=936.36	-7.3	0	0
6lu7_EM_21594133_uff_E=1040.94	-7.2	0	0
6lu7_EM_21594201_uff_E=1029.71	-7.2	0	0
6lu7_EM_21603611_uff_E=902.79	-6.5	0	0
6lu7_EM_21636239_uff_E=541.75	-6.4	0	0
6lu7_EM_21637563_uff_E=505.53	-6.8	0	0
6lu7_EM_21637743_uff_E=1218.48	-5.6	0	0
6lu7_EM_21672545_uff_E=906.64	-7.4	0	0
6lu7_EM_21672546_uff_E=1010.22	-7.1	0	0
6lu7_EM_21673419_uff_E=1417.97	-7.3	0	0
6lu7_EM_21723831_uff_E=897.91	-7	0	0
6lu7_EM_21766881_uff_E=882.33	-2.7	0	0
6lu7_EM_22297418_uff_E=578.43	-7.2	0	0
6lu7_EM_22416599_uff_E=552.35	-4.3	0	0
6lu7_EM_22676887_uff_E=75.39	-4.2	0	0
6lu7_EM_24721165_uff_E=902.37	-7.1	0	0
6lu7_EM_24814354_uff_E=1729.57	-7.2	0	0
6lu7_EM_24970641_uff_E=575.07	-7.5	0	0
6lu7_EM_25104959_uff_E=1106.53	-5.6	0	0
6lu7_EM_25756094_uff_E=188.36	-6.6	0	0
6lu7_EM_25763835_uff_E=199.05	-6.6	0	0
6lu7_EM_27282457_uff_E=89.25	-5.3	0	0
6lu7_EM_42604340_uff_E=450.86	-6.1	0	0
6lu7_EM_42604341_uff_E=251.04	-6.2	0	0
6lu7_EM_42626427_uff_E=1462.85	-4.9	0	0
6lu7_EM_44147426_uff_E=439.01	-6.2	0	0
6lu7_EM_44241258_uff_E=227.54	-7	0	0
6lu7_EM_44241259_uff_E=252.51	-5.7	0	0
6lu7_EM_44256718_uff_E=759.59	-6	0	0
6lu7_EM_44257058_uff_E=432.88	-7.3	0	0
6lu7_EM_44257110_uff_E=499.57	-7.5	0	0
6lu7_EM_44257299_uff_E=639.23	-5.2	0	0
6lu7_EM_44257531_uff_E=499.51	-6.4	0	0
6lu7_EM_44258219_uff_E=623.44	-7.4	0	0
6lu7_EM_44421646_uff_E=861.15	-6.9	0	0
6lu7_EM_44421647_uff_E=928.75	-4.6	0	0
6lu7_EM_44557350_uff_E=1114.56	-5.3	0	0
6lu7_EM_44557351_uff_E=1096.26	-6.3	0	0
6lu7_EM_44557352_uff_E=1124.88	-5.2	0	0
6lu7_EM_44557353_uff_E=911.03	-6.8	0	0
6lu7_EM_44557413_uff_E=1042.55	-7.2	0	0
6lu7_EM_44557414_uff_E=977.87	-6.8	0	0
6lu7_EM_44557415_uff_E=1101.27	-6	0	0
6lu7_EM_44557416_uff_E=923.76	-7	0	0
6lu7_EM_44557481_uff_E=1010.81	-6	0	0
6lu7_EM_44557482_uff_E=1086.57	-5.2	0	0
6lu7_EM_44568160_uff_E=539.37	-6.7	0	0
6lu7_EM_44575701_uff_E=1079.47	-5.7	0	0
6lu7_EM_44578390_uff_E=512.12	-7.4	0	0
6lu7_EM_44583694_uff_E=1145.72	-7.5	0	0
6lu7_EM_44583695_uff_E=1254.22	-6.8	0	0
6lu7_EM_44584027_uff_E=876.14	-4.7	0	0
6lu7_EM_44584030_uff_E=783.21	-7.5	0	0

6lu7_EM_44593364_uff_E=1138.13	-6.6	0	0
6lu7_EM_44715841_uff_E=588.93	-6.6	0	0
6lu7_EM_45359677_uff_E=1123.83	-6.2	0	0
6lu7_EM_45482321_uff_E=786.78	-7.5	0	0
6lu7_EM_46174030_uff_E=435.37	-5.6	0	0
6lu7_EM_46882793_uff_E=957.76	-7.2	0	0
6lu7_EM_46939340_uff_E=710.54	-7.1	0	0
6lu7_EM_50909267_uff_E=667.89	-6.9	0	0
6lu7_EM_51003489_uff_E=1080.14	-6.2	0	0
6lu7_EM_52945930_uff_E=221.19	-7.3	0	0
6lu7_EM_54067425_uff_E=810.34	-6.4	0	0
6lu7_EM_54326635_uff_E=949.03	-7.2	0	0
6lu7_EM_54675810_uff_E=205.18	-5.3	0	0
6lu7_EM_54690297_uff_E=233.66	-5.9	0	0
6lu7_EM_56680033_uff_E=762.83	-4.7	0	0
6lu7_EM_56776306_uff_E=340.57	-7.1	0	0
6lu7_EM_56951689_uff_E=952.52	-7.3	0	0
6lu7_EM_57335470_uff_E=597.68	-5.6	0	0
6lu7_EM_58552693_uff_E=89.04	-5	0	0
6lu7_EM_70697882_uff_E=600.67	-5.8	0	0
6lu7_EM_70698280_uff_E=629.07	-6.2	0	0
6lu7_EM_71307329_uff_E=604.28	-7	0	0
6lu7_EM_71437983_uff_E=402.58	-7.3	0	0
6lu7_EM_71473354_uff_E=2606.44	-7.4	0	0
6lu7_EM_71473355_uff_E=756.85	-7.1	0	0
6lu7_EM_71473390_uff_E=971.96	-5.8	0	0
6lu7_EM_73348891_uff_E=903.83	-7.1	0	0
6lu7_EM_75202444_uff_E=400.94	-7.5	0	0
6lu7_EM_76327123_uff_E=504.95	-7.1	0	0
6lu7_EM_76330776_uff_E=489.75	-6.5	0	0
6lu7_EM_76972524_uff_E=1077.68	-4.2	0	0
6lu7_EM_85350942_uff_E=3273.69	-5.7	0	0
6lu7_EM_90474067_uff_E=259.81	-6.9	0	0
6lu7_EM_91227631_uff_E=331.76	-7.3	0	0
6lu7_EM_91864462_uff_E=1004.27	-6	0	0
6lu7_EM_91895373_uff_E=589.37	-7.2	0	0

6lu7_EM_91895456_uff_E=964.43	-6.3	0	0
6lu7_EM_92016157_uff_E=497.42	-6.2	0	0
6lu7_EM_92135690_uff_E=332.88	-6.6	0	0
6lu7_EM_92469142_uff_E=254.92	-5.6	0	0
6lu7_EM_100929735_uff_E=398.11	-6.8	0	0
6lu7_EM_100967916_uff_E=1061.99	-4.7	0	0
6lu7_EM_100968221_uff_E=750.87	-5.5	0	0
6lu7_EM_100991413_uff_E=938.63	-6.6	0	0
6lu7_EM_101051955_uff_E=818.70	-4.2	0	0
6lu7_EM_101117774_uff_E=807.85	-6.6	0	0
6lu7_EM_101277340_uff_E=1021.77	-5.6	0	0
6lu7_EM_101280173_uff_E=850.66	-7.3	0	0
6lu7_EM_101281096_uff_E=800.79	-5.9	0	0
6lu7_EM_101281312_uff_E=748.65	-7.1	0	0
6lu7_EM_101289764_uff_E=820.21	-7.4	0	0
6lu7_EM_101304457_uff_E=445.10	-7	0	0
6lu7_EM_101599479_uff_E=918.48	-7.4	0	0
6lu7_EM_101616678_uff_E=812.75	-7.3	0	0
6lu7_EM_101669621_uff_E=347.91	-6.8	0	0
6lu7_EM_101688442_uff_E=149.61	-5.6	0	0
6lu7_EM_101691231_uff_E=352.84	-6.8	0	0
6lu7_EM_101701119_uff_E=841.00	-7.2	0	0
6lu7_EM_101707493_uff_E=1080.37	-7.1	0	0
6lu7_EM_101711017_uff_E=1320.08	-6.3	0	0
6lu7_EM_101862378_uff_E=785.20	-6.5	0	0
6lu7_EM_101862379_uff_E=772.73	-6.5	0	0
6lu7_EM_101905232_uff_E=716.93	-7.2	0	0
6lu7_EM_101916323_uff_E=1328.12	-6.1	0	0
6lu7_EM_101926854_uff_E=1808.55	-5.7	0	0
6lu7_EM_101936045_uff_E=710.60	-6.2	0	0
6lu7_EM_101937309_uff_E=434.05	-7.2	0	0
6lu7_EM_102004681_uff_E=647.79	-8.8	0	0
6lu7_EM_102004748_uff_E=526.01	-6.3	0	0
6lu7_EM_102049211_uff_E=713.57	-7.3	0	0
6lu7_EM_102066925_uff_E=591.89	-5.6	0	0
6lu7_EM_102067840_uff_E=349.22	-6.7	0	0
6lu7_EM_102115826_uff_E=1286.26	-5.6	0	0
6lu7_EM_102121496_uff_E=965.18	-6.6	0	0
6lu7_EM_102121497_uff_E=1045.48	-5	0	0
6lu7_EM_102121498_uff_E=914.53	-5.6	0	0
6lu7_EM_102121829_uff_E=911.04	-5	0	0
6lu7_EM_102132636_uff_E=193.60	-6.9	0	0
6lu7_EM_102132637_uff_E=467.79	-7.3	0	0
6lu7_EM_102132638_uff_E=364.98	-6.7	0	0
6lu7_EM_102316663_uff_E=757.83	-7.1	0	0
6lu7_EM_102505446_uff_E=555.35	-7.2	0	0
6lu7_EM_122209598_uff_E=405.92	-6.8	0	0
6lu7_EM_122209599_uff_E=450.81	-7.2	0	0
6lu7_EM_122391444_uff_E=2621.43	-7.3	0	0
6lu7_EM_122391445_uff_E=2128.96	-7.5	0	0
6lu7_EM_122391446_uff_E=2064.48	-7	0	0
6lu7_EM_122391447_uff_E=2101.18	-7.1	0	0
6lu7_EM_129636740_uff_E=183.94	-5.5	0	0
6lu7_EM_129670266_uff_E=359.21	-7.1	0	0
6lu7_EM_129686415_uff_E=384.81	-7.1	0	0
6lu7_EM_129834462_uff_E=725.86	-7.4	0	0
6lu7_EM_129881881_uff_E=135.57	-5	0	0
6lu7_EM_130475803_uff_E=305.08	-6.5	0	0
6lu7_EM_130475831_uff_E=722.55	-7.2	0	0
6lu7_EM_132282051_uff_E=1919.93	-7.1	0	0
6lu7_EM_132556616_uff_E=682.39	-7.3	0	0
6lu7_EM_132556617_uff_E=606.96	-7.5	0	0
6lu7_EM_132556618_uff_E=546.73	-7.3	0	0
6lu7_EM_134814038_uff_E=634.93	-7.5	0	0
6lu7_EM_135408753_uff_E=93.68	-5.7	0	0
6lu7_EM_137347660_uff_E=2285.95	-7.2	0	0
